# Dietary Factors Drive Volatile Organic Compound Exposure and Modulate Its Impact on Multi‐Dimensional Biological Aging

**DOI:** 10.1002/fsn3.71753

**Published:** 2026-04-13

**Authors:** Weitao Su, Yaoyu Hu, Jindong Zhao, Ming Yang, Jingtao Wu, Danfeng Wen, Zhiqi Lin, Jiaxin Zhao, Yanbing Li, Jiufeng Li, Ang Li

**Affiliations:** ^1^ Central Laboratory The Second Affiliated Hospital of Fujian Medical University Quanzhou Fujian China; ^2^ Center for Rare Diseases, State Key Laboratory of Complex, Severe, and Rare Diseases Peking Union Medical College Hospital, Chinese Academy of Medical Sciences Beijing China; ^3^ Department of Epidemiology and Biostatistics, Institute of Basic Medical Sciences, Chinese Academy of Medical Sciences School of Basic Medicine Peking Union Medical College Beijing China; ^4^ Department of Epidemiology and Statistics, School of Public Health Hebei Medical University Shijiazhuang China; ^5^ Hebei Key Laboratory of Environment and Human Health Shijiazhuang Hebei Province China; ^6^ Key Laboratory of Health Technology Assessment, National Health Commission of the People's Republic of China Fudan University Shanghai China; ^7^ Key Laboratory of Public Health Safety, Ministry of Education School of Public Health, Fudan University Shanghai China

**Keywords:** biological aging, dietary modification, exposure inequality, volatile organic compounds

## Abstract

Volatile organic compound (VOC) exposure is ubiquitous and linked to aging, but dietary contributions and effects on multi‐dimensional biological age (BA) indicators are unclear. This study investigated dietary drivers of urinary VOC metabolite (VOCM) concentrations, their association with multi‐dimensional BA, and whether diet modifies this relationship. This study analyzed 4,976 U.S. adults (2011–2020), measuring 16 urinary VOC metabolites (VOCMs) and four BA indicators: Klemera‐Doubal BA/phenotypic age acceleration (KDMAgeAccel/PhenoAgeAccel), allostatic load (AL), and homeostatic dysregulation (HD). Survey‐weighted generalized linear and logistic regression models were used to examine associations between 16 VOCMs and four BA indicators, assessing interaction with dietary patterns. VOCM concentrations were influenced by dietary factors, including dietary patterns, food groups, and nutrients. Multiple VOCMs, and their mixture, were positively associated with BA (e.g., VOCM mixture on KDMAgeAccel > 0: OR = 1.235, 95% CI: 1.097, 1.391). Specifically, 4HBeMA exhibited significant positive associations with KDMAgeAccel, AL, and PhenoAgeAccel. KDMAgeAccel emerged as the most sensitive BA indicator, significantly associated with eight VOCMs, with DHBMA showing the strongest correlation (KDMAgeAccel > 0: OR = 1.393, 95% CI: 1.129, 1.718). Healthy dietary patterns have protective effects against VOCMs‐related BA. Dietary factors are significant drivers of VOC exposure inequality. VOCM exposure is linked to multi‐dimensional BA, for which KDMAgeAccel is a sensitive biomarker. Healthier dietary patterns may protect against VOC‐related BA, suggesting a viable strategy for public health intervention.

## Graphical abstract

In 4976 U.S. adults from NHANES 2011–2020, dietary factors influenced urinary volatile organic compound metabolites (VOCMs) and modified their associations with multi‐dimensional biological aging. Multiple VOCMs were positively associated with biological age indicators, with KDMAgeAccel being the most sensitive marker. Healthier dietary patterns may attenuate VOC‐related biological aging, highlighting diet as a modifiable target to reduce exposure inequality and aging risk.

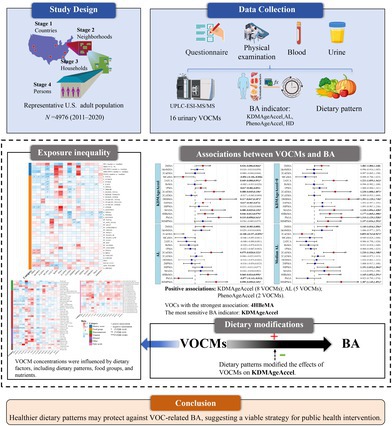

**Abbreviations**
2ATCA: 2‐Aminothiazoline‐4‐carboxylic acid
2CaEMA: N‐Acetyl‐S‐(2‐carbamoylethyl)‐L‐cysteine
2CoEMA: N‐Acetyl‐S‐(2‐carboxyethyl)‐L‐cysteine
2CyEMA: N‐Acetyl‐S‐(2‐cyanoethyl)‐L‐cysteine
2HPMA: N‐Acetyl‐S‐(2‐hydroxypropyl)‐L‐cysteine
2MHA: 2‐Methylhippuric acid
3&4MHA: 3‐ and 4‐Methylhippuric acid
3HMPMA: N‐Acetyl‐S‐(3‐hydroxypropyl‐1‐methyl)‐L‐cysteine
3HPMA: N‐Acetyl‐S‐(3‐hydroxypropyl)‐L‐cysteine
4HBeMA: N‐Acetyl‐S‐(4‐hydroxy‐2‐butenyl)‐L‐cysteine
AHEI‐2010: Alternative Healthy Eating Index‐2010
AL: Allostatic load
BA: Biological age
BzMA: N‐Acetyl‐S‐(benzyl)‐L‐cysteine
CDC: Centers for Disease Control and Prevention
CYP450: Cytochrome P450
DASHI: Dietary Approaches to Stop Hypertension Index
DHBMA: N‐Acetyl‐S‐(3,4‐dihydroxybutyl)‐L‐cysteine
DII: Dietary Inflammatory Index
DNAmPhenoAge: DNA methylation PhenoAge
ERK: Extracellular signal‐regulated kinase
FNDDS: Food and Nutrient Database for Dietary Studies
GM: Geometric mean
GST: Glutathione S‐transferase theta
HD: Homeostatic dysregulation
HEI‐2020: Healthy Eating Index‐2020
IGF‐1: Insulin‐like growth factor 1
KDMAgeAccel: Klemera‐Doubal biological age acceleration
LLOD: Lower limit of detection
MADA: Mandelic acid
MCaMA: N‐Acetyl‐S‐(N‐methylcarbamoyl)‐L‐cysteine
MEDI: Mediterranean Diet Index
MET: Metabolic equivalent of task
NF‐κB: Nuclear factor kappa B
NHANES: National Health and Nutrition Examination Survey
PA: Phenotypic age
PhenoAgeAccel: Phenotypic age acceleration
PhGA: Phenylglyoxylic acid
PIR: Poverty‐to‐income ratio
SMRM: Scheduled Multiple Reaction Monitoring
SRF: Serum response factor
UPLC‐ESI‐MS/MS: Ultra‐performance liquid chromatography coupled with electrospray tandem mass spectrometry
VOCMs: Volatile organic compound metabolites
VOCs: Volatile organic compounds
WBC: White blood cell

## 1 Introduction

Volatile organic compounds (VOCs), ubiquitous pollutants from industrial and domestic sources, vehicular emissions, and tobacco smoke, pose substantial health risks via inhalation, dermal contact, and dietary intake (Drabińska et al. 2021). Following exposure, VOCs undergo biotransformation via cytochrome P450 (CYP450) enzymes and phase II conjugation reactions, producing volatile organic compound metabolites (VOCMs) which serve as reliable internal exposure biomarkers (Hu et al. 2022; Shen et al. 2023). The activity of these hepatic enzymes is highly susceptible to dietary modulation. Both macronutrient composition and bioactive compounds—such as flavonoids and cruciferous vegetables—can induce or competitively inhibit specific CYP450 isoforms (Eagles et al. 2020; Niederberger and Parnham 2021), thereby altering the rate and profile of VOCM generation.

Regulatory actions in the United States, such as the Clean Air Act and subsequent federal standards, have significantly reduced ambient VOC emissions (Gil‐Alana and Solarin 2018; United States Environmental Protection Agency 2025). Significant exposure disparities persist across the population, driven by demographic, socioeconomic, and lifestyle factors. For example, females and Black individuals exhibited higher levels of specific VOCMs, potentially due to metabolic or environmental differences (St Helen et al. 2021; Woo et al. 2019). High‐risk occupational settings and urban versus rural environments further exacerbate these inequities (Chaiklieng et al. 2019). Furthermore, these demographic disparities may intersect with nutritional inequities; for instance, research indicates that Black populations often experience poorer diet quality (Pourmontaseri and Khanmohammadi 2024), potentially compounding their exposure risks. However, dietary intake's contribution to the overall human VOC body burden is frequently underestimated. Food can become contaminated with VOCs and other pollutants through various environmental and anthropogenic pathways, including crop uptake from contaminated soil, water, and air deposition, as well as bioaccumulation in animal products (Eurofins Scientific 2026; United States Environmental Protection Agency 2026). A study screening market food samples for 21 different VOCs found chloroform to be the most prevalent (detected in 97% of samples), followed by toluene (95%), ethylbenzene (80%), o‐xylene (79%), and benzene (58%) (Cao et al. 2018). VOC concentrations also vary significantly by food type (Cao et al. 2016). Evidence from other exposures further supports the concept that dietary ingestion is the primary pathway of human exposure for certain classes of environmental contaminants (Campo et al. 2019). However, the influence of different dietary patterns (e.g., Dietary Inflammatory Index (DII) (Shivappa et al. 2014) and Mediterranean Diet Index (MEDI) (Stewart 2018)), nutrients, and food types on VOC concentrations has not been quantified and requires further research. A systematic understanding of these exposure inequities is crucial for identifying vulnerable populations and developing targeted public health interventions.

Previous studies have demonstrated that exposure to specific VOCs may activate key molecular mechanisms known as hallmarks of aging. Toxicological studies have shown that acrolein stimulates the extracellular signal‐regulated kinase (ERK) → serum response factor (SRF) → nuclear factor kappa B (NF‐κB) inflammatory signaling pathway in vascular smooth muscle cells, a critical axis implicated in vascular, neural, and dermal system deterioration (Newaz and Yousefipour 2013; Singh et al. 2024). Likewise, both animal and human studies have confirmed that 1,3‐butadiene exhibits genotoxicity; its reactive metabolites act as electrophiles that covalently bind to nucleophilic DNA sites, forming DNA adducts and impairing DNA repair capacity (Lewis et al. 2019). These disruptions in genomic integrity and stability are known contributors to the aging processes (López‐Otín et al. 2023). However, most studies have focused on individual VOCs and aging mechanisms. Aging is a multisystem process characterized by progressive functional decline across physiological domains (López‐Otín et al. 2023). This complexity raises a crucial question: how can we feasibly and comprehensively quantify aging and evaluate the impact of numerous VOCs on aging?

Biological age (BA), which reflects an individual's functional and physiological state, assesses aging more accurately than chronological age (Hamczyk et al. 2020). Several BA indexes have been developed, including Klemera‐Doubal biological age (KDM‐BA) acceleration (KDMAgeAccel) (Klemera and Doubal 2006), phenotypic age (PA) acceleration (PhenoAgeAccel) (Levine et al. 2018), homeostatic dysregulation (HD) (Cohen et al. 2013), and allostatic load (AL) (Duong et al. 2017). Each captures distinct dimensions of aging—physiological dysregulation, mortality, physiological homeostasis, and cumulative stress burden. Yet, it remains uncertain which of these indexes are most responsive to VOC exposure, highlighting the need for population‐level studies linking VOCMs to BA. Given that dietary intake may constitute a significant source of VOC exposure, research into dietary strategies to reduce exposure and mitigate its impact on BA is crucial. Plant phenolic compounds have demonstrated efficacy in reducing cellular oxidative stress and inflammation from various air pollutants (Poljšak and Fink 2014). Diets abundant in these compounds offer an efficient protective approach (Nobile et al. 2021). Antioxidant‐rich and anti‐inflammatory dietary patterns may mitigate aging‐related damage (Tan et al. 2018; X. Wang et al. 2024). Several composite dietary indexes integrate multiple food and nutrient components to evaluate dietary quality (Appel et al. 1997; Chiuve et al. 2012; Shams‐White et al. 2023; Shivappa et al. 2014; Stewart 2018). Understanding how these dietary patterns interact with VOCM levels and influence multi‐dimensional BA could guide effective, modifiable strategies to reduce disparities in environmentally driven aging.

This study uses data from a representative U.S. population over 10 years to: (1) identify dietary factors affecting VOCM concentrations; (2) evaluate associations between VOCMs and multi‐dimensional BA indicators to identify sensitive aging biomarkers; and (3) examine the role of dietary patterns in modifying these associations.

## 2 Materials and Methods

### 2.1 Study Population

This study used data from 4 cycles of the National Health and Nutrition Examination Survey (NHANES): 2011–2012, 2013–2014, 2015–2016, and 2017–2020. NHANES assesses the health and nutritional status of a nationally representative sample of US residents, using a complex, multistage probability sampling design. A detailed description of the NHANES study design is available on the Centers for Disease Control and Prevention (CDC) official website (https://www.cdc.gov/nchs/nhanes/). This study was approved by National Center for Health Statistics (NCHS) Ethics Review Board (The institutional review board for each survey cycle was available at: https://www.cdc.gov/nchs/nhanes/irba98.htm). All participants provided written informed consent.

We excluded individuals under 20 years old, pregnant individuals, and those with missing data for all VOCMs and covariates. After exclusion, 4976 individuals were included. Additionally, missing values for each outcome were further excluded during the analysis of that specific outcome. The selection of study participants was shown in Figure S1. Specifically, the analytical sample sizes for KDMAgeAccel, AL, PhenoAgeAccel, and HD were 4976, 4827, 2804, and 2804, respectively. For PhenoAgeAccel and HD, the analyses were restricted to the 2015–2020 cycles due to the unavailability of C‐reactive protein (CRP) data in earlier cycles.

### 2.2 Measurements of Urinary VOCMs

Sample collection methods and urinary VOCM measurements have been described previously (Zhou et al. 2022). Briefly, urine specimens were collected at mobile examination centers, processed, stored at −20°C, and then shipped to the National Center for Environmental Health for testing. Urinary VOCMs were quantified using ultra‐performance liquid chromatography coupled with electrospray tandem mass spectrometry (UPLC‐ESI‐MS/MS) (Alwis et al. 2012). Urine samples and quality control specimens were diluted 10 times with 15 mM ammonium acetate. Internal standard solution was immediately added to each sample, followed by thorough mixing. Chromatographic separation of the analytes was accomplished using a UPLC system (e.g., Waters Acquity) equipped with a reversed‐phase C18 column (e.g., Acquity UPLC HSS T3). Detection of urinary VOCMs was performed using a triple quadrupole mass spectrometer (e.g., AB Sciex Triple Quad 5500) with an electrospray ion source under Scheduled Multiple Reaction Monitoring (SMRM) mode (Bhandari, Pirkle, & Centers for Disease Control and Prevention (CDC) 2017).

The lower limits of detection (LLOD) and detection rates for 30 VOCMs measured in 4 cycles (2011–2020) are shown in Table S1. We excluded 12 VOCMs not detected in all 4 cycles and 2 VOCMs with a detection rate below 60% (Table S1). Eventually, 16 VOCMs were included in our study, including 2‐methylhippuric acid (2MHA), 3‐methylhippuric acid and 4‐methylhippuric acid (3&4MHA), N‐acetyl‐S‐(2‐carbamoylethyl)‐L‐cysteine (2CaEMA), N‐acetyl‐S‐(N‐methylcarbamoyl)‐L‐cysteine (MCaMA), 2‐aminothiazoline‐4‐carboxylic acid (2ATCA), N‐acetyl‐S‐(benzyl)‐L‐cysteine (BzMA), N‐acetyl‐S‐(n‐propyl)‐L‐cysteine (1PMA), N‐acetyl‐S‐(2‐carboxyethyl)‐L‐cysteine (2CoEMA), N‐acetyl‐S‐(2‐cyanoethyl)‐L‐cysteine (2CyEMA), N‐acetyl‐S‐(3,4‐dihydroxybutyl)‐L‐cysteine (DHBMA), N‐acetyl‐S‐(2‐hydroxypropyl)‐L‐cysteine (2HPMA), N‐acetyl‐S‐(3‐hydroxypropyl)‐L‐cysteine (3HPMA), mandelic acid (MADA), N‐acetyl‐S‐(4‐hydroxy‐2‐butenyl)‐L‐cysteine (4HBeMA), phenylglyoxylic acid (PhGA), and N‐acetyl‐S‐(3‐hydroxypropyl‐1‐methyl)‐L‐cysteine (3HMPMA). Concentrations of VOCMs below the LLOD were replaced with the LLOD divided by the square root of 2.

### 2.3 Assessment of Multi‐Dimensional BA

In this study, we assessed multi‐dimensional biological age (BA) using data from NHANES, which included blood biochemistry, complete blood count, and physical examination data. We calculated four BA indicators: Klemera‐Doubal method Biological Age acceleration (KDMAgeAccel) (Klemera and Doubal 2006), PhenoAge acceleration (PhenoAgeAccel)(Levine et al. 2018), homeostatic dysregulation (HD) (Cohen et al. 2013), and allostatic load (AL) (Duong et al. 2017). KDMAgeAccel and PhenoAgeAccel were derived from the well‐established KDM‐BA and PhenoAge (PA) models, respectively, and were calculated as the residuals from regressing biological age on chronological age. These four indicators quantify aging‐related declines in system integrity and are robustly associated with morbidity and mortality risks (Belsky et al. 2015; Klemera and Doubal 2006; Levine and Crimmins 2018; Levine et al. 2018).

The clinical biomarkers used for these BA indicators are summarized in Table S2, covering five dimensions: the inflammation system, glucose and lipid metabolism, the cardiovascular system, kidney, and liver function. The calculations for KDM‐BA (underlying KDMAgeAccel) and AL include biomarkers from all five dimensions and allow for missing values. In contrast, PA (underlying PhenoAgeAccel) and HD include biomarkers from four dimensions (excluding the cardiovascular system) and do not allow for missing values. AL dichotomizes biomarkers by quantiles, whereas the other indicators use continuous variables, potentially capturing more detailed physiological information. The detailed calculation process for all BA indicators is shown in Supplementary Methods. Although DNA methylation epigenetic clocks are robust measures of BA, they were excluded because NHANES only assessed them during 1999–2002, lacking temporal overlap with urinary VOCM data (2011–2020).

Accelerated BA was defined for each indicator. For the age acceleration metrics, KDMAgeAccel > 0 or PhenoAgeAccel > 0 indicates that an individual is biologically older than their chronological age, suggesting an advanced BA process and elevated risks of disease and mortality (Liu et al. 2018). Individuals with HD or AL values above the median were categorized as having accelerated BA, indicating a greater risk of homeostatic disturbances and physiological health burden (Wang et al. 2024).

Due to the unavailability of CRP data from 2011 to 2014, PA and HD could only be calculated for the 2015–2020 cycles. To mitigate potential bias from this reduced sample size, we designated KDMAgeAccel and AL as the primary analyses, and PhenoAgeAccel and HD as the secondary analyses.

### 2.4 Dietary Data

Daily dietary intakes were evaluated using 24‐h dietary recall data. During the interview, participants were prompted to provide types and amounts of all foods and beverages consumed on the preceding day. The second dietary interview was conducted 3–10 days after the first face‐to‐face interview by telephone. Because NHANES dietary interviews are conducted across all days of the week, this data collection strategy captures both weekday and weekend dietary habits. Energy and nutrients for each food and beverage consumed were calculated using USDA's Food and Nutrient Database for Dietary Studies (FNDDS) for each NHANES cycle. The collected dietary recall data were integrated into the Food Patterns Equivalent Database (FPED), which classifies foods into 37 USDA Food Pattern Components by the USDA Food Composition Table. For single‐component foods, FPED directly distributes the food to the corresponding component. For multi‐ingredient foods containing multiple components, FPED uses standard recipe files to disaggregate these foods into their components. Dietary intakes of energy, nutrients, and food groups were calculated as the mean of 2‐day data if available; otherwise, data from the first day was used. Besides, several dietary indexes of high interest in research, including DII (Shivappa et al. 2014), MEDI (Stewart 2018), Healthy Eating Index‐2020 (HEI‐2020) (Shams‐White et al. 2023), Alternative Healthy Eating Index‐2010 (AHEI‐2010) (Chiuve et al. 2012), Dietary Approaches to Stop Hypertension Index (DASHI) (Appel et al. 1997), were used to comprehensively evaluate overall dietary quality based on daily intakes of nutrients and food groups. Specifically, the DII quantifies the inflammatory potential of a diet based on 45 nutritional parameters, with higher scores reflecting a stronger pro‐inflammatory and unhealthy profile. Conversely, the four healthy dietary indices generally score diets by rewarding the intake of protective foods (e.g., vegetables, fruits, whole grains, legumes, nuts, and seafood) while penalizing the consumption of detrimental items like red or processed meats and added sugars. Briefly, higher DII scores indicated a more pro‐inflammatory and unhealthy diet, while higher MEDI, HEI‐2020, AHEI‐2010 and DASHI scores indicated a higher quality and healthier diet. The calculation of all dietary indexes was performed using the R package “Dietaryindex” (Zhan et al. 2024). The components of the healthy dietary indices are summarized in Table S3.

### 2.5 Assessment of Covariates

Questionnaires recorded sociodemographic information and lifestyles. Body mass index (BMI) was determined by body weight in kilograms divided by height squared in meters (kg/m^2^). Poverty‐to‐income ratio (PIR) reflects socio‐economic status and estimates household income based on poverty standards. Smoking status was categorized into never smoker (those who had never smoked at least 100 cigarettes in life), former smoker (those who had smoked at least 100 cigarettes in life and now quit smoking), and current smoker (those who had smoked at least 100 cigarettes in life and now smoked cigarettes every day or sometimes). Drinking status was categorized into never and light drinker (≤ 2 drinks/day for male; ≤ 1 drinks/day for female), moderate drinker (3 drinks/day for male; 2 drinks/day for female), or heavy drinker (≥ 4 drinks/day for male; ≥ 3 drinks/day for female)(Zhang et al. 2023). Physical activity was quantified as metabolic equivalent of task (MET) minutes per week (MET‐minutes/week), calculated by multiplying activity duration and intensity‐specific MET score (Piercy et al. 2018). Total energy intake was calculated by mean calorie values in two‐day 24‐h dietary recalls.

### 2.6 Statistical Analysis

All analyses accounted for the complex sampling design of NHANES using appropriate nationally representative weights. We applied the weights from Subsample A (urinary VOCMs), which represented the smallest subset analyzed in our study. Per NHANES guidelines, we calculated new combined weights for the three 2‐year cycles (2011–2016) and the special 3.2‐year pre‐pandemic cycle (2017–2020) by proportionally adjusting them based on their temporal contributions to the overall 5.2‐year (2015–2020) or 9.2‐year (2011–2020) periods.

Continuous variables were expressed as survey‐weighted medians with interquartile ranges (Q25–Q75) due to skewed distributions, and between‐group differences were assessed using survey‐weighted Wilcoxon rank sum or Kruskal‐Wallis tests. Categorical variables were presented as frequencies with survey‐weighted percentages, and between‐group differences were assessed using survey‐weighted Pearson's chi‐squared tests.

Survey‐weighted generalized linear models were used to estimate regression coefficients (βs) and corresponding 95% confidence intervals (95% CIs) for associations of demographics, lifestyles, comorbidities, environmental, and dietary factors with VOCMs, as well as associations of VOCMs with continuous BA indicators (KDMAgeAccel, PhenoAgeAccel, HD, and AL). Survey‐weighted generalized logistic regression models were used to estimate odds ratios (ORs) and corresponding 95% CIs for associations of VOCMs with binary BA indicators. Because PhenoAgeAccel and HD were unavailable from 2011 to 2015, potentially biasing results, we prioritized KDMAgeAccel and AL as the main analysis, with PhenoAgeAccel and HD as secondary analyses. The combined effects of VOCMs mixture on BA indicators were assessed using weighted quantile g‐computation (qgcomp).

In our study, covariates in the model included: age (continuous, years), sex (male/female), BMI (continuous, kg/m^2^), race (Mexican American, other‐Hispanic, non‐Hispanic White, non‐Hispanic Black, and other races), marital status (never married, married, living with partner, widowed/divorced/separated), PIR (< 1.3, 1.3–3.5, and > 3.5), education level (less than high school, high school graduate, some college or AA degree, college graduate or above), smoking status (never, former and current), drinking status (never and light drinker, moderate drinker, heavy drinker), physical activity (continuous, MET‐minutes/week), self‐reported diabetes (no and yes), self‐reported hypertension (no and yes), self‐reported hyperlipidemia (no and yes), total energy intake (continuous, kcal) and urinary creatinine (continuous, mg/dL). All variables in the model exhibited variance inflation factor (VIF) values below 5, suggesting multicollinearity was unlikely (Table S4).

To explore the interactions between VOCMs and dietary indexes on continuous BA indicators, product terms of VOCMs and dietary indexes were included in linear regression models to assess potential significant interactions. Before all association analyses, due to skewed distribution, VOCMs were Ln‐transformed, and BA indicators (KDMAgeAccel, PhenoAgeAccel, HD, and AL) and dietary variables (including dietary indexes and food intake) were Z‐score transformed. The covariates mentioned above were adjusted for all regression analyses.

All statistical analyses were conducted using R version 4.3.1. The primary R packages used were “survey”, “rms”, “ggplot2”, and “qgcomp”. Statistical significance was defined as a 2‐tailed *p* < 0.05. To account for multiple comparisons, raw *p* values were adjusted using the Benjamini–Hochberg false discovery rate (FDR) method across all VOCMs separately for each dietary variable and BA indicator.

## 3 Results

### 3.1 Population Characteristics Among Delayed BA and Accelerated BA

The general characteristics of the participants are presented in Table 1. Among 4976 participants included in this study, the median age was 48 (33, 61) years and 50.2% were male. Notably, 2411 (48.5%) participants exhibited accelerated KDM‐BA. There were significant between‐group differences in all demographic variables (*p* < 0.05). Compared to the delayed KDM‐BA group, participants with accelerated KDM‐BA were more likely to be younger, female, non‐Hispanic Black, unmarried, and have higher BMI, lower education level, and lower PIR, and less likely to be non‐Hispanic White. Besides, participants with accelerated KDM‐BA had a higher proportion of current smokers, heavy drinkers, and participants with diabetes and hypertension, and higher BA indicators (all *p* < 0.05). All VOCMs, except for MCaMA, were significantly higher in the accelerated KDM‐BA group. For dietary indexes, participants with accelerated KDM‐BA had significantly higher DII scores (unhealthy) and lower scores on healthy dietary indexes, including MEDI, HEI‐2020, AHEI‐2010, and DASHI.

**TABLE 1:** General characteristics of subjects by KDMAgeAccel.

Characteristics	Total (*N* = 4976)	Delayed KDM‐BA (KDMAgeAccel ≤ 0, *N* = 2565)	Accelerated KDM‐BA (KDMAgeAccel > 0, *N* = 2411)	*p*
Demographics				
Age, years (median [IQR])	48.00 [33.00, 61.00]	48.30 [35.00, 61.00]	46.00 [31.77, 60.00]	**0.018**
BMI, kg/m^2^	28.30 [24.50, 33.00]	26.90 [23.60, 31.23]	30.10 [26.00, 35.40]	**< 0.001**
Sex, *n* (%)				**0.037**
Male	2566 (50.2)	1359 (52.2)	1207 (47.8)	
Female	2410 (49.8)	1206 (47.8)	1204 (52.2)	
Race, *n* (%)				**< 0.001**
Mexican American	584 (7.4)	296 (6.8)	288 (8.1)	
Other Hispanic	506 (6.0)	270 (6.0)	236 (6.1)	
Non‐Hispanic White	1980 (68.3)	1121 (72.1)	859 (63.6)	
Non‐Hispanic Black	1173 (10.5)	461 (7.5)	712 (14.1)	
Other Race—Including Multi‐Racial	733 (7.8)	417 (7.6)	316 (8.2)	
Education level, *n* (%)				**< 0.001**
Less than high school	921 (11.7)	483 (11.4)	438 (12.1)	
High school	1147 (23.9)	530 (21.3)	617 (27.1)	
Some college or AA degree	1579 (31.9)	762 (30.9)	817 (33.0)	
College graduate or above	1329 (32.5)	790 (36.3)	539 (27.8)	
Marital status, *n* (%)				**0.007**
Married/Living with Partner	2921 (63.3)	1580 (65.5)	1341 (60.7)	
Widowed/Divorced/Separated	1064 (18.4)	525 (17.7)	539 (19.4)	
Never married	991 (18.2)	460 (16.8)	531 (20.0)	
PIR category, *n* (%)				**0.001**
< 1.3	1505 (20.1)	714 (17.4)	791 (23.3)	
1.3–3.5	1860 (35.8)	933 (35.9)	927 (35.7)	
> 3.5	1611 (44.1)	918 (46.7)	693 (41.0)	
Lifestyles				
Smoking status, *n* (%)				**0.024**
Never	2783 (56.2)	1436 (56.1)	1347 (56.3)	
Former	1230 (26.1)	665 (27.7)	565 (24.2)	
Current	963 (17.7)	464 (16.3)	499 (19.5)	
Alcohol drinking status, *n* (%)				**0.003**
Never and light drinker	3164 (60.6)	1679 (62.5)	1485 (58.2)	
Moderate drinker	845 (18.5)	439 (19.4)	406 (17.4)	
Heavy drinker	967 (21.0)	447 (18.1)	520 (24.4)	
MET‐minutes/week	1600.00 [240.00, 5160.00]	1560.00 [360.00, 4920.00]	1614.60 [240.00, 5520.00]	0.520
Total energy intake (kcal)	2004.00 [1549.93, 2598.89]	2025.27 [1572.77, 2587.63]	1981.53 [1525.00, 2608.69]	0.476
Comorbidities				
Diabetes, *n* (%)	677 (10.7)	252 (7.1)	425 (15.1)	**< 0.001**
Hypertension, *n* (%)	1829 (32.6)	752 (26.2)	1077 (40.4)	**< 0.001**
Hyperlipidemia, *n* (%)	1822 (34.7)	897 (33.3)	925 (36.4)	0.172
Biological aging indicators				
KDMAgeAccel	−1.59 [−8.45, 6.45]	−7.67 [−12.94, −3.92]	7.52 [3.40, 13.80]	**< 0.001**
PhenoAgeAccel	0.66 [−1.86, 4.12]	−0.72 [−3.13, 1.68]	2.49 [−0.32, 5.92]	**< 0.001**
AL	0.20 [0.07, 0.33]	0.13 [0.05, 0.25]	0.30 [0.17, 0.43]	**< 0.001**
HD	3.12 [2.66, 3.81]	3.00 [2.52, 3.48]	3.37 [2.80, 4.14]	**< 0.001**
VOCMs				
2MHA, ng/mL	27.90 [12.30, 73.70]	25.20 [11.00, 70.60]	30.77 [14.30, 78.34]	**0.001**
3&4MHA, ng/mL	168.00 [70.95, 469.96]	160.00 [64.50, 447.56]	178.00 [79.58, 498.00]	**0.019**
2CaEMA, ng/mL	50.10 [26.40, 99.84]	47.20 [24.70, 93.25]	54.10 [29.10, 106.00]	**< 0.001**
MCaMA, ng/mL	154.00 [75.14, 315.00]	149.00 [72.20, 303.00]	159.00 [77.60, 333.00]	0.069
2ATCA, ng/mL	102.00 [48.00, 200.00]	87.64 [42.08, 171.00]	122.00 [56.60, 242.00]	**< 0.001**
BzMA, ng/mL	6.12 [3.18, 11.40]	5.78 [2.88, 10.90]	6.39 [3.51, 12.20]	**0.005**
1PMA, ng/mL	3.67 [1.33, 10.40]	3.39 [0.85, 9.39]	4.12 [1.56, 11.33]	**0.006**
2CoEMA, ng/mL	93.44 [47.16, 178.00]	79.50 [40.40, 159.79]	109.92 [58.80, 197.00]	**< 0.001**
2CyEMA, ng/mL	1.60 [0.76, 7.04]	1.45 [0.69, 5.75]	1.72 [0.86, 11.37]	**< 0.001**
DHBMA, ng/mL	318.00 [176.00, 515.00]	283.00 [155.00, 482.00]	361.00 [207.00, 555.00]	**< 0.001**
2HPMA, ng/mL	29.40 [15.60, 59.40]	27.00 [14.40, 56.07]	32.40 [17.70, 64.55]	**< 0.001**
3HPMA, ng/mL	237.00 [116.00, 472.00]	210.00 [104.00, 436.00]	264.00 [134.00, 518.00]	**< 0.001**
MADA, ng/mL	132.00 [72.00, 233.00]	118.00 [67.00, 215.00]	150.00 [82.77, 252.00]	**< 0.001**
4HBeMA, ng/mL	4.56 [2.40, 10.10]	4.12 [2.12, 8.55]	5.13 [2.81, 11.80]	**< 0.001**
PhGA, ng/mL	208.00 [117.00, 354.92]	190.00 [104.00, 334.00]	232.10 [133.49, 375.00]	**< 0.001**
3HMPMA, ng/mL	227.00 [118.00, 434.00]	195.00 [104.18, 394.85]	256.00 [140.21, 472.00]	**< 0.001**
Dietary index				
DII	1.07 [−0.23, 2.34]	0.89 [−0.39, 2.22]	1.32 [0.06, 2.52]	**< 0.001**
MEDI	3.50 [2.50, 4.50]	3.50 [2.50, 4.50]	3.00 [2.00, 4.00]	**< 0.001**
HEI‐2020	50.52 [42.23, 59.70]	51.95 [43.36, 61.39]	48.58 [41.09, 57.54]	**< 0.001**
AHEI‐2010	38.16 [30.51, 47.27]	39.79 [31.73, 48.73]	36.42 [29.51, 45.40]	**< 0.001**
DASHI	22.00 [18.50, 26.00]	22.50 [19.00, 27.00]	21.00 [18.00, 25.00]	**< 0.001**

*Note:* All continuous variables were expressed as weighted medians (25, 75th percentiles), categorical variables were presented as frequencies (weighted percentages).

Abbreviations: 1PMA, N‐acetyl‐S‐(n‐propyl)‐L‐cysteine; 2ATCA, 2‐aminothiazoline‐4‐carboxylic acid; 2CaEMA, N‐acetyl‐S‐(2‐carbamoylethyl)‐L‐cysteine; 2CoEMA, N‐acetyl‐S‐(2‐carboxyethyl)‐L‐cysteine; 2CyEMA, N‐acetyl‐S‐(2‐cyanoethyl)‐L‐cysteine; 2HPMA, N‐acetyl‐S‐(2‐hydroxypropyl)‐L‐cysteine; 2MHA, 2‐methylhippuric acid; 3&4MHA, 3‐ and 4‐methylhippuric acid; 3HMPMA, N‐acetyl‐S‐(3‐hydroxypropyl‐1‐methyl)‐L‐cysteine; 3HPMA, N‐acetyl‐S‐(3‐hydroxypropyl)‐L‐cysteine; 4HBeMA, N‐acetyl‐S‐(4‐hydroxy‐2‐butenyl)‐L‐cysteine; AHEI‐2010, Alternative Healthy Eating Index‐2010; AL, allostatic load; BzMA, N‐acetyl‐S‐(benzyl)‐L‐cysteine; DASHI, Dietary Approaches to Stop Hypertension Index; DHBMA, N‐acetyl‐S‐(3,4‐dihydroxybutyl)‐L‐cysteine; DII, Dietary Inflammatory Index; HD, homeostatic dysregulation; HEI‐2020, Healthy Eating Index‐2020; KDMAgeAccel, Klemera‐Doubal method biological age acceleration; MADA, mandelic acid; MCaMA, N‐acetyl‐S‐(N‐methylcarbamoyl)‐L‐cysteine; MEDI, Mediterranean Diet Index; MET, metabolic equivalent; PhenoAgeAccel, phenotypic age acceleration; PhGA, phenylglyoxylic acid; PIR, poverty‐to‐income ratio.

Detection rates and distribution of the included VOCMs are represented in Table S5. VOCM concentrations varied greatly; the top 5 by geometric mean (GM) were DHBMA (291.78 ng/mL), 3HPMA (248.81 ng/mL), 3HMPMA (246.96 ng/mL), PhGA (195.77 ng/mL), and 3&4MHA (182.79 ng/mL), while the bottom 5 VOCMs with the lowest GM concentrations were 2CyEMA (3.32 ng/mL), 1PMA (4.21 ng/mL), 4HBeMA (5.24 ng/mL), BzMA (6.27 ng/mL), and 2MHA (29.86 ng/mL). Spearman's rank correlation analysis revealed that the correlation coefficients ranged from 0.12 to 0.88 (Figure S2).

### 3.2 Dietary Factors Affecting VOCM Concentrations

**FIGURE 1:**
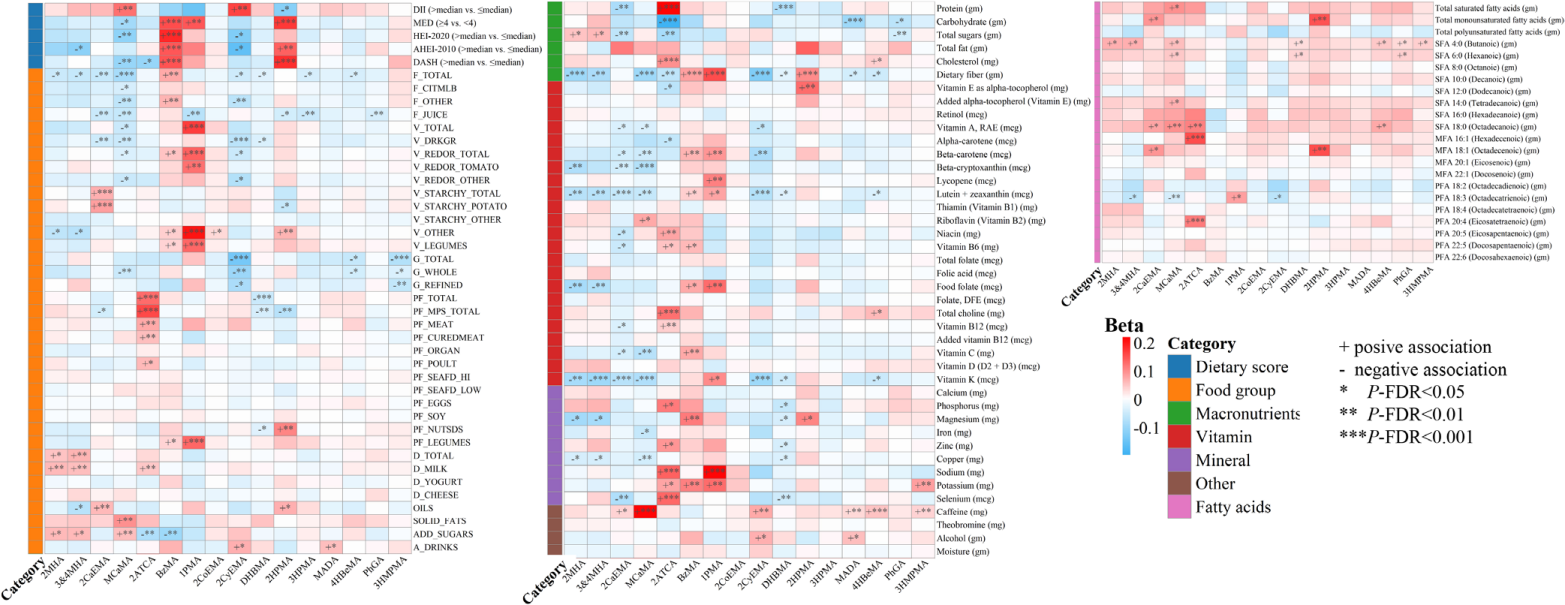
Dietary exposures related to VOCMs. Survey‐weighted multivariable linear regression models were used to evaluate the influencing factors of Ln‐transformed VOCM concentrations, adjusted for age, sex, BMI, race, marital status, education level, PIR, smoking status, drinking status, physical activity (MET‐minutes/week), total energy intake (kcal), diabetes, hypertension, hyperlipidemia, and urinary creatinine. FDR correction was applied to all *p* values across 16 VOCMs separately for each dietary variable. ***p‐FDR < 0.001, **p‐FDR < 0.01, *p‐FDR < 0.05. 1PMA, N‐acetyl‐S‐(n‐propyl)‐L‐cysteine; 2ATCA, 2‐aminothiazoline‐4‐carboxylic acid; 2CaEMA, N‐acetyl‐S‐(2‐carbamoylethyl)‐L‐cysteine; 2CoEMA, N‐acetyl‐S‐(2‐carboxyethyl)‐L‐cysteine; 2CyEMA, N‐acetyl‐S‐(2‐cyanoethyl)‐L‐cysteine; 2HPMA, N‐acetyl‐S‐(2‐hydroxypropyl)‐L‐cysteine; 2MHA, 2‐methylhippuric acid; 3&4MHA, 3‐ and 4‐methylhippuric acid; 3HMPMA, N‐acetyl‐S‐(3‐hydroxypropyl‐1‐methyl)‐L‐cysteine; 3HPMA, N‐acetyl‐S‐(3‐hydroxypropyl)‐L‐cysteine; 4HBeMA, N‐acetyl‐S‐(4‐hydroxy‐2‐butenyl)‐L‐cysteine; A_DRINKS, Alcoholic beverages (no. of drinks); ADD_SUGARS, Foods defined as added sugars (tsp. eq.); AHEI‐2010, Alternative Healthy Eating Index‐2010; BzMA, N‐acetyl‐S‐(benzyl)‐L‐cysteine; D_CHEESE, Cheese (cup eq.); D_MILK, Fluid milk and calcium fortified soy milk (cup eq.); D_TOTAL, Total milk, yogurt, cheese, and whey (cup eq.); D_YOGURT, Yogurt (cup eq.); DASHI, Dietary Approaches to Stop Hypertension Index; DHBMA, N‐acetyl‐S‐(3,4‐dihydroxybutyl)‐L‐cysteine; DII, Dietary Inflammatory Index; F_CITMLB, Intact fruits (whole or cut) of citrus, melons, and berries (cup eq.); F_JUICE, Fruit juices, citrus and non‐citrus (cup eq.); F_OTHER, Intact fruits (whole or cut); excluding citrus, melons, and berries (cup eq.); F_TOTAL, Total intact or cut fruits and fruit juices (cup eq.); G_REFINED, Refined or non‐whole grains (oz. eq.); G_TOTAL, Total whole and refined grains (oz. eq.); G_WHOLE, Whole grains (oz. eq.); HEI‐2020, Healthy Eating Index‐2020; MADA, mandelic acid; MCaMA, N‐acetyl‐S‐(N‐methylcarbamoyl)‐L‐cysteine; MEDI, Mediterranean Diet Index; OILS, Oils (grams); PF_CUREDMEAT, Cured/luncheon meat made from beef, pork, or poultry (oz. eq.); PF_EGGS, Eggs (chicken, duck, goose, quail) and egg substitutes (oz. eq.); PF_LEGUMES, Legumes computed as protein foods (oz. eq.); PF_MEAT, Beef, veal, pork, lamb, game meat; excludes organ meats and cured meat (oz. eq.); PF_MPS_TOTAL, Total meat, poultry, seafood, organ meats, and cured meat (oz. eq.); PF_NUTSDS, Peanuts, tree nuts, and seeds, excludes coconut (oz. eq.); PF_ORGAN, Organ meat from beef, veal, pork, lamb, game, and poultry (oz. eq.); PF_POULT, Chicken, turkey, Cornish hens, and game birds; excludes organ meats and cured meat (oz. eq.); PF_SEAFD_HI, Seafood (finfish, shellfish and other seafood) high in n‐3 fatty acids (oz. eq.); PF_SEAFD_LOW, Seafood (finfish, shellfish and other seafood) low in n‐3 fatty acids (oz. eq.); PF_SOY, Soy products, excluding calcium fortified soy milk and immature soybeans (oz. eq.); PF_TOTAL, Total meat, poultry, seafood, organ meats, cured meat, eggs, soy, and nuts and seeds; excludes legumes (oz. eq.); *p*‐FDR, false discovery rate corrected *p* value; PhGA, phenylglyoxylic acid; SOLID_FATS, Solid fats (grams); V_DRKGR, Dark green vegetables (cup eq.); V_LEGUMES, Legumes computed as vegetables (cup eq.); V_OTHER, Other vegetables not in the vegetable components listed above (cup eq.); V_REDOR_OTHER, Other red and orange vegetables, excluding tomatoes and tomato products (cup eq.); V_REDOR_TOMATO, Tomatoes and tomato products (cup eq.); V_REDOR_TOTAL, Total red and orange vegetables (tomatoes + other red and orange) (cup eq.); V_STARCHY_OTHER, Other starchy vegetables, excluding white potatoes (cup eq.); V_STARCHY_POTATO, White potatoes (cup eq.); V_STARCHY_TOTAL, Total starchy vegetables (white potatoes + other starchy) (cup eq.); V_TOTAL, Total dark green, red and orange, starchy, and other vegetables; excludes legumes (cup eq.).

Regarding dietary indexes, individuals with higher DII had significantly lower VOCM concentrations, while those with higher healthy dietary indexes, including MEDI, HEI‐2020, AHEI‐2010, and DASHI, had significantly lower VOCM concentrations (Tables S6–S10). However, in the regression analysis, the association directions of VOCMs with higher specific dietary indexes were inconsistent, and BzMA and 2HPMA showed opposite directions compared to other VOCMs (Figure 1). For food groups and nutrients, 2ATCA, BzMA, and 1PMA mostly showed positive correlations, while other VOCMs were basically negative. For example, we observed that several VOCMs exhibited negative associations with fruit, dark green vegetables, total whole and refined grains, dietary fiber, lutein, vitamin K, and copper. We also observed significant positive correlations between VOCMs and dietary fatty acids, particularly saturated fatty acids, SFA 4:0 (Butanoic) and SFA 18:0 (Octadecanoic).

### 3.3 Association of VOCMs With Multi‐Dimensional BA

**FIGURE 2:**
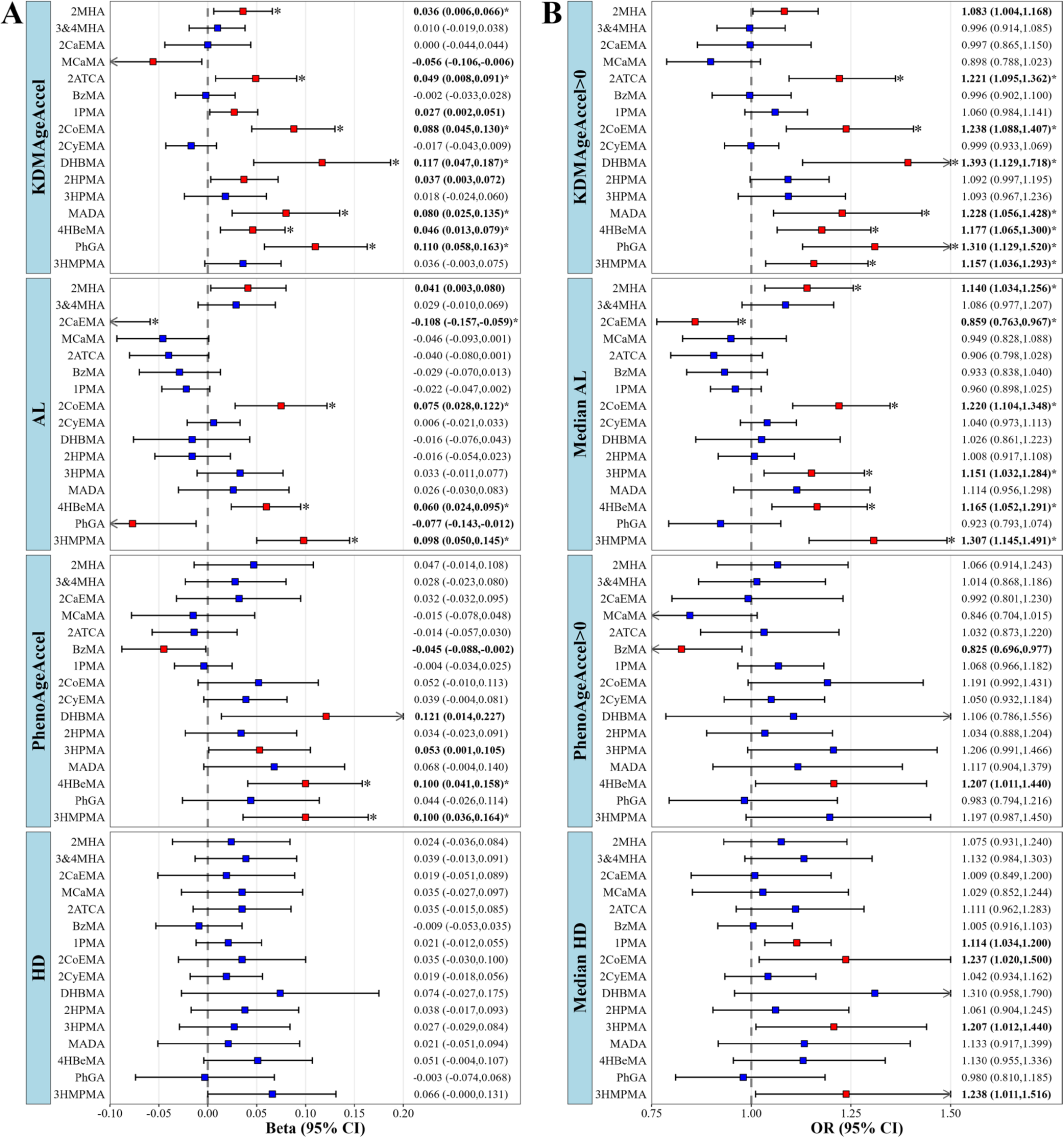
Association of VOCMs with continuous and binary BA in weighted generalized linear models. (A) Continuous BA: KDMAgeAccel, AL, PhenoAgeAccel, and HD; (B) binary BA: KDMAgeAccel > 0, median AL, PhenoAgeAccel > 0, and median HD. Survey‐weighted multivariable linear and logistics regression models were used to assess associations of Ln‐transformed VOCM concentrations with *Z*‐score transformed continuous BA and binary BA, adjusted for age, sex, BMI, race, marital status, education level, PIR, smoking status, drinking status, physical activity (MET‐minutes/week), total energy intake (kcal), diabetes, hypertension, hyperlipidemia, and urinary creatinine. FDR correction was applied to all *p* values across 16 VOCMs separately for each BA indicator. Red effect values and bold text indicate *p* < 0.05, asterisks indicate *p*‐FDR < 0.05. 1PMA, *N*‐acetyl‐S‐(*n*‐propyl)‐L‐cysteine; 2ATCA, 2‐aminothiazoline‐4‐carboxylic acid; 2CaEMA, *N*‐acetyl‐S‐(2‐carbamoylethyl)‐L‐cysteine; 2CoEMA, *N*‐acetyl‐S‐(2‐carboxyethyl)‐L‐cysteine; 2CyEMA, *N*‐acetyl‐S‐(2‐cyanoethyl)‐L‐cysteine; 2HPMA, *N*‐acetyl‐S‐(2‐hydroxypropyl)‐L‐cysteine; 2MHA, 2‐methylhippuric acid; 3&4MHA, 3‐ and 4‐methylhippuric acid; 3HMPMA, *N*‐acetyl‐S‐(3‐hydroxypropyl‐1‐methyl)‐L‐cysteine; 3HPMA, *N*‐acetyl‐S‐(3‐hydroxypropyl)‐L‐cysteine; 4HBeMA, *N*‐acetyl‐S‐(4‐hydroxy‐2‐butenyl)‐L‐cysteine; 95% CI, 95% confidence interval; AL, allostatic load; BzMA, *N*‐acetyl‐S‐(benzyl)‐L‐cysteine; DHBMA, *N*‐acetyl‐S‐(3,4‐dihydroxybutyl)‐L‐cysteine; HD, homeostatic dysregulation; KDMAgeAccel, Klemera‐Doubal method biological age acceleration; MADA, mandelic acid; MCaMA, *N*‐acetyl‐S‐(*N*‐methylcarbamoyl)‐L‐cysteine; OR, odds ratio; *p*‐FDR, false discovery rate corrected *p* value; PhenoAgeAccel, phenotypic age acceleration; PhGA, phenylglyoxylic acid.

We observed that 8 VOCMs (2MHA, 2ATCA, 2CoEMA, DHBMA, MADA, 4HBeMA, PhGA, and 3HMPMA) exhibited significant positive associations with KDMAgeAccel (Figure 2; Table S11). Among them, 6 VOCMs had robust correlations with KDMAgeAccel, with DHBMA showing the strongest correlation (continuous KDMAgeAccel: β = 0.117 (95% CI: 0.047, 0.187); binary KDMAgeAccel: OR = 1.393 (95% CI: 1.129, 1.718)). There were 6 VOCMs (2MHA, 2CaEMA, 2CoEMA, 3HPMA, 4HBeMA, and 3HMPMA) that exhibited positive correlations with AL, while 4 VOCMs showed robust correlations, with 3HMPMA showing the strongest correlation (continuous AL: β = 0.098 (95% CI: 0.050, 0.145); binary AL: OR = 1.307 (95% CI: 1.145, 1.491)). For PhenoAgeAccel and HD, only 2 VOCMs (4HBeMA and 3HMPMA) were significantly positively correlated with PhenoAgeAccel, with no significant associations found for HD (Figure 2, Table S12).

Taken together, 2CoEMA and 4HBeMA showed significant positive associations with both continuous and binary KDMAgeAccel and AL, and 4HBeMA was additionally associated with PhenoAgeAccel; beta effect coefficients of 4HBeMA were 0.046 (95% CI: 0.013, 0.079) for KDMAgeAccel, 0.060 (95% CI: 0.024, 0.095) for AL, and 0.100 (95% CI: 0.041, 0.158) for PhenoAgeAccel. These data suggest that 2CoEMA and 4HBeMA may play major roles in the association between VOCMs and BA. Moreover, we examined the correlation of VOCMs with original biological age, KDM‐BA and PA, and observed strong positive correlations, with DHBMA showing the strongest associations (KDM‐BA: β = 5.944 (95% CI: 4.427, 7.460); PA: β = 7.450 (95% CI: 5.227, 9.673)) (Table S13).

The weighted qgcomp models revealed that VOCM mixture had significant effects on KDMAgeAccel (β = 0.081, 95% CI: 0.015, 0.148) and risk of KDMAgeAccel > 0 (OR = 1.235, 95% CI: 1.097, 1.391). Although we observed no significant associations between individual VOCMs and HD, the VOCM mixture was significantly associated with HD (β = 0.104, 95% CI: 0.014, 0.195) and risk of median HD (OR = 1.199, 95% CI: 1.029, 1.398) (Table 2).

**TABLE 2:** Association between VOCMs mixture and biological aging in weighted qgcomp models.

	β (95% CI)	*p*		OR (95% CI)	*p*
KDMAgeAccel	**0.081 (0.015, 0.148)**	**0.017**	KDMAgeAccel > 0	**1.235 (1.097, 1.391)**	**0.001**
PhenoAgeAccel	0.074 (−0.008, 0.156)	0.077	PhenoAgeAccel > 0	1.113 (0.957, 1.294)	0.165
AL	−0.014 (−0.078, 0.050)	0.671	Median AL	0.979 (0.868, 1.103)	0.725
HD	**0.104 (0.014, 0.195)**	**0.024**	Median HD	**1.199 (1.029, 1.398)**	**0.020**

*Note:* In weighted qgcomp models, VOCM concentrations were Ln‐transformed, biological aging indicators were scaled into *Z*‐scores. The covariates included age, sex, BMI, race, marital status, education level, PIR, smoking status, drinking status, physical activity (MET‐minutes/week), total energy intake (kcal), diabetes, hypertension, hyperlipidemia, and urinary creatinine. Bold font indicates effect estimates were statistically significant, *p* < 0.05.

Abbreviations: 95% CI, 95% confidence interval; AL, allostatic load; HD, homeostatic dysregulation; KDMAgeAccel, Klemera‐Doubal method biological age acceleration; OR, odds ratio; PhenoAgeAccel, phenotypic age acceleration.

### 3.4 Interaction of VOCMs and Dietary Indexes on Multi‐Dimensional BA

**FIGURE 3:**
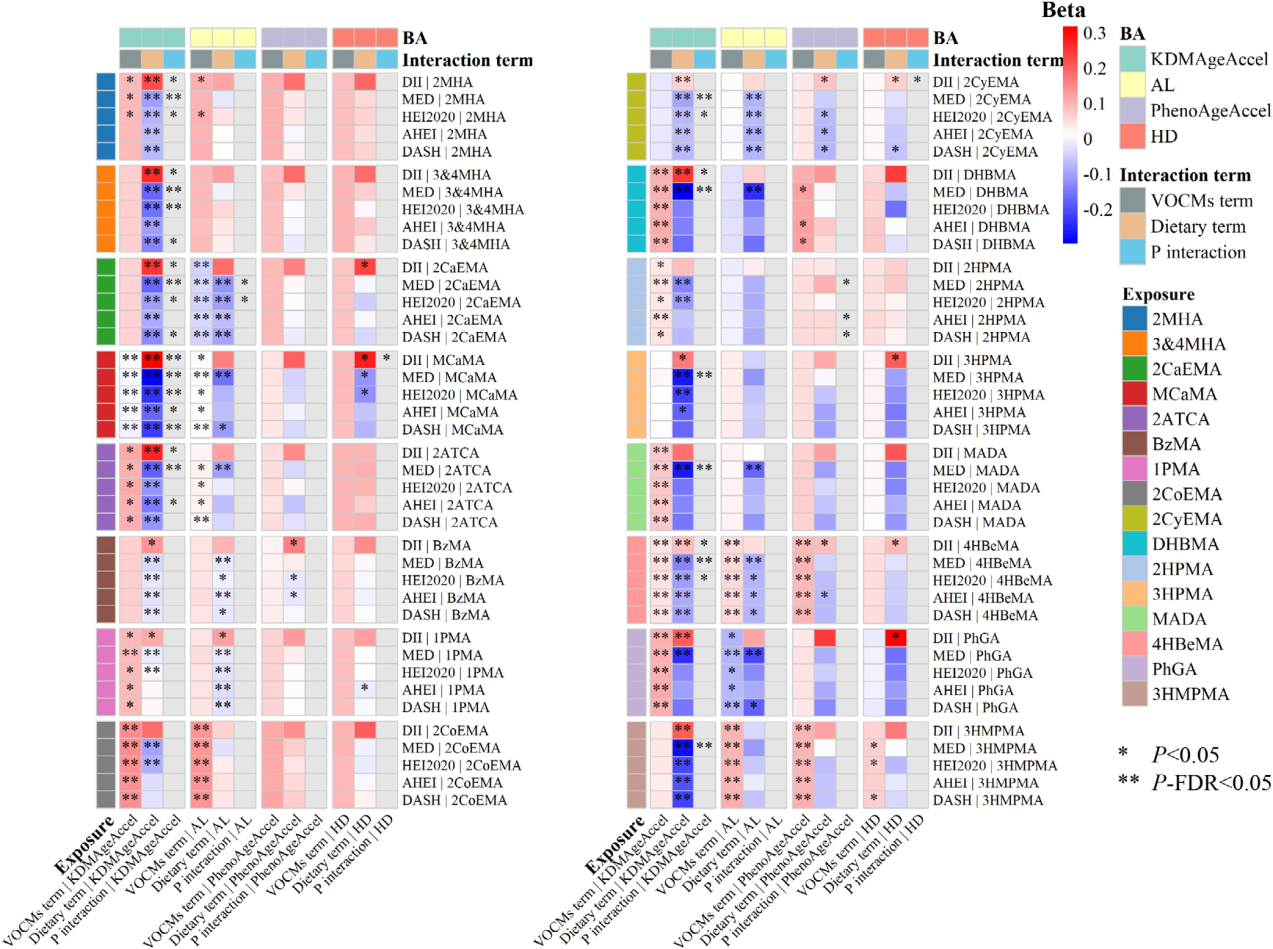
Interaction of VOCMs and dietary indexes on continuous BA in weighted linear regression models. Survey‐weighted multivariable linear regression models were used to assess interaction of Ln‐transformed VOCM concentrations and *Z*‐score transformed dietary indexes on *Z*‐score transformed BA, adjusted for age, sex, BMI, race, marital status, education level, PIR, smoking status, drinking status, physical activity (MET‐minutes/week), total energy intake (kcal), diabetes, hypertension, hyperlipidemia, and urinary creatinine. The figure shows the effect values and *p* values of VOC items and dietary items, as well as the *p* value of the interaction term in the interaction model. FDR correction was applied to all *p* values across 16 VOCMs separately for each combination of a BA indicator and a dietary index. **p‐FDR < 0.05, *p < 0.05. 1PMA, *N*‐acetyl‐*S*‐(*n*‐propyl)‐L‐cysteine; 2ATCA, 2‐aminothiazoline‐4‐carboxylic acid; 2CaEMA, *N*‐acetyl‐*S*‐(2‐carbamoylethyl)‐L‐cysteine; 2CoEMA, *N*‐acetyl‐*S*‐(2‐carboxyethyl)‐L‐cysteine; 2CyEMA, *N*‐acetyl‐*S*‐(2‐cyanoethyl)‐L‐cysteine; 2HPMA, *N*‐acetyl‐*S*‐(2‐hydroxypropyl)‐L‐cysteine; 2MHA, 2‐methylhippuric acid; 3&4MHA, 3‐ and 4‐methylhippuric acid; 3HMPMA, *N*‐acetyl‐*S*‐(3‐hydroxypropyl‐1‐methyl)‐L‐cysteine; 3HPMA, *N*‐acetyl‐*S*‐(3‐hydroxypropyl)‐L‐cysteine; 4HBeMA, *N*‐acetyl‐*S*‐(4‐hydroxy‐2‐butenyl)‐L‐cysteine; 95% CI, 95% confidence interval; AHEI‐2010, Alternative Healthy Eating Index‐2010; AL, allostatic load; BzMA, *N*‐acetyl‐*S*‐(benzyl)‐L‐cysteine; DASHI, Dietary Approaches to Stop Hypertension Index; DHBMA, *N*‐acetyl‐*S*‐(3,4‐dihydroxybutyl)‐L‐cysteine; DII, Dietary Inflammatory Index; HEI‐2020, Healthy Eating Index‐2020; KDMAgeAccel, Klemera‐Doubal method biological age acceleration; MADA, mandelic acid; MCaMA, *N*‐acetyl‐*S*‐(*N*‐methylcarbamoyl)‐L‐cysteine; MEDI, Mediterranean Diet Index; PhGA, phenylglyoxylic acid.

We included product terms of VOCMs and dietary indexes in linear regression models to explore their interaction on continuous BA indicators (Figure 3, Tables S14 and S15). We observed significant interactions between 11 VOCMs and at least one dietary index on KDMAgeAccel (*p* for interaction < 0.05, Figure 3; Table S14). In these models with significant interaction, the effect size of DII was significantly positive, while the effect size of other indexes (MEDI, HEI‐2020, AHEI‐2010, and DASHI) was significantly negative. For example, in the model for 4HBeMA, which was associated with the largest number of BA indicators among VOCMs, the VOCMs term showed significant positive correlations as expected, the DII term showed a significant positive effect (β = 0.086 (95% CI: 0.031, 0.141)), and other dietary indexes showed significant negative correlations (MEDI: β = −0.135 (95% CI: −0.187, −0.083); HEI‐2020: β = −0.106 (95% CI: −0.167, −0.045); AHEI‐2010: β = −0.095 (95% CI: −0.155, −0.035); DASHI: β = −0.100 (95% CI: −0.166, −0.034)), and significant interactions were found for DII and MEDI. These results suggest that diet modifies the association of VOCMs with BA, and a healthy diet may offer protection against the effects of VOCMs on BA. For AL, PhenoAgeAccel, and HD, there were few significant interactions between VOCMs and dietary indexes, and the dietary index term also had similar effect size direction to those in the analysis of KDMAgeAccel (Figure 3; Table S15).

## 4 Discussion

In a nationally representative U.S. population, dietary factors significantly drive population‐level VOCM concentrations. KDMAgeAccel was the most sensitive indicator of the positive association between VOCMs and multi‐dimensional BA. Furthermore, dietary indexes potentially modified the relationship between VOCMs and KDMAgeAccel, highlighting potential nutritional strategies to mitigate VOCMs‐related aging.

### 4.1 Dietary Factors Affecting VOCM Concentrations

Our results suggested that healthy dietary habits may have a protective effect on urinary VOCM concentrations. Specifically, higher intake of dark‐colored vegetables, fruits, grains, dietary fiber, lutein, vitamin K, and copper was negatively associated with several urinary VOCMs. Consistent with our findings, Bosch et al. reported that a vegetarian diet significantly altered fecal VOC patterns, whereas regular diets showed no notable effects (Bosch et al. 2019). Similarly, Baranska et al. (2013) demonstrated that a gluten‐free diet induced reversible changes in the profile of VOCs in breath. However, the mechanisms by which these foods and nutrients influence VOCM levels in humans remain unclear and require further investigation. For example, lutein, abundant in dark‐colored vegetables and fruits, possesses antioxidant properties, which may contribute to reduced formation or faster clearance of harmful metabolites (Pap et al. 2021). Additionally, our finding that oil intake was associated with increased urinary 2CaEMA (parent compound: Acrylamide) levels underscores the potential exposure risks linked to certain cooking practices. Acrylamide is a toxic compound widely present in heat‐processed foods, particularly those subjected to frying, grilling, and baking (Koszucka and Nowak 2019). This finding highlights the role of oil‐based cooking methods, such as frying, in acrylamide exposure.

In dietary patterns analysis, our research first demonstrated that individuals with higher DII or lower healthy dietary indexes, including MEDI, HEI‐2020, AHEI‐2010, and DASHI, exhibit elevated urinary concentrations of several VOCMs. High DII pattern reflects diets rich in calories, sugar, and fat (Shivappa et al. 2014). Supporting this, Kistler et al. (2016) observed elevated acrolein in the exhaled breath of obese mice induced by high‐fat diet. Beyond exogenous exposures like air pollution and smoking, acrolein can also be endogenously produced through lipid peroxidation under oxidative stress (Stevens and Maier 2008), a process exacerbated by high‐fat diets. However, we did not observe significant associations between fatty acid intake and metabolites of acrolein. Furthermore, metabolic competition is a critical factor to consider regarding urinary VOCMs. Because VOC biotransformation relies heavily on hepatic cytochrome P450 enzymes, diets rich in competing compounds—such as caffeine, which places a continuous demand on CYP1A2 (Esteves et al. 2023)—can theoretically induce competitive inhibition. Consequently, specific dietary patterns may alter urinary VOCM profiles by modifying internal toxicokinetics rather than simply reflecting external exposures. To further understand the protective effects of healthier diets on urinary VOCMs, future studies should investigate the molecular and biochemical mechanisms underlying these associations while comprehensively accounting for potential confounders.

Beyond these mechanistic considerations, it is crucial to contextualize the public health relevance of our findings. Importantly, dietary‐driven variations in VOCM concentrations represent chronic, low‐dose exposures. These levels are markedly lower than those in occupational settings, where workers (e.g., coke oven operators) frequently exhibit urinary VOCMs 2 to 10 times higher (Frigerio et al. 2020). Nevertheless, given the ubiquitous nature of daily food consumption, this low‐dose pathway remains a critical contributor to cumulative lifelong exposure.

### 4.2 Association of VOCMs With Multi‐Dimensional BA

Previous studies on VOCs and aging focused on exhalation or feces, neglecting urine‐derived VOCMs. For example, the frequency distributions of VOCs in exhaled breath varied significantly among centenarians, seniors, and young subjects, and dimethyl trisulfide and 1H‐indole in feces were associated with aging (Conte et al. 2020; Mazzatenta et al. 2015). Due to the short half‐life and volatile nature of VOCs, VOCMs exhibit relatively delayed clearance and are easier to measure. We thus utilized urinary VOCMs to estimate human exposure levels and explore their associations with BA.

In our study, we assessed BA using four biomarkers: KDMAgeAccel, PhenoAgeAccel, HD, and AL. Our findings revealed that the associations between VOCMs and KDMAgeAccel were the most significant and comprehensive, suggesting that KDMAgeAccel may be more sensitive in capturing VOCM effects on BA. This difference may be related to the algorithm and biological implications of KDMAgeAccel. The process of BA involves complex, system‐wide functional declines (Fabbri et al. 2015). KDMAgeAccel incorporates clinical biomarkers from five systems based on chronological age regression. Consequently, this cross‐system integration may reflect the systemic aging effects of VOCMs. Moreover, KDMAgeAccel's exclusion of complete blood count, which is susceptible to short‐term health fluctuations like infections, may make it a more reliable and robust biomarker for reflecting BA (Yildiz Balci et al. 2020).

In contrast, PhenoAgeAccel and HD do not consider the cardiovascular system, despite cardiovascular diseases being an important hallmark of aging, which may explain the fewer associations of VOCMs with PhenoAgeAccel or HD. Furthermore, PhenoAgeAccel is heavily weighted toward inflammatory biomarkers. The lack of broad associations with PhenoAgeAccel suggests that while some VOCs induce inflammation, the primary mechanisms driving VOC‐related aging in the general population may involve non‐inflammatory pathways, such as direct DNA adduction, genomic instability, and widespread macromolecular damage (López‐Otín et al. 2023), which are better captured by the multi‐system functional integration of KDMAgeAccel. Moreover, due to data missingness, the limited sample sizes for PhenoAgeAccel and HD may have influenced the analytical results, partially explaining the fewer associations. Hastings et al. suggested that AL is often constrained by the specific distribution of the studied population, with risk definitions relying on biomarker quantile distributions within the analytical sample (Hastings et al. 2022). This constraint could lead to underestimation or bias in effect sizes, particularly in healthy or heterogeneous populations. As an indicator based on multi‐system integration, KDMAgeAccel may be a potentially critical biomarker for characterizing the impact of VOCMs on multi‐dimensional BA.

Research examining the relationship between exposure to VOCs and BA remains notably sparse. A panel study of 73 healthy older adults showed personal exposure to 1,4‐dichlorobenzene significantly accelerated DNA methylation PhenoAge (DNAmPhenoAge) (W. Shi et al. 2022). 1,4‐Dichlorobenzene, widely used as a pesticide, has been shown to induce DNA damage in livers and spleens of mice (Canonero et al. 1997), which plays a causal role in aging (Yousefzadeh et al. 2021). Aligning with these findings, we observed that MCaMA (parent compounds: N,N‐dimethylformamide and Methyl isocyanate) is positively associated with increased KDM‐BA and PA. Long‐term exposure to N,N‐dimethylformamide (a precursor to MCaMA) is highly associated with mitochondrial DNA (mtDNA) alterations (Shieh et al. 2007), which have been elucidated to accelerate aging. Although the latest research has reported a positive association between VOCs and a type of BA (PhenoAgeAccel), it did not explore the heterogeneity of the association between VOCs and different BAs, nor did it investigate diet‐related mitigation strategies (Hong et al. 2025; Shi et al. 2025).

Our research indicated that 2CoEMA (parent compound: acrolein) and 4HBeMA (parent compound: 1,3‐Butadiene) exhibit a significant positive association with KDMAgeAccel and AL. The biological plausibility of these associations is well‐supported by extensive literature. Mechanistically, acrolein accelerates aging through multiple pathways that directly correspond to established hallmarks of aging. First, it induces oxidative stress by depleting glutathione, causing mitochondrial dysfunction and macromolecular damage (Igarashi et al. 2018). Second, it activates pro‐inflammatory signaling cascades like NF‐κB, promoting chronic low‐grade inflammation, a hallmark of aging (Newaz and Yousefipour 2013). Third, acrolein interferes with genomic stability by forming DNA adducts and suppressing the expression of DNA repair enzymes such as WRN helicase, thus facilitating the accumulation of somatic mutations. Similarly, 1,3‐butadiene exerts its aging‐accelerating effects through well‐characterized mechanisms. Its reactive metabolites, particularly diepoxybutane, form DNA–DNA cross‐links that lead to chromosomal aberrations and genomic instability (Goggin et al. 2009). Additionally, 1,3‐butadiene disrupts epigenetic homeostasis through global DNA hypomethylation and altered histone modifications, processes that are central to aging‐related changes in gene expression patterns (Galow and Peleg 2022). These molecular mechanisms provide a robust biological foundation for our observed associations between 2CoEMA/4HBeMA and accelerated BA.

However, a critical toxicological caveat must be considered. VOCs are predominantly detoxified by hepatic enzymes and their metabolites are actively excreted via the kidneys. Consequently, chronic VOC exposure may induce localized hepatotoxicity or direct renal strain. Because KDMAgeAccel incorporates clinical markers of liver and kidney function (e.g., serum albumin, blood urea nitrogen, serum creatinine), direct toxicological insults to these specific clearance organs could disproportionately drive the KDMAgeAccel score upward. Thus, the observed aging acceleration may partially reflect targeted organ‐system strain rather than purely generalized systemic senescence.

### 4.3 Interaction of VOCMs and Dietary Indexes on Multi‐Dimensional BA

We also observed that dietary patterns modify the associations between VOCMs and KDMAgeAccel. Healthier diets (lower DII; higher MEDI, HEI‐2020, AHEI‐2010, and DASHI) may protect against VOCM effects on BA. Limited evidence suggests that healthy dietary patterns reduce VOCM‐related accelerated aging. For example, low DII and high MEDI dietary patterns are typically polyphenol‐rich (Shivappa et al. 2014). Studies have demonstrated that dietary polyphenols can mitigate acrolein toxicity by reducing endogenous acrolein production through inhibition of lipid peroxidation and by directly scavenging acrolein (Y. Zhou et al. 2024). Additionally, polyphenols can suppress acrolein‐induced inflammatory responses by inhibiting NF‐κB signaling pathways (Nani et al. 2021) and alleviate acrolein‐induced cellular senescence by regulating the p53 signaling pathway (Kim et al. 2018). These findings suggest that polyphenols may reduce hallmarks of aging related to acrolein. Unfortunately, our study did not include polyphenols when investigating how nutrients influence urinary VOCM concentrations. However, we found that the intake of fruits and dark vegetables—polyphenol‐rich foods—was associated with decreased levels of 3HPMA, an acrolein metabolite. This supports the hypothesis that polyphenols protect against VOCM‐related accelerated aging. Furthermore, vitamin E, green tea polyphenols, and N‐acetylcysteine, which are commonly recommended as part of healthier dietary patterns, have been shown to reduce acrylonitrile‐induced oxidative DNA damage by enhancing the activity of apurinic/apyrimidinic endonuclease 2 (APEX2), a DNA repair enzyme (Pu et al. 2016). Our findings also indicate that the intake of vitamin E‐rich foods, such as fruits, dark vegetables, and whole grains, can lower urinary levels of acrylonitrile metabolites. Therefore, we propose that healthy dietary patterns, characterized by low DII and high MEDI, HEI‐2020, AHEI‐2010, and DASHI, represent a novel nutritional strategy to modify VOCM‐related BA.

### 4.4 Strengths and Limitations

Our study has several strengths. First, we identified comprehensive dietary exposure‐related inequalities in VOC exposure, providing a basis for targeted interventions. Second, we identified KDMAgeAccel as the most sensitive biomarker linking VOCMs to multi‐dimensional BA, offering a tool to understand VOCM aging impacts. Third, by examining the interaction between dietary patterns and VOCMs on BA, this study is among the first to highlight the potential of nutritional strategies in mitigating VOCM‐related aging acceleration. However, the study also has limitations. First, its cross‐sectional design precludes causal inferences between VOCMs, dietary patterns, and BA. Specifically, a critical temporal discrepancy exists: short half‐life urinary VOCMs and 1‐to‐2 days of dietary recall provide only acute exposure snapshots, which may not perfectly capture the lifelong cumulative toxicological burden or long‐term nutritional habits driving progressive biological aging. Furthermore, we cannot rule out reverse causality related to physiological decline. Because VOC metabolites are primarily eliminated via the kidneys, individuals with advanced BA and concomitant declines in renal function may exhibit impaired clearance and subsequent urinary accumulation of these metabolites. Longitudinal studies are needed to establish temporal relationships and clarify these complex, age‐related toxicokinetic shifts. Second, urinary VOCM levels may not fully represent systemic exposure. Still, urine analysis is advantageous for its stability, broad detection window, and non‐invasiveness, making it suitable for assessing long‐term exposure (Alwis et al. 2012; Hu et al. 2022). Third, VOCM levels were measured at a single time point, which may not adequately capture chronic exposure patterns. However, Qian et al. demonstrated good inter‐day reproducibility of urinary VOCM measurements (Qian et al. 2021), suggesting the reliability of our results. Fourth, although certain foods (e.g., grains, dark green vegetables) were associated with lower VOCM levels, they are also primary dietary vectors for aging‐accelerating heavy metals (e.g., cadmium and lead) (Kan et al. 2026; Mei et al. 2025). The omission of these co‐contaminants may confound the net impact of diet on biological aging. Fifth, the lack of genotype data precluded the exploration of potential gene–environment interactions (Wang et al. 2026). Finally, the study's focus on American adults may limit its generalizability to other populations. Future research should further explore the utility of KDMAgeAccel as a key biomarker for the effects of VOCMs on BA across diverse populations and in longitudinal studies to validate its broader applicability.

## 5 Conclusions

In conclusion, we identified dietary factors as drivers of population‐level inequalities in VOC exposure in a nationally representative U.S. population. KDMAgeAccel was the most sensitive marker linking VOCMs to multi‐dimensional BA. Furthermore, dietary patterns modified the relationship between VOCMs and KDMAgeAccel. Future longitudinal research across diverse populations is needed to validate our findings and elucidate the underlying mechanisms. These findings highlight the need for targeted strategies, including VOC exposure reduction and dietary interventions, to promote healthier, more equitable aging outcomes.

## Author Contributions

Weitao Su: Funding acquisition, Investigation, Methodology, Visualization, Formal analysis, Data curation, Software, Writing original draft, Writing – review and editing. Yaoyu Hu: Investigation, Methodology, Visualization, Formal analysis, Data curation, Software, Writing original draft, Writing – review and editing. Jindong Zhao: Investigation, Methodology, Visualization, Formal analysis, Data curation, Software, Writing original draft, Writing – review and editing. Ming Yang: Investigation, Methodology, Data curation, Writing – review and editing. Jingtao Wu: Investigation, Methodology, Data curation, Writing – review and editing. Danfeng Wen: Investigation, Methodology, Data curation, Writing – review and editing. Zhiqi Lin: Investigation, Methodology, Visualization, Software, Writing – review and editing. Jiaxin Zhao: Investigation, Methodology, Visualization, Software, Writing – review and editing. Yanbing Li: Investigation, Methodology, Visualization, Software, Writing – review and editing. Jiufeng Li: Conceptualization, Funding acquisition, Project administration, Resources, Supervision, Writing – review and editing. Ang Li: Conceptualization, Funding acquisition, Project administration, Resources, Supervision, Writing – review and editing.

## Funding

This work was supported by National Natural Science Foundation of China (82404365), Noncommunicable Chronic Diseases–National Science and Technology Major Project (2023ZD0513200), Shanghai 3‐year Public Health Action Plan (GWVI‐11.1‐39), Shanghai Magnolia talent plan Pujiang project (24PJA020), Natural Science Foundation of Shanghai (25ZR1401069), Institutional Fund of The Second Affiliated Hospital of Fujian Medical University (BS202503), Young Elite Scientists Sponsorship Program of the Beijing High Innovation Plan (20250761), Joint Funds for the Innovation of Science and Technology, Fujian Province (2024Y9367), and Natural Science Foundation of Fujian Province (2025J01820).

## Ethics Statement

This study was approved by the NCHS Ethics Review Board.

## Consent

Written informed consent was obtained from all study participants.

## Conflicts of Interest

The authors declare no conflicts of interest.

## Supporting information


**Data S1:** Supplementary methods. Calculations of KDM‐BA, PA, HD, and AL.
**Figure S1:** Flowchart of selection of study participants.
**Figure S2:** Spearman correlation coefficients for VOCMs.
**Table S1:** Summary of all VOCMs detected in NHANES cycles.
**Table S2:** Variables used in calculating KDM‐BA, PA, HD and AL.
**Table S3:** Components of several healthy dietary indexes.
**Table S4:** Variance inflation factors (VIFs) of covariates in the survey‐weighted multivariable linear regression models for KDMAgeAccel.
**Table S5:** Detection rates and distribution of included VOCMs cross cycles.
**Table S6:** BA indicators and VOCM concentrations of subjects by median DII.
**Table S7:** BA indicators and VOCM concentrations of subjects by moderate to high adherence to MEDI.
**Table S8:** BA indicators and VOCM concentrations of subjects by median HEI‐2020.
**Table S9:** BA indicators and VOCM concentrations of subjects by median AHEI‐2010.
**Table S10:** BA indicators and VOCM concentrations of subjects by median DASHI.
**Table S11:** Association of VOCMs with continuous and binary KDMAgeAccel and AL in weighted generalized linear models.
**Table S12:** Association of VOCMs with continuous and binary PhenoAgeAccel and HD in weighted generalized linear models.
**Table S13:** Association of VOCMs with KDM‐BA and PA in weighted linear regression models.
**Table S14:** Interaction of VOCMs and dietary indexes on KDMAgeAccel and AL in weighted linear regression models.
**Table S15:** Interaction of VOCMs and dietary indexes on PhenoAgeAccel and HD in weighted linear regression models.

## Data Availability

All data are available in the NHANES official website (https://wwwn.cdc.gov/nchs/nhanes/).
