# Supplementary material for: Dietary Factors Drive Volatile Organic Compound Exposure and Modulate Its Impact on Multi‐Dimensional Biological Aging
Source: Food Sci Nutr. 2026 Apr 13;14(4):e71753. doi: 10.1002/fsn3.71753 (PMC13071531; doi:10.1002/fsn3.71753)
Supplement: Supplementary file 1 — Data S1: Supplementary methods. Calculations of KDM‐BA, PA, HD, and AL. Figure S1: Flowchart of selection of study participants. Figure S2: Spearman correlation coefficients for VOCMs. Table S1: Summary of all VOCMs detected in NHANES cycles. Table S2: Variables used in calculating KDM‐BA, PA, HD and AL. Table S3: Components of several healthy dietary indexes. Table S4: Variance inflation factors (VIFs) of covariates in the survey‐weighted multivariable linear regression models for KDMAgeAccel. Table S5: Detection rates and distribution of included VOCMs cross cycles. Table S6: BA indicators and VOCM concentrations of subjects by median DII. Table S7: BA indicators and VOCM concentrations of subjects by moderate to high adherence to MEDI. Table S8: BA indicators and VOCM concentrations of subjects by median HEI‐2020. Table S9: BA indicators and VOCM concentrations of subjects by median AHEI‐2010. Table S10: BA indicators and VOCM concentrations of subjects by median DASHI. Table S11: Association of VOCMs with continuous and binary KDMAgeAccel and AL in weighted generalized linear models. Table S12: Association of VOCMs with continuous and binary PhenoAgeAccel and HD in weighted generalized linear models. Table S13: Association of VOCMs with KDM‐BA and PA in weighted linear regression models. Table S14: Interaction of VOCMs and dietary indexes on KDMAgeAccel and AL in weighted linear regression models. Table S15: Interaction of VOCMs and dietary indexes on PhenoAgeAccel and HD in weighted linear regression models. [file FSN3-14-e71753-s001.docx]

**Supporting Information**

**Dietary Factors Drive Volatile Organic Compound Exposure and Modulate Its Impact on Multi-Dimensional Biological Aging**

**Diet Modifies VOCs' Impact on Aging**

Weitao Su^a,#^, Yaoyu Hu^b,c,#^, Jindong Zhao^d,e^, Ming Yang^b,c^, Jingtao Wu^b,c^, Danfeng Wen^f,g^, Zhiqi Lin^f,g^, Jiaxin Zhao^c^, Yanbing Li^c^, Jiufeng Li^f,g**^, Ang Li^d,e*^

a Central Laboratory, The Second Affiliated Hospital of Fujian Medical University, Quanzhou, Fujian 362000, China.

b Center for Rare Diseases, State Key Laboratory of Complex, Severe, and Rare Diseases, Peking Union Medical College Hospital, Chinese Academy of Medical Sciences, Beijing, 100730, China.

c Department of Epidemiology and Biostatistics, Institute of Basic Medical Sciences, Chinese Academy of Medical Sciences, School of Basic Medicine Peking Union Medical College, Beijing 100005, China

d Department of Epidemiology and Statistics, School of Public Health, Hebei Medical University, Shijiazhuang, 050017, China.

e Hebei Key Laboratory of Environment and Human Health, Hebei Province, Shijiazhuang 050017, China.

f Key Laboratory of Health Technology Assessment, National Health Commission of the People’s Republic of China, Fudan University, Shanghai 200032, China.

g Key Laboratory of Public Health Safety, Ministry of Education, School of Public Health, Fudan University, Shanghai 200032, China.

^#^ These authors contributed equally to this paper.

^*^Corresponding Author:

**Jiufeng Li**, Key Laboratory of Health Technology Assessment, National Health Commission of the People’s Republic of China, Fudan University, Shanghai 200032, China; Key Laboratory of Public Health Safety, Ministry of Education, School of Public Health, Fudan University, Shanghai 200032, China;

Email: lijiufeng@fudan.edu.cn.

**Ang Li,** Department of Epidemiology and Statistics, School of Public Health, Hebei Medical University, Shijiazhuang, 050017, China; Hebei Key Laboratory of Environment and Human Health, Hebei Province, Shijiazhuang, 050017, China.

Email: [pumcleon@ibms.pumc.edu.cn](mailto:pumcleon@ibms.pumc.edu.cn).

**Supplementary Material Content**

[**Supplementary methods. Calculations of KDM-BA, PA, HD, and AL** 4](#_Toc223996982)

[**Figure S1. Flowchart of selection of study participants.** 8](#_Toc223996983)

[**Figure S2. Spearman correlation coefficients for VOCMs.** 9](#_Toc223996984)

[**Table S1. Summary of all VOCMs detected in NHANES cycles** 10](#_Toc223996985)

[**Table S2. Variables used in calculating KDM-BA, PA, HD and AL** 12](#_Toc223996986)

[**Table S3. Components of several healthy dietary indexes** 14](#_Toc223996987)

[**Table S4. Variance inflation factors (VIFs) of covariates in the survey-weighted multivariable linear regression models for KDMAgeAccel.** 15](#_Toc223996988)

[**Table S5. Detection rates and distribution of included VOCMs cross cycles** 18](#_Toc223996989)

[**Table S6. BA indicators and VOCM concentrations of subjects by median DII** 20](#_Toc223996990)

[**Table S7. BA indicators and VOCM concentrations of subjects by moderate to high adherence to MEDI** 22](#_Toc223996991)

[**Table S8. BA indicators and VOCM concentrations of subjects by median HEI-2020** 24](#_Toc223996992)

[**Table S9. BA indicators and VOCM concentrations of subjects by median AHEI-2010** 26](#_Toc223996993)

[**Table S10. BA indicators and VOCM concentrations of subjects by median DASHI** 28](#_Toc223996994)

[**Table S11. Association of VOCMs with continuous and binary KDMAgeAccel and AL in weighted generalized linear models** 30](#_Toc223996995)

[**Table S12. Association of VOCMs with continuous and binary PhenoAgeAccel and HD in weighted generalized linear models** 32](#_Toc223996996)

[**Table S13. Association of VOCMs with KDM-BA and PA in weighted linear regression models** 34](#_Toc223996997)

[**Table S14. Interaction of VOCMs and dietary indexes on KDMAgeAccel and AL in weighted linear regression models** 36](#_Toc223996998)

[**Table S15. Interaction of VOCMs and dietary indexes on PhenoAgeAccel and HD in weighted linear regression models** 41](#_Toc223996999)

**Supplementary methods. Calculations of KDM-BA, PA, HD, and AL**

The KDM-BA algorithm was developed through a set of regressions of individual biomarkers on chronological age in a reference population(Klemera & Doubal, 2006). The formula derives from n regression lines which regress chronological age on n biomarkers, and it is shown below:

$${BA}_{EC}=\frac{\sum_{i=1}^{n} \left( x_{i}-q_{i} \right)\frac{k_{i}}{s_{i}^{2}}+\frac{CA}{S_{BA}^{2}}}{\sum_{i=1}^{n} {(\frac{k_{i}}{S_{i}})}^{2}+\frac{1}{S_{BA}^{2}}}$$

Where *x* represents the measured value of biomarker *i* for an individual. For each biomarker *i*, the parameters k, q, and s are the regression intercept, slope, and root mean squared error, respectively, derived from a regression analysis of chronological age on biomarker in the reference population. S_BA_ is a scaling factor, equivalent to the square root of the variance in chronological age which is accounted for the set of biomarkers in the reference population. CA is short for chronological age. The reference sample comprised non-pregnant participants aged 30 to 75 from NHANES III.

The PA algorithm was originally formulated using elastic-net Gompertz regression of mortality on 42 biomarkers in the NHANES III, and the final model selected nine biomarkers and chronological age(Levine et al., 2018). A mortality prediction score was calculated based on the linear combination of biomarkers from the fitted model, and then converted into a biological age value. PA refers to the chronological age where a participant’s estimated mortality risk matches the average mortality hazard observed in the NHANES reference population. The formula to calculate PA is shown below:

$$PA=\frac{\ln(- 0.00553\times ln(\left( 1-mortality risk \right))}{0.090165}$$

Where $mortality risk=1-e^{\frac{-1.51714\times e^{xb}}{0.0076927}}$

xb = − 19.907 − 0.0336 × albumin + 0.0095 × creatinine + 0.1953 × glucose + 0.0954 × ln (C-reactive protein (CRP)) − 0.0120 ×lymphocute percentage + 0.0268 × mean cell volume + 0.3306 × red blood cell distribution width + 0.00188 × alkaline phosphatase (ALP) + 0.0554 × white blood cell count + 0.0804 × chronological age.

HD is calculated as the Mahalanobis distance for multiple given biomarkers in relation to a reference population(Cohen et al., 2013).

The Mahalanobis distance formula to calculate HD is shown below:

$$D_{M}(\overset{\to}{x})=\sqrt{\left( \overset{\to}{x}-\overset{\to}{\mu} \right)^{T}S^{-1}(\overset{\to}{x}-\overset{\to}{\mu})}$$

Where *x* represents a multivariate observation, encompassing all biomarker values for an individual, while *μ* is the vector of means for each variable in the reference sample. *S* denotes the variance-covariance matrix for the variables in the reference sample. We defined the reference population as non-pregnant participants aged 20 to 30 years old who had all selected biomarkers falling within the clinically normal range in NHANES III. The HD value indicates the extent to which an individual’s physiology deviates from that of a healthy reference group. The calculations of KDM-BA, PA, and HD were performed separately for men and women using the R package “BioAge”(Kwon & Belsky, 2021).

AL represents the cumulative biological damage due to frequent activation of allostatic responses in stressful situations, which captures characteristics of BA across various life stages(Gruenewald et al., 2012; Seeman, Singer, Rowe, Horwitz, & McEwen, 1997). To comprehensively characterize the physiological state of the human body, we used an improved AL, which includes biomarkers targeting three physiological systems (cardiovascular, metabolic, and immune systems) as well as two additional organs (liver and kidney)(Chadeau-Hyam et al., 2020). Due to the lack of insulin-like growth factor 1 (IGF-1) in NHANES, it was replaced with white blood cells (WBC), a common indicator of inflammation, in the immune system. Biomarkers of cardiovascular system (n = 3) included systolic blood pressure (SBP), diastolic blood pressure (DBP), and pulse rate. Biomarkers of metabolic system (n = 4) included glycated hemoglobin (HbA1c), triglyceride, high-density lipoprotein cholesterol (HDL-c), and low-density lipoprotein cholesterol (LDL-c). Biomarkers of immune system (n = 2) included CRP and WBC. Liver function biomarkers (n = 3) included alanine aminotransferase (ALT), aspartate aminotransferase (AST), and γ-glutamyl transferase (GGT). Kidney function biomarkers (n = 1) included serum creatinine. Quartiles for each of these 13 biomarkers were established for each gender and age group (<50, 50-64, and >64 years old) separately. Biomarkers were dichotomised into “high risk” group, and coded as 1 for the lowest quartile of HDL-c, and the highest quartile of all remaining 12 biomarkers, whereas a score of 0 was allocated to non-high-risk group. If a biomarker value was missing, a null sub-score was assigned. Each of the five system-specific sub-scores is calculated as the sum of binary variables, and then standardized by the number of biomarkers in each system to ensure consistency of system-specific sub-scores in measurement scale. Finally, the overall AL is defined as the sum of all system-specific sub-scores, standardized by the number of systems.

**References**

Chadeau-Hyam, M., Bodinier, B., Vermeulen, R., Karimi, M., Zuber, V., Castagné, R., … Delpierre, C. (2020). Education, biological ageing, all-cause and cause-specific mortality and morbidity: UK biobank cohort study. *EClinicalMedicine*, *29–30*, 100658. https://doi.org/10.1016/j.eclinm.2020.100658

Cohen, A. A., Milot, E., Yong, J., Seplaki, C. L., Fülöp, T., Bandeen-Roche, K., & Fried, L. P. (2013). A novel statistical approach shows evidence for multi-system physiological dysregulation during aging. *Mechanisms of Ageing and Development*, *134*(3–4), 110–117. https://doi.org/10.1016/j.mad.2013.01.004

Gruenewald, T. L., Karlamangla, A. S., Hu, P., Stein-Merkin, S., Crandall, C., Koretz, B., & Seeman, T. E. (2012). History of socioeconomic disadvantage and allostatic load in later life. *Social Science & Medicine (1982)*, *74*(1), 75–83. https://doi.org/10.1016/j.socscimed.2011.09.037

Klemera, P., & Doubal, S. (2006). A new approach to the concept and computation of biological age. *Mechanisms of Ageing and Development*, *127*(3), 240–248. https://doi.org/10.1016/j.mad.2005.10.004

Kwon, D., & Belsky, D. W. (2021). A toolkit for quantification of biological age from blood chemistry and organ function test data: BioAge. *GeroScience*, *43*(6), 2795–2808. https://doi.org/10.1007/s11357-021-00480-5

Levine, M. E., Lu, A. T., Quach, A., Chen, B. H., Assimes, T. L., Bandinelli, S., … Horvath, S. (2018). An epigenetic biomarker of aging for lifespan and healthspan. *Aging*, *10*(4), 573–591. https://doi.org/10.18632/aging.101414

Seeman, T. E., Singer, B. H., Rowe, J. W., Horwitz, R. I., & McEwen, B. S. (1997). Price of adaptation—Allostatic load and its health consequences. MacArthur studies of successful aging. *Archives of Internal Medicine*, *157*(19), 2259–2268.


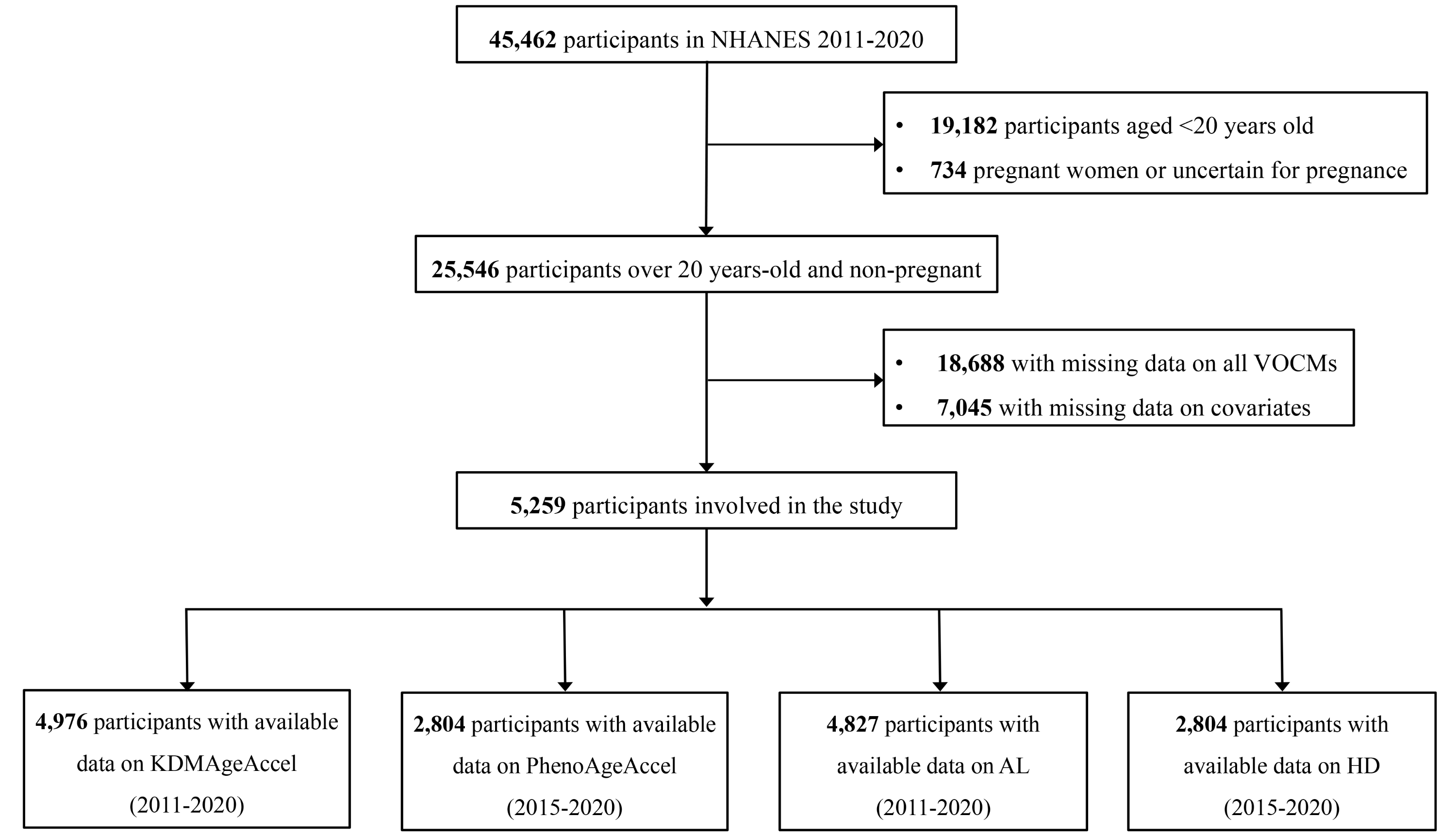


**Figure S1. Flowchart of selection of study participants.**


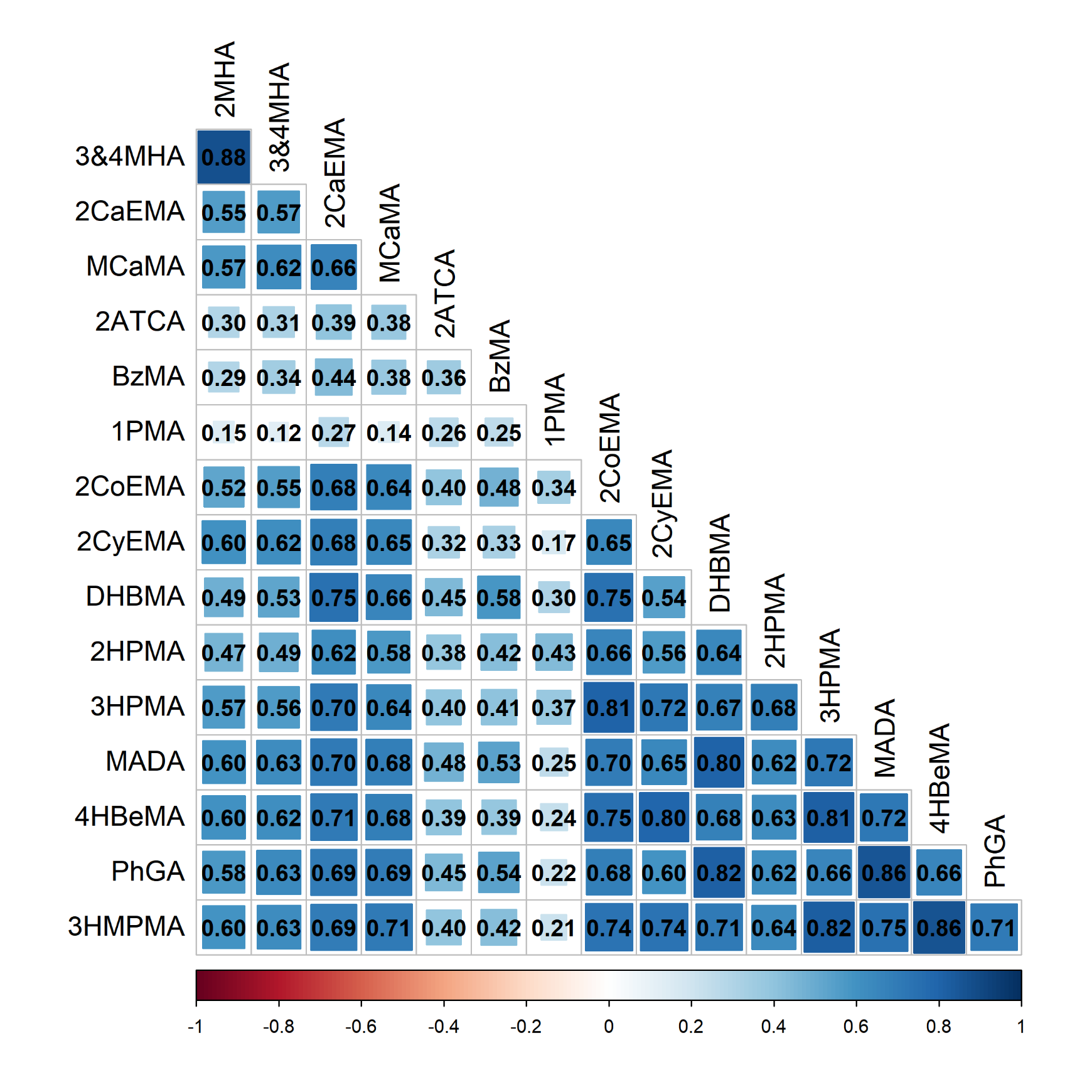


**Figure S2. Spearman correlation coefficients for VOCMs.**

**Table S1. Summary of all VOCMs detected in NHANES cycles**

| VOCMs | Full name | LLOD | Detection rate (%) | 2011-2012* (N=9756) | 2013-2014* (N=10175) | 2015-2016* (N=9971) | 2017-2020* (N=15560) | Included |
| --- | --- | --- | --- | --- | --- | --- | --- | --- |
| 2MHA | 2-methylhippuric acid | 5 | 90.61% | 2466 | 2370 | 3015 | 4325 | √ |
| 3&4MHA | 3-methylhippuric acid and 4-methylhippuric acid | 8 | 99.49% | 2466 | 2628 | 3014 | 4325 | √ |
| 2CaEMA | N-acetyl-S-(2-carbamoylethyl)-L-cysteine | 2.2 | 99.90% | 2466 | 2511 | 3015 | 4325 | √ |
| MCaMA | N-acetyl-S-(N-methylcarbamoyl)-L-cysteine | 6.26 | 99.53% | 2466 | 2629 | 2965 | 4325 | √ |
| 2ATCA | 2-aminothiazoline-4-carboxylic acid | 15 | 94.19% | 2466 | 2523 | 3015 | 4325 | √ |
| BzMA | N-acetyl-S-(benzyl)-L-cysteine | 0.5 | 99.45% | 2466 | 2622 | 3014 | 4325 | √ |
| 1PMA | N-acetyl-S-(n-propyl)-L-cysteine | 1.2 | 73.53% | 2396 | 2631 | 2952 | 4325 | √ |
| 2CoEMA | N-acetyl-S-(2-carboxyethyl)-L-cysteine | 6.96 | 99.03% | 2466 | 2609 | 3015 | 4325 | √ |
| 2CyEMA | N-acetyl-S-(2-cyanoethyl)-L-cysteine | 0.5 | 84.23% | 2466 | 2576 | 3015 | 4325 | √ |
| DHBMA | N-acetyl-S-(3,4-dihydroxybutyl)-L-cysteine | 5.25 | 99.98% | 2466 | 2639 | 2691 | 4325 | √ |
| 2HPMA | N-acetyl-S-(2-hydroxypropyl)-L-cysteine | 5.3 | 94.61% | 2466 | 2597 | 3014 | 4325 | √ |
| 3HPMA | N-acetyl-S-(3-hydroxypropyl)-L-cysteine | 13 | 99.78% | 2466 | 2606 | 2821 | 4325 | √ |
| MADA | Mandelic acid | 12 | 98.64% | 2466 | 2541 | 3008 | 4325 | √ |
| 4HBeMA | N-acetyl-S-(4-hydroxy-2-butenyl)-L-cysteine | 0.6 | 96.84% | 2466 | 2520 | 3015 | 4325 | √ |
| PhGA | Phenylglyoxylic acid | 12 | 99.42% | 2466 | 2575 | 3013 | 4325 | √ |
| 3HMPMA | N-acetyl-S-(3-hydroxypropyl-1-methyl)-L-cysteine | 1.13 | 99.99% | 2466 | 2593 | 3015 | 4325 | √ |
| 2CaHEMA | N-acetyl-S-(2-carbamoyl-2-hydroxyethyl)-L-cysteine | 9.4 | 41.49% | 2466 | 2636 | 3015 | 4325 | × |
| 2HEMA | N-acetyl-S-(2-hydroxyethyl)-L-cysteine | 0.791 | 50.15% | 2466 | 2586 | 3015 | 4325 | × |
| 1,2DCVMA | N-acetyl-S-(1,2-dichlorovinyl)-L-cysteine | 12.6 | 0.04% | 2466 | 2475 | 2886 | - | × |
| 2,2DCVMA | N-acetyl-S-(2,2-dichlorovinyl)-L-cysteine | 4.7 | 0.10% | 2466 | 2551 | 2831 | - | × |
| DPMA | N-acetyl-S-(dimethylphenyl)-L-cysteine | 0.5 | 0.24% | 2466 | 2471 | 3015 | - | × |
| MHBMA1 | N-acetyl-S-(1-hydroxymethyl-2-propenyl)-L-cysteine | 0.7 | 0.65% | 2466 | 2587 | 2987 | - | × |
| MHBMA2 | N-acetyl-S-(2-hydroxy-3-butenyl)-L-cysteine | 0.7 | 9.95% | 2466 | 2627 | 3015 | - | × |
| PHEM | N-acetyl-S-(phenyl-2-hydroxyethyl)-L-cysteine | 0.7 | 40.90% | 2466 | 2569 | 2947 | - | × |
| PMA | N-acetyl-S-(phenyl)-L-cysteine | 0.6 | 44.18% | 2466 | 2561 | 3015 | - | × |
| TCVMA | N-acetyl-S-(trichlorovinyl)-L-cysteine | 3 | 0.02% | 2466 | 2619 | 3015 | - | × |
| TTCA | 2-Thioxothiazolidine-4-carboxylic acid | 11.2 | 35.27% | 2466 | 2640 | - | 4325 | × |
| CYHA | N-acetyl-S-(1-cyano-2-hydroxyethyl)-L-cysteine | 2.6 | 15.16% | - | - | 3015 | 4325 | × |
| IPM1 | N-acetyl-S-(2-hydroxy-3-methyl-3-butenyl)-L-cysteine+N-acetyl-S-(2-hydroxy-2-methyl-3-butenyl)-L-cysteine | 1.43 | 73.80% | - | - | 3015 | - | × |
| IPM3 | N-acetyl-S-(4-hydroxy-2-methyl-2-butenyl)-L-cysteine | 1.2 | 81.55% | - | - | 3015 | 4324 | × |

* The number indicates the total number of samples detected in the cycle. - indicates VOCMs were not detected in this cycle.

2CaHEMA and 2HEMA were excluded due to detection rate < 60%. Other VOCMs were excluded because they were not detected in all cycles.

Abbreviations: LLOD, lower limit of detection.

**Table S2. Variables used in calculating KDM-BA, PA, HD and AL**

| Variables | KDM-BA calculation | PA calculation | AL calculation | HD calculation |
| --- | --- | --- | --- | --- |
| Chronological age | √(units: years) | √(units: years) |  |  |
| Lymphocyte percent |  | √(units: %) |  | √(units: %) |
| Mean cell volume |  | √(units: fL) |  | √(units: fL) |
| Red cell distribution width |  | √(units: %) |  | √(units: %) |
| White blood cell count |  | √(units: 1000 cells/mL) | √(units: 1000 cells/mL) | √(units: 1000 cells/mL) |
| C-reactive protein* | √(Ln, units: mg/dL) | √(Ln, units: mg/dL) | √(units: mg/dL) | √(Ln, units: mg/dL) |
| Serum creatinine | √(units: mg/dL) | √(units: mmol/L) | √(units: mg/dL) | √(units: mg/dL) |
| Blood urea nitrogen | √(units: mg/dL) |  |  |  |
| Serum albumin | √(units: g/dL) | √(units: g/L) |  | √(units: g/dL) |
| Serum alanine aminotransferase |  |  | √(units: U/L) |  |
| Serum aspartate aminotransferase |  |  | √(units: U/L) |  |
| Serum γ-glutamyl transferase |  |  | √(units: U/L) |  |
| Serum alkaline phosphatase | √(units: U/L) | √(units: U/L) |  | √(units: U/L) |
| Blood glucose |  | √(units: mmol/L) |  | √(units: mg/dL) |
| Glycated hemoglobin | √(units: %) |  | √(units: %) |  |
| Serum total cholesterol | √(units: mg/dL) |  |  |  |
| Triglyceride |  |  | √(units: mg/dL) |  |
| Low-density lipoprotein cholesterol |  |  | √(units: mg/dL) |  |
| High-density lipoprotein cholesterol |  |  | √(units: mg/dL) |  |
| Systolic blood pressure | √(units: mmHg) |  | √(units: mmHg) |  |
| Diastolic blood pressure |  |  | √(units: mmHg) |  |
| Heart rate |  |  | √(units: bpm) |  |

* C-reactive protein was not detected in NHANES 2011-2012 and 2013-2014 cycles.

Abbreviations: KDM-BA, Klemera-Doubal method biological age; PA, phenotypic age; AL, allostatic load; HD, homeostatic dysregulation; Ln, natural logarithmic transformation.

**Table S3. Components of several healthy dietary indexes**

| **MEDI** | **HEI-2020** | **AHEI-2010** | **DASH** |
| --- | --- | --- | --- |
| **Adequacy components** |  |  |  |
| Vegetable | Total vegetable | Vegetables | Vegetable |
| Whole fruit + fruit juice | Total fruit | Whole fruit | Whole fruit + fruit juice |
| Whole grain products | Whole fruit | Whole grains | Nuts/Legumes/ vegetable protein |
| Legume | Greens and beans | Nuts/legumes/vegetable protein | Whole grains |
| Nut | Total protein food | Long chain (omega-3) fats (EPA+DHA) | Low-fat dairy |
| Fish | Seafood and plant protein | Polyunsaturated fatty acids |  |
| Ratio of monounsaturated fatty acids to  saturated fatty acids | Whole grains |  |  |
|  | Dairy |  |  |
|  | fatty acids |  |  |
| **Moderation components** |  |  |  |
| Red and processed meat | Refined grains | Red/processed meat | Red/processed meat |
| Alcohol (optimum, 10-25 g/day) | Added sugars | Sugar-sweetened beverages | Sugar-sweetened beverages |
|  | Saturated fat |  | Sodium |
|  | Sodium |  |  |
| **Score range** |  |  |  |
| 0-9 | 0-100 | 0-100 | 8-40 |

Abbreviations: DII, Dietary Inflammatory Index; MED, Mediterranean Diet Index; HEI-2020, Healthy Eating Index-2020; AHEI-2010, Alternative Healthy Eating Index-2010; DASH, Dietary Approaches to Stop Hypertension Index; EPA, eicosapentaenoic acid; DHA, docosahexaenoic acid.

**Table S4. Variance inflation factors (VIFs) of covariates in the survey-weighted multivariable linear regression models for KDMAgeAccel.**

| **VOCMs** | **Covariate** | | | | |
| --- | --- | --- | --- | --- | --- |
|  | **Sex** | **Age** | **BMI** | **Race** | **Education level** |
| 2MHA | 1.561 | 2.019 | 1.708 | 1.435 | 1.404 |
| 3&4MHA | 1.540 | 2.015 | 1.747 | 1.430 | 1.412 |
| 2CaEMA | 1.512 | 2.169 | 1.707 | 1.435 | 1.397 |
| MCaMA | 1.575 | 2.257 | 1.728 | 1.453 | 1.408 |
| 2ATCA | 1.583 | 2.153 | 1.706 | 1.442 | 1.427 |
| BzMA | 1.532 | 2.138 | 1.699 | 1.430 | 1.405 |
| 1PMA | 1.497 | 2.044 | 1.683 | 1.450 | 1.384 |
| 2CoEMA | 1.509 | 2.136 | 1.724 | 1.422 | 1.453 |
| 2CyEMA | 1.510 | 2.091 | 1.703 | 1.435 | 1.443 |
| DHBMA | 1.497 | 2.272 | 1.740 | 1.460 | 1.413 |
| 2HPMA | 1.502 | 2.053 | 1.708 | 1.432 | 1.392 |
| 3HPMA | 1.525 | 2.223 | 1.709 | 1.434 | 1.428 |
| MADA | 1.496 | 2.199 | 1.690 | 1.437 | 1.432 |
| 4HBeMA | 1.498 | 2.232 | 1.716 | 1.420 | 1.425 |
| PhGA | 1.498 | 2.114 | 1.640 | 1.434 | 1.417 |
| 3HMPMA | 1.510 | 2.117 | 1.701 | 1.441 | 1.439 |
|  |  |  |  |  |  |
|  | **Marital status** | **PIR** | **Smoking status** | **Drinking status** | **MET** |
| 2MHA | 1.345 | 1.478 | 1.512 | 1.368 | 1.329 |
| 3&4MHA | 1.334 | 1.485 | 1.542 | 1.377 | 1.328 |
| 2CaEMA | 1.333 | 1.479 | 1.548 | 1.363 | 1.327 |
| MCaMA | 1.322 | 1.482 | 1.629 | 1.371 | 1.395 |
| 2ATCA | 1.336 | 1.490 | 1.531 | 1.378 | 1.349 |
| BzMA | 1.343 | 1.524 | 1.498 | 1.391 | 1.333 |
| 1PMA | 1.363 | 1.556 | 1.477 | 1.357 | 1.349 |
| 2CoEMA | 1.362 | 1.537 | 1.547 | 1.373 | 1.312 |
| 2CyEMA | 1.340 | 1.518 | 1.889 | 1.365 | 1.330 |
| DHBMA | 1.346 | 1.526 | 1.480 | 1.379 | 1.315 |
| 2HPMA | 1.373 | 1.520 | 1.519 | 1.419 | 1.359 |
| 3HPMA | 1.336 | 1.483 | 1.686 | 1.355 | 1.348 |
| MADA | 1.369 | 1.561 | 1.476 | 1.366 | 1.332 |
| 4HBeMA | 1.388 | 1.498 | 1.618 | 1.364 | 1.330 |
| PhGA | 1.347 | 1.472 | 1.497 | 1.337 | 1.337 |
| 3HMPMA | 1.344 | 1.539 | 1.679 | 1.376 | 1.341 |
|  |  |  |  |  |  |
|  | **Dietary calories** | **Diabetes** | **Hypertension** | **Hyperlipidemia** | **urinary creatinine** |
| 2MHA | 1.398 | 1.436 | 1.913 | 1.639 | 1.541 |
| 3&4MHA | 1.418 | 1.426 | 1.903 | 1.613 | 1.583 |
| 2CaEMA | 1.418 | 1.418 | 2.012 | 1.730 | 1.916 |
| MCaMA | 1.447 | 1.557 | 1.911 | 1.628 | 2.113 |
| 2ATCA | 1.465 | 1.452 | 1.866 | 1.596 | 1.776 |
| BzMA | 1.447 | 1.415 | 1.894 | 1.562 | 1.805 |
| 1PMA | 1.402 | 1.406 | 1.909 | 1.567 | 1.585 |
| 2CoEMA | 1.426 | 1.471 | 1.962 | 1.575 | 1.920 |
| 2CyEMA | 1.430 | 1.457 | 1.907 | 1.622 | 1.597 |
| DHBMA | 1.411 | 1.430 | 1.910 | 1.628 | 2.364 |
| 2HPMA | 1.407 | 1.413 | 1.896 | 1.580 | 1.534 |
| 3HPMA | 1.409 | 1.445 | 1.948 | 1.667 | 1.756 |
| MADA | 1.388 | 1.501 | 1.881 | 1.677 | 2.063 |
| 4HBeMA | 1.438 | 1.419 | 1.882 | 1.619 | 1.691 |
| PhGA | 1.358 | 1.406 | 1.929 | 1.568 | 1.999 |
| 3HMPMA | 1.397 | 1.439 | 1.886 | 1.572 | 1.744 |

**Table S5. Detection rates and distribution of included VOCMs cross cycles**

| VOCMs | Detection rate (%) | GM (GSE) | Percentile | | | | |
| --- | --- | --- | --- | --- | --- | --- | --- |
|  |  |  | 5th | 25th | 50th | 75th | 95th |
| 2MHA | 91.98% | 29.86 (1.04) | 3.5 | 12.3 | 27.9 | 73.7 | 237.0 |
| 3&4MHA | 99.60% | 182.79 (1.04) | 23.9 | 71.0 | 168.0 | 470.0 | 1560.0 |
| 2CaEMA | 99.90% | 51.72 (1.03) | 9.4 | 26.4 | 50.1 | 99.9 | 291.0 |
| MCaMA | 99.84% | 151.68 (1.03) | 25.5 | 75.2 | 154.0 | 315.0 | 872.0 |
| 2ATCA | 92.00% | 97.71 (1.03) | 20.2 | 48.0 | 102.0 | 200.0 | 510.0 |
| BzMA | 99.56% | 6.27 (1.02) | 1.2 | 3.2 | 6.1 | 11.4 | 38.2 |
| 1PMA | 77.69% | 4.21 (1.03) | 0.9 | 1.3 | 3.7 | 10.4 | 44.0 |
| 2CoEMA | 99.18% | 91.36 (1.03) | 16.5 | 47.2 | 93.5 | 178.0 | 506.0 |
| 2CyEMA | 86.33% | 3.32 (1.05) | 0.4 | 0.8 | 1.6 | 7.1 | 262.0 |
| DHBMA | 99.98% | 291.78 (1.02) | 72.6 | 176.0 | 318.0 | 515.0 | 901.0 |
| 2HPMA | 95.78% | 31.97 (1.03) | 5.8 | 15.6 | 29.4 | 59.4 | 225.0 |
| 3HPMA | 99.88% | 248.81 (1.03) | 44.0 | 116.0 | 237.0 | 472.0 | 1950.0 |
| MADA | 99.04% | 128.02 (1.03) | 27.7 | 72.0 | 132.0 | 233.0 | 511.0 |
| 4HBeMA | 97.39% | 5.24 (1.04) | 0.9 | 2.4 | 4.6 | 10.1 | 55.0 |
| PhGA | 99.52% | 195.77 (1.02) | 43.4 | 117.0 | 208.0 | 355.0 | 723.0 |
| 3HMPMA | 100.00% | 246.96 (1.03) | 49.4 | 118.0 | 227.0 | 434.0 | 2070.0 |

All GM (GSE) were survey-weighted.

Abbreviations: GM, geometric mean; GSE, geometric standard error; 2MHA, 2-methylhippuric acid; 3&4MHA, 3- and 4-methylhippuric acid; 2CaEMA, N-acetyl-S-(2-carbamoylethyl)-L-cysteine; MCaMA, N-acetyl-S-(N-methylcarbamoyl)-L-cysteine; 2ATCA, 2-aminothiazoline-4-carboxylic acid; BzMA, N-acetyl-S-(benzyl)-L-cysteine; 1PMA, N-acetyl-S-(n-propyl)-L-cysteine; 2CoEMA, N-acetyl-S-(2-carboxyethyl)-L-cysteine; 2CyEMA, N-acetyl-S-(2-cyanoethyl)-L-cysteine; DHBMA, N-acetyl-S-(3,4-dihydroxybutyl)-L-cysteine; 2HPMA, N-acetyl-S-(2-hydroxypropyl)-L-cysteine; 3HPMA, N-acetyl-S-(3-hydroxypropyl)-L-cysteine; MADA, mandelic acid; 4HBeMA, N-acetyl-S-(4-hydroxy-2-butenyl)-L-cysteine; PhGA, phenylglyoxylic acid; 3HMPMA, N-acetyl-S-(3-hydroxypropyl-1-methyl)-L-cysteine.

**Table S6. BA indicators and VOCM concentrations of subjects by median DII**

|  | Total (N=4975*) | DII≤1.29 (N=2487) | DII>1.29 (N=2488) | *P* |
| --- | --- | --- | --- | --- |
| KDMAgeAccel (median [IQR]) | -1.59 [-8.45, 6.45] | -2.91 [-9.77, 5.22] | -0.10 [-6.86, 7.45] | **<0.001** |
| PhenoAgeAccel (median [IQR]) | 0.66 [-1.86, 4.12] | 0.22 [-2.20, 3.47] | 1.16 [-1.57, 4.87] | **0.008** |
| AL (median [IQR]) | 0.20 [0.07, 0.33] | 0.20 [0.07, 0.32] | 0.22 [0.10, 0.37] | **<0.001** |
| HD (median [IQR]) | 3.12 [2.66, 3.81] | 3.08 [2.60, 3.65] | 3.23 [2.70, 3.97] | **0.011** |
| 2MHA (median [IQR]) | 27.90 [12.30, 73.70] | 25.10 [11.70, 64.63] | 31.83 [13.64, 87.60] | **0.001** |
| 3&4MHA (median [IQR]) | 168.00 [70.95, 469.96] | 147.00 [63.70, 391.00] | 196.00 [81.21, 561.52] | **<0.001** |
| 2CaEMA (median [IQR]) | 50.10 [26.40, 99.84] | 46.98 [24.70, 93.10] | 53.15 [28.40, 111.00] | **0.004** |
| MCaMA (median [IQR]) | 154.00 [75.14, 315.00] | 137.00 [68.40, 272.08] | 172.00 [83.06, 357.00] | **<0.001** |
| 2ATCA (median [IQR]) | 102.00 [48.00, 200.00] | 91.30 [43.60, 180.00] | 116.00 [52.90, 224.00] | **<0.001** |
| BzMA (median [IQR]) | 6.12 [3.18, 11.40] | 6.26 [3.23, 11.50] | 5.92 [3.14, 11.30] | 0.538 |
| 1PMA (median [IQR]) | 3.67 [1.33, 10.40] | 4.01 [1.49, 10.90] | 3.28 [0.85, 9.61] | **0.010** |
| 2CoEMA (median [IQR]) | 93.44 [47.16, 178.00] | 89.84 [43.30, 166.00] | 96.11 [51.10, 192.00] | **0.013** |
| 2CyEMA (median [IQR]) | 1.60 [0.76, 7.04] | 1.42 [0.72, 3.62] | 1.84 [0.82, 39.71] | **<0.001** |
| DHBMA (median [IQR]) | 318.00 [176.00, 515.00] | 308.00 [160.00, 499.00] | 327.00 [190.00, 525.00] | **0.013** |
| 2HPMA (median [IQR]) | 29.40 [15.60, 59.40] | 31.10 [15.90, 59.10] | 27.80 [15.40, 60.00] | 0.659 |
| 3HPMA (median [IQR]) | 237.00 [116.00, 472.00] | 226.00 [112.00, 419.00] | 252.65 [120.00, 565.51] | **0.008** |
| MADA (median [IQR]) | 132.00 [72.00, 233.00] | 126.00 [68.15, 220.00] | 141.00 [78.18, 246.00] | **0.002** |
| 4HBeMA (median [IQR]) | 4.56 [2.40, 10.10] | 4.34 [2.28, 8.77] | 4.82 [2.55, 13.17] | **<0.001** |
| PhGA (median [IQR]) | 208.00 [117.00, 354.92] | 200.00 [107.63, 342.00] | 221.00 [125.00, 365.00] | **0.003** |
| 3HMPMA (median [IQR]) | 227.00 [118.00, 434.00] | 215.00 [109.00, 385.24] | 237.00 [125.00, 510.00] | **0.006** |

All continuous variables were expressed as weighted medians (25, 75th percentiles). ^*^1 subject with missing data on DII.

Abbreviations: KDMAgeAccel, Klemera-Doubal method biological age acceleration; PhenoAgeAccel, phenotypic age acceleration; AL, allostatic load; HD, homeostatic dysregulation; 2MHA, 2-methylhippuric acid; 3&4MHA, 3- and 4-methylhippuric acid; 2CaEMA, N-acetyl-S-(2-carbamoylethyl)-L-cysteine; MCaMA, N-acetyl-S-(N-methylcarbamoyl)-L-cysteine; 2ATCA, 2-aminothiazoline-4-carboxylic acid; BzMA, N-acetyl-S-(benzyl)-L-cysteine; 1PMA, N-acetyl-S-(n-propyl)-L-cysteine; 2CoEMA, N-acetyl-S-(2-carboxyethyl)-L-cysteine; 2CyEMA, N-acetyl-S-(2-cyanoethyl)-L-cysteine; DHBMA, N-acetyl-S-(3,4-dihydroxybutyl)-L-cysteine; 2HPMA, N-acetyl-S-(2-hydroxypropyl)-L-cysteine; 3HPMA, N-acetyl-S-(3-hydroxypropyl)-L-cysteine; MADA, mandelic acid; 4HBeMA, N-acetyl-S-(4-hydroxy-2-butenyl)-L-cysteine; PhGA, phenylglyoxylic acid; 3HMPMA, N-acetyl-S-(3-hydroxypropyl-1-methyl)-L-cysteine.

**Table S7. BA indicators and VOCM concentrations of subjects by moderate to high adherence to MEDI**

|  | Total (N=4975*) | MEDI<4 (N=3001) | MEDI≥4 (N=1974) | *P* |
| --- | --- | --- | --- | --- |
| KDMAgeAccel (median [IQR]) | -1.59 [-8.45, 6.45] | -0.24 [-7.45, 7.92] | -3.33 [-9.99, 4.56] | **<0.001** |
| PhenoAgeAccel (median [IQR]) | 0.66 [-1.86, 4.12] | 1.29 [-1.22, 4.84] | -0.14 [-2.78, 2.83] | **<0.001** |
| AL (median [IQR]) | 0.20 [0.07, 0.33] | 0.23 [0.10, 0.37] | 0.17 [0.07, 0.30] | **<0.001** |
| HD (median [IQR]) | 3.12 [2.66, 3.81] | 3.19 [2.67, 3.90] | 3.06 [2.65, 3.64] | **0.045** |
| 2MHA (median [IQR]) | 27.90 [12.30, 73.70] | 34.30 [14.80, 87.88] | 21.10 [9.70, 54.28] | **<0.001** |
| 3&4MHA (median [IQR]) | 168.00 [70.95, 469.96] | 201.00 [87.30, 554.00] | 124.00 [56.20, 349.00] | **<0.001** |
| 2CaEMA (median [IQR]) | 50.10 [26.40, 99.84] | 57.20 [30.67, 117.40] | 42.02 [21.70, 80.01] | **<0.001** |
| MCaMA (median [IQR]) | 154.00 [75.14, 315.00] | 183.00 [90.36, 355.00] | 121.09 [56.84, 240.00] | **<0.001** |
| 2ATCA (median [IQR]) | 102.00 [48.00, 200.00] | 112.00 [53.33, 218.00] | 86.78 [39.95, 172.08] | **<0.001** |
| BzMA (median [IQR]) | 6.12 [3.18, 11.40] | 6.11 [3.34, 11.20] | 6.14 [3.00, 11.70] | 0.978 |
| 1PMA (median [IQR]) | 3.67 [1.33, 10.40] | 3.60 [1.27, 10.50] | 3.75 [1.44, 10.23] | 0.334 |
| 2CoEMA (median [IQR]) | 93.44 [47.16, 178.00] | 105.00 [55.50, 199.00] | 76.50 [37.90, 149.00] | **<0.001** |
| 2CyEMA (median [IQR]) | 1.60 [0.76, 7.04] | 1.92 [0.90, 41.35] | 1.23 [0.60, 2.64] | **<0.001** |
| DHBMA (median [IQR]) | 318.00 [176.00, 515.00] | 347.00 [205.17, 555.00] | 272.00 [146.00, 460.00] | **<0.001** |
| 2HPMA (median [IQR]) | 29.40 [15.60, 59.40] | 30.12 [16.60, 60.70] | 27.88 [14.30, 57.97] | **0.035** |
| 3HPMA (median [IQR]) | 237.00 [116.00, 472.00] | 268.00 [130.00, 579.00] | 191.00 [95.10, 365.23] | **<0.001** |
| MADA (median [IQR]) | 132.00 [72.00, 233.00] | 150.00 [84.35, 255.00] | 111.00 [58.10, 205.00] | **<0.001** |
| 4HBeMA (median [IQR]) | 4.56 [2.40, 10.10] | 5.29 [2.71, 13.30] | 3.70 [2.00, 7.13] | **<0.001** |
| PhGA (median [IQR]) | 208.00 [117.00, 354.92] | 234.00 [131.00, 380.00] | 179.00 [95.33, 309.00] | **<0.001** |
| 3HMPMA (median [IQR]) | 227.00 [118.00, 434.00] | 254.00 [134.00, 510.00] | 191.78 [99.72, 345.00] | **<0.001** |

All continuous variables were expressed as weighted medians (25, 75th percentiles). ^*^1 subject with missing data on MEDI.

Abbreviations: KDMAgeAccel, Klemera-Doubal method biological age acceleration; PhenoAgeAccel, phenotypic age acceleration; AL, allostatic load; HD, homeostatic dysregulation; 2MHA, 2-methylhippuric acid; 3&4MHA, 3- and 4-methylhippuric acid; 2CaEMA, N-acetyl-S-(2-carbamoylethyl)-L-cysteine; MCaMA, N-acetyl-S-(N-methylcarbamoyl)-L-cysteine; 2ATCA, 2-aminothiazoline-4-carboxylic acid; BzMA, N-acetyl-S-(benzyl)-L-cysteine; 1PMA, N-acetyl-S-(n-propyl)-L-cysteine; 2CoEMA, N-acetyl-S-(2-carboxyethyl)-L-cysteine; 2CyEMA, N-acetyl-S-(2-cyanoethyl)-L-cysteine; DHBMA, N-acetyl-S-(3,4-dihydroxybutyl)-L-cysteine; 2HPMA, N-acetyl-S-(2-hydroxypropyl)-L-cysteine; 3HPMA, N-acetyl-S-(3-hydroxypropyl)-L-cysteine; MADA, mandelic acid; 4HBeMA, N-acetyl-S-(4-hydroxy-2-butenyl)-L-cysteine; PhGA, phenylglyoxylic acid; 3HMPMA, N-acetyl-S-(3-hydroxypropyl-1-methyl)-L-cysteine.

**Table S8. BA indicators and VOCM concentrations of subjects by median HEI-2020**

|  | Total (N=4976) | HEI-2020≤50.46 (N=2488) | HEI-2020>50.46 (N=2488) | *P* |
| --- | --- | --- | --- | --- |
| KDMAgeAccel (median [IQR]) | -1.59 [-8.45, 6.45] | 0.12 [-7.14, 7.77] | -3.14 [-9.74, 4.93] | **<0.001** |
| PhenoAgeAccel (median [IQR]) | 0.66 [-1.86, 4.12] | 1.62 [-1.18, 5.04] | -0.11 [-2.68, 2.90] | **<0.001** |
| AL (median [IQR]) | 0.20 [0.07, 0.33] | 0.23 [0.10, 0.37] | 0.20 [0.07, 0.30] | **<0.001** |
| HD (median [IQR]) | 3.12 [2.66, 3.81] | 3.20 [2.69, 3.87] | 3.07 [2.65, 3.71] | 0.088 |
| 2MHA (median [IQR]) | 27.90 [12.30, 73.70] | 35.00 [15.36, 96.05] | 22.50 [10.50, 56.52] | **<0.001** |
| 3&4MHA (median [IQR]) | 168.00 [70.95, 469.96] | 207.21 [89.64, 597.00] | 135.00 [57.40, 347.50] | **<0.001** |
| 2CaEMA (median [IQR]) | 50.10 [26.40, 99.84] | 60.43 [32.10, 127.00] | 42.33 [22.26, 80.68] | **<0.001** |
| MCaMA (median [IQR]) | 154.00 [75.14, 315.00] | 194.00 [92.57, 375.00] | 126.00 [62.20, 244.00] | **<0.001** |
| 2ATCA (median [IQR]) | 102.00 [48.00, 200.00] | 113.00 [54.50, 217.00] | 90.18 [42.60, 183.45] | **<0.001** |
| BzMA (median [IQR]) | 6.12 [3.18, 11.40] | 6.09 [3.31, 11.20] | 6.21 [3.08, 11.60] | 0.551 |
| 1PMA (median [IQR]) | 3.67 [1.33, 10.40] | 3.73 [1.38, 10.90] | 3.60 [1.27, 9.67] | 0.215 |
| 2CoEMA (median [IQR]) | 93.44 [47.16, 178.00] | 106.00 [58.13, 206.00] | 80.35 [39.40, 155.00] | **<0.001** |
| 2CyEMA (median [IQR]) | 1.60 [0.76, 7.04] | 2.06 [0.91, 53.19] | 1.25 [0.62, 2.79] | **<0.001** |
| DHBMA (median [IQR]) | 318.00 [176.00, 515.00] | 353.00 [208.00, 558.00] | 280.47 [148.00, 474.00] | **<0.001** |
| 2HPMA (median [IQR]) | 29.40 [15.60, 59.40] | 32.13 [17.50, 65.90] | 26.60 [13.80, 53.00] | **<0.001** |
| 3HPMA (median [IQR]) | 237.00 [116.00, 472.00] | 279.00 [136.00, 610.95] | 195.00 [95.29, 377.00] | **<0.001** |
| MADA (median [IQR]) | 132.00 [72.00, 233.00] | 151.00 [86.60, 255.00] | 115.18 [62.00, 212.99] | **<0.001** |
| 4HBeMA (median [IQR]) | 4.56 [2.40, 10.10] | 5.43 [2.86, 14.27] | 3.82 [2.00, 7.59] | **<0.001** |
| PhGA (median [IQR]) | 208.00 [117.00, 354.92] | 237.00 [136.00, 389.00] | 184.00 [98.90, 316.00] | **<0.001** |
| 3HMPMA (median [IQR]) | 227.00 [118.00, 434.00] | 254.00 [138.00, 532.34] | 199.00 [101.00, 373.13] | **<0.001** |

All continuous variables were expressed as weighted medians (25, 75th percentiles).

Abbreviations: KDMAgeAccel, Klemera-Doubal method biological age acceleration; PhenoAgeAccel, phenotypic age acceleration; AL, allostatic load; HD, homeostatic dysregulation; 2MHA, 2-methylhippuric acid; 3&4MHA, 3- and 4-methylhippuric acid; 2CaEMA, N-acetyl-S-(2-carbamoylethyl)-L-cysteine; MCaMA, N-acetyl-S-(N-methylcarbamoyl)-L-cysteine; 2ATCA, 2-aminothiazoline-4-carboxylic acid; BzMA, N-acetyl-S-(benzyl)-L-cysteine; 1PMA, N-acetyl-S-(n-propyl)-L-cysteine; 2CoEMA, N-acetyl-S-(2-carboxyethyl)-L-cysteine; 2CyEMA, N-acetyl-S-(2-cyanoethyl)-L-cysteine; DHBMA, N-acetyl-S-(3,4-dihydroxybutyl)-L-cysteine; 2HPMA, N-acetyl-S-(2-hydroxypropyl)-L-cysteine; 3HPMA, N-acetyl-S-(3-hydroxypropyl)-L-cysteine; MADA, mandelic acid; 4HBeMA, N-acetyl-S-(4-hydroxy-2-butenyl)-L-cysteine; PhGA, phenylglyoxylic acid; 3HMPMA, N-acetyl-S-(3-hydroxypropyl-1-methyl)-L-cysteine.

**Table S9. BA indicators and VOCM concentrations of subjects by median AHEI-2010**

|  | Total (N=4975*) | AHEI-2010≤37.53 (N=2487) | AHEI-2010>37.53 (N=2488) | *P* |
| --- | --- | --- | --- | --- |
| KDMAgeAccel (median [IQR]) | -1.59 [-8.45, 6.45] | 0.11 [-7.13, 8.00] | -2.94 [-9.87, 5.05] | **<0.001** |
| PhenoAgeAccel (median [IQR]) | 0.66 [-1.86, 4.12] | 1.61 [-0.71, 5.08] | -0.24 [-2.62, 3.02] | **<0.001** |
| AL (median [IQR]) | 0.20 [0.07, 0.33] | 0.23 [0.12, 0.38] | 0.18 [0.07, 0.30] | **<0.001** |
| HD (median [IQR]) | 3.12 [2.66, 3.81] | 3.21 [2.69, 3.91] | 3.07 [2.65, 3.66] | **0.046** |
| 2MHA (median [IQR]) | 27.90 [12.30, 73.70] | 36.60 [16.60, 95.60] | 21.36 [10.00, 55.60] | **<0.001** |
| 3&4MHA (median [IQR]) | 168.00 [70.95, 469.96] | 221.00 [96.16, 611.64] | 128.00 [56.20, 333.52] | **<0.001** |
| 2CaEMA (median [IQR]) | 50.10 [26.40, 99.84] | 60.35 [30.92, 132.00] | 43.56 [23.00, 82.34] | **<0.001** |
| MCaMA (median [IQR]) | 154.00 [75.14, 315.00] | 186.00 [90.10, 384.21] | 127.00 [63.41, 261.00] | **<0.001** |
| 2ATCA (median [IQR]) | 102.00 [48.00, 200.00] | 116.00 [52.60, 224.00] | 89.88 [43.60, 181.34] | **<0.001** |
| BzMA (median [IQR]) | 6.12 [3.18, 11.40] | 6.19 [3.39, 11.70] | 6.06 [3.05, 11.40] | 0.593 |
| 1PMA (median [IQR]) | 3.67 [1.33, 10.40] | 3.72 [1.29, 10.60] | 3.59 [1.35, 10.13] | 0.862 |
| 2CoEMA (median [IQR]) | 93.44 [47.16, 178.00] | 110.00 [58.99, 209.00] | 79.05 [39.40, 153.00] | **<0.001** |
| 2CyEMA (median [IQR]) | 1.60 [0.76, 7.04] | 2.16 [0.95, 59.87] | 1.24 [0.61, 2.64] | **<0.001** |
| DHBMA (median [IQR]) | 318.00 [176.00, 515.00] | 367.03 [208.00, 572.00] | 278.00 [152.00, 461.00] | **<0.001** |
| 2HPMA (median [IQR]) | 29.40 [15.60, 59.40] | 32.36 [17.40, 63.50] | 26.60 [14.30, 55.12] | **0.002** |
| 3HPMA (median [IQR]) | 237.00 [116.00, 472.00] | 290.00 [142.00, 627.52] | 191.00 [95.80, 375.00] | **<0.001** |
| MADA (median [IQR]) | 132.00 [72.00, 233.00] | 153.00 [85.70, 264.00] | 117.00 [62.00, 209.00] | **<0.001** |
| 4HBeMA (median [IQR]) | 4.56 [2.40, 10.10] | 5.67 [2.97, 15.80] | 3.86 [2.06, 7.24] | **<0.001** |
| PhGA (median [IQR]) | 208.00 [117.00, 354.92] | 237.00 [134.00, 393.00] | 189.00 [101.00, 316.00] | **<0.001** |
| 3HMPMA (median [IQR]) | 227.00 [118.00, 434.00] | 264.38 [142.00, 552.77] | 194.00 [101.00, 358.00] | **<0.001** |

All continuous variables were expressed as weighted medians (25, 75th percentiles). ^*^1 subject with missing data on AHEI-2010.

Abbreviations: KDMAgeAccel, Klemera-Doubal method biological age acceleration; PhenoAgeAccel, phenotypic age acceleration; AL, allostatic load; HD, homeostatic dysregulation; 2MHA, 2-methylhippuric acid; 3&4MHA, 3- and 4-methylhippuric acid; 2CaEMA, N-acetyl-S-(2-carbamoylethyl)-L-cysteine; MCaMA, N-acetyl-S-(N-methylcarbamoyl)-L-cysteine; 2ATCA, 2-aminothiazoline-4-carboxylic acid; BzMA, N-acetyl-S-(benzyl)-L-cysteine; 1PMA, N-acetyl-S-(n-propyl)-L-cysteine; 2CoEMA, N-acetyl-S-(2-carboxyethyl)-L-cysteine; 2CyEMA, N-acetyl-S-(2-cyanoethyl)-L-cysteine; DHBMA, N-acetyl-S-(3,4-dihydroxybutyl)-L-cysteine; 2HPMA, N-acetyl-S-(2-hydroxypropyl)-L-cysteine; 3HPMA, N-acetyl-S-(3-hydroxypropyl)-L-cysteine; MADA, mandelic acid; 4HBeMA, N-acetyl-S-(4-hydroxy-2-butenyl)-L-cysteine; PhGA, phenylglyoxylic acid; 3HMPMA, N-acetyl-S-(3-hydroxypropyl-1-methyl)-L-cysteine.

**Table S10. BA indicators and VOCM concentrations of subjects by median** **DASHI**

|  | Total (N=4976) | DASHI≤21.5 (N=2374) | DASHI>21.5 (N=2602) | *P* |
| --- | --- | --- | --- | --- |
| KDMAgeAccel (median [IQR]) | -1.59 [-8.45, 6.45] | 0.26 [-6.68, 8.05] | -3.02 [-9.93, 5.01] | **<0.001** |
| PhenoAgeAccel (median [IQR]) | 0.66 [-1.86, 4.12] | 1.72 [-0.83, 5.11] | -0.17 [-2.67, 2.97] | **<0.001** |
| AL (median [IQR]) | 0.20 [0.07, 0.33] | 0.23 [0.10, 0.38] | 0.20 [0.07, 0.30] | **<0.001** |
| HD (median [IQR]) | 3.12 [2.66, 3.81] | 3.19 [2.67, 3.90] | 3.08 [2.65, 3.69] | 0.131 |
| 2MHA (median [IQR]) | 27.90 [12.30, 73.70] | 36.98 [16.40, 98.07] | 22.57 [9.88, 58.49] | **<0.001** |
| 3&4MHA (median [IQR]) | 168.00 [70.95, 469.96] | 213.93 [92.50, 601.40] | 139.00 [58.32, 376.00] | **<0.001** |
| 2CaEMA (median [IQR]) | 50.10 [26.40, 99.84] | 64.00 [33.20, 133.00] | 42.42 [22.50, 81.18] | **<0.001** |
| MCaMA (median [IQR]) | 154.00 [75.14, 315.00] | 199.47 [94.20, 384.85] | 126.00 [62.88, 248.57] | **<0.001** |
| 2ATCA (median [IQR]) | 102.00 [48.00, 200.00] | 121.00 [56.95, 231.00] | 88.30 [41.52, 181.13] | **<0.001** |
| BzMA (median [IQR]) | 6.12 [3.18, 11.40] | 6.10 [3.47, 11.80] | 6.17 [3.00, 11.30] | 0.486 |
| 1PMA (median [IQR]) | 3.67 [1.33, 10.40] | 3.84 [1.37, 10.60] | 3.46 [1.28, 10.10] | 0.259 |
| 2CoEMA (median [IQR]) | 93.44 [47.16, 178.00] | 110.00 [61.06, 211.00] | 79.80 [39.40, 155.00] | **<0.001** |
| 2CyEMA (median [IQR]) | 1.60 [0.76, 7.04] | 2.19 [0.97, 60.04] | 1.25 [0.61, 2.76] | **<0.001** |
| DHBMA (median [IQR]) | 318.00 [176.00, 515.00] | 372.00 [215.00, 567.00] | 275.00 [151.00, 473.00] | **<0.001** |
| 2HPMA (median [IQR]) | 29.40 [15.60, 59.40] | 31.30 [17.40, 63.69] | 27.59 [14.40, 56.70] | **0.004** |
| 3HPMA (median [IQR]) | 237.00 [116.00, 472.00] | 292.00 [143.00, 638.06] | 194.00 [95.80, 383.00] | **<0.001** |
| MADA (median [IQR]) | 132.00 [72.00, 233.00] | 157.00 [88.33, 261.00] | 114.00 [62.00, 212.00] | **<0.001** |
| 4HBeMA (median [IQR]) | 4.56 [2.40, 10.10] | 5.63 [3.04, 15.60] | 3.86 [2.03, 7.49] | **<0.001** |
| PhGA (median [IQR]) | 208.00 [117.00, 354.92] | 245.00 [139.00, 389.00] | 181.00 [99.50, 320.01] | **<0.001** |
| 3HMPMA (median [IQR]) | 227.00 [118.00, 434.00] | 263.00 [145.00, 556.91] | 199.00 [101.00, 372.00] | **<0.001** |

All continuous variables were expressed as weighted medians (25, 75th percentiles).

Abbreviations: KDMAgeAccel, Klemera-Doubal method biological age acceleration; PhenoAgeAccel, phenotypic age acceleration; AL, allostatic load; HD, homeostatic dysregulation; 2MHA, 2-methylhippuric acid; 3&4MHA, 3- and 4-methylhippuric acid; 2CaEMA, N-acetyl-S-(2-carbamoylethyl)-L-cysteine; MCaMA, N-acetyl-S-(N-methylcarbamoyl)-L-cysteine; 2ATCA, 2-aminothiazoline-4-carboxylic acid; BzMA, N-acetyl-S-(benzyl)-L-cysteine; 1PMA, N-acetyl-S-(n-propyl)-L-cysteine; 2CoEMA, N-acetyl-S-(2-carboxyethyl)-L-cysteine; 2CyEMA, N-acetyl-S-(2-cyanoethyl)-L-cysteine; DHBMA, N-acetyl-S-(3,4-dihydroxybutyl)-L-cysteine; 2HPMA, N-acetyl-S-(2-hydroxypropyl)-L-cysteine; 3HPMA, N-acetyl-S-(3-hydroxypropyl)-L-cysteine; MADA, mandelic acid; 4HBeMA, N-acetyl-S-(4-hydroxy-2-butenyl)-L-cysteine; PhGA, phenylglyoxylic acid; 3HMPMA, N-acetyl-S-(3-hydroxypropyl-1-methyl)-L-cysteine.

**Table S11. Association of VOCMs with** **continuous and binary KDMAgeAccel and AL in weighted generalized linear models**

|  | KDMAgeAccel | |  | KDMAgeAccel>0 | |  | AL | |  | Median AL | |
| --- | --- | --- | --- | --- | --- | --- | --- | --- | --- | --- | --- |
|  | β (95% CI) | *P*-FDR |  | OR (95% CI) | *P*-FDR |  | β (95% CI) | *P*-FDR |  | OR (95% CI) | *P*-FDR |
| 2MHA | **0.036 (0.006,0.066)** | **0.045** |  | 1.083 (1.004,1.168) | 0.080 |  | 0.041 (0.003,0.080) | 0.093 |  | **1.140 (1.034,1.256)** | **0.035** |
| 3&4MHA | 0.010 (-0.019,0.038) | 0.569 |  | 0.996 (0.914,1.085) | 0.972 |  | 0.029 (-0.010,0.069) | 0.205 |  | 1.086 (0.977,1.207) | 0.251 |
| 2CaEMA | 0.000 (-0.044,0.044) | 1.000 |  | 0.997 (0.865,1.150) | 0.972 |  | **-0.108 (-0.157,-0.059)** | **0.001** |  | **0.859 (0.763,0.967)** | **0.035** |
| MCaMA | -0.056 (-0.106,-0.006) | 0.057 |  | 0.898 (0.788,1.023) | 0.164 |  | -0.046 (-0.093,0.001) | 0.111 |  | 0.949 (0.828,1.088) | 0.507 |
| 2ATCA | **0.049 (0.008,0.091)** | **0.045** |  | **1.221 (1.095,1.362)** | **0.005** |  | -0.040 (-0.080,0.001) | 0.111 |  | 0.906 (0.798,1.028) | 0.251 |
| BzMA | -0.002 (-0.033,0.028) | 0.938 |  | 0.996 (0.902,1.100) | 0.972 |  | -0.029 (-0.070,0.013) | 0.236 |  | 0.933 (0.838,1.040) | 0.314 |
| 1PMA | 0.027 (0.002,0.051) | 0.057 |  | 1.060 (0.984,1.141) | 0.179 |  | -0.022 (-0.047,0.002) | 0.129 |  | 0.960 (0.898,1.025) | 0.314 |
| 2CoEMA | **0.088 (0.045,0.130)** | **0.001** |  | **1.238 (1.088,1.407)** | **0.008** |  | **0.075 (0.028,0.122)** | **0.010** |  | **1.220 (1.104,1.348)** | **0.002** |
| 2CyEMA | -0.017 (-0.043,0.009) | 0.258 |  | 0.999 (0.933,1.069) | 0.972 |  | 0.006 (-0.021,0.033) | 0.662 |  | 1.040 (0.973,1.113) | 0.324 |
| DHBMA | **0.117 (****0.047,0.187)** | **0.008** |  | **1.393 (****1.129,1.718)** | **0.008** |  | -0.016 (-0.076,0.043) | 0.627 |  | 1.026 (0.861,1.223) | 0.823 |
| 2HPMA | 0.037 (0.003,0.072) | 0.057 |  | 1.092 (0.997,1.195) | 0.102 |  | -0.016 (-0.054,0.023) | 0.474 |  | 1.008 (0.917,1.108) | 0.867 |
| 3HPMA | 0.018 (-0.024,0.060) | 0.483 |  | 1.093 (0.967,1.236) | 0.202 |  | 0.033 (-0.011,0.077) | 0.205 |  | **1.151 (1.032,1.284)** | **0.035** |
| MADA | **0.080 (0.025,0.135)** | **0.020** |  | **1.228 (1.056,1.428)** | **0.023** |  | 0.026 (-0.030,0.083) | 0.437 |  | 1.114 (0.956,1.298) | 0.289 |
| 4HBeMA | **0.046 (0.013,0.079)** | **0.022** |  | **1.177 (1.065,1.300)** | **0.008** |  | **0.060 (0.024,0.095)** | **0.007** |  | **1.165 (1.052,1.291)** | **0.023** |
| PhGA | **0.110 (0.058,0.163)** | **0.001** |  | **1.310 (1.129,1.520)** | **0.005** |  | -0.077 (-0.143,-0.012) | 0.071 |  | 0.923 (0.793,1.074) | 0.361 |
| 3HMPMA | 0.036 (-0.003,0.075) | 0.106 |  | **1.157 (1.036,1.293)** | **0.024** |  | **0.098 (0.050,0.145)** | **0.001** |  | **1.307 (1.145,1.491)** | **0.002** |

Survey-weighted multivariable linear and logistic regression models were used to assess associations of Ln-transformed VOCM concentrations with Z-score transformed KDMAgeAccel and AL, KDMAgeAccel>0, and median AL, adjusted for age, sex, BMI, race, marital status, education level, PIR, smoking status, drinking status, physical activity (MET-minutes/week), total energy intake (kcal), diabetes, hypertension, hyperlipidemia, and urinary creatinine. FDR correction was applied to all *P*-values across 16 VOCMs separately for each BA indicator. Bold font indicates *P*-FDR < 0.05.

Abbreviations: KDMAgeAccel, Klemera-Doubal method biological age acceleration; AL, allostatic load; 95% CI, 95% confidence interval; OR, odds ratio; *P*-FDR, false discovery rate corrected *P* value; 2MHA, 2-methylhippuric acid; 3&4MHA, 3- and 4-methylhippuric acid; 2CaEMA, N-acetyl-S-(2-carbamoylethyl)-L-cysteine; MCaMA, N-acetyl-S-(N-methylcarbamoyl)-L-cysteine; 2ATCA, 2-aminothiazoline-4-carboxylic acid; BzMA, N-acetyl-S-(benzyl)-L-cysteine; 1PMA, N-acetyl-S-(n-propyl)-L-cysteine; 2CoEMA, N-acetyl-S-(2-carboxyethyl)-L-cysteine; 2CyEMA, N-acetyl-S-(2-cyanoethyl)-L-cysteine; DHBMA, N-acetyl-S-(3,4-dihydroxybutyl)-L-cysteine; 2HPMA, N-acetyl-S-(2-hydroxypropyl)-L-cysteine; 3HPMA, N-acetyl-S-(3-hydroxypropyl)-L-cysteine; MADA, mandelic acid; 4HBeMA, N-acetyl-S-(4-hydroxy-2-butenyl)-L-cysteine; PhGA, phenylglyoxylic acid; 3HMPMA, N-acetyl-S-(3-hydroxypropyl-1-methyl)-L-cysteine.

**Table S12. Association of VOCMs with continuous and binary PhenoAgeAccel and HD in weighted generalized linear models**

|  | PhenoAgeAccel | |  | PhenoAgeAccel>0 | |  | HD | |  | Median HD | |
| --- | --- | --- | --- | --- | --- | --- | --- | --- | --- | --- | --- |
|  | β (95% CI) | *P*-FDR |  | OR (95% CI) | *P*-FDR |  | β (95% CI) | *P*-FDR |  | OR (95% CI) | *P*-FDR |
| 2MHA | 0.047 (-0.014,0.108) | 0.220 |  | 1.066 (0.914,1.243) | 0.635 |  | 0.024 (-0.036,0.084) | 0.535 |  | 1.075 (0.931,1.240) | 0.482 |
| 3&4MHA | 0.028 (-0.023,0.080) | 0.349 |  | 1.014 (0.868,1.186) | 0.922 |  | 0.039 (-0.013,0.091) | 0.428 |  | 1.132 (0.984,1.303) | 0.229 |
| 2CaEMA | 0.032 (-0.032,0.095) | 0.380 |  | 0.992 (0.801,1.230) | 0.939 |  | 0.019 (-0.051,0.089) | 0.655 |  | 1.009 (0.849,1.200) | 0.909 |
| MCaMA | -0.015 (-0.078,0.048) | 0.664 |  | 0.846 (0.704,1.015) | 0.184 |  | 0.035 (-0.027,0.097) | 0.466 |  | 1.029 (0.852,1.244) | 0.909 |
| 2ATCA | -0.014 (-0.057,0.030) | 0.589 |  | 1.032 (0.873,1.220) | 0.854 |  | 0.035 (-0.015,0.085) | 0.428 |  | 1.111 (0.962,1.283) | 0.283 |
| BzMA | -0.045 (-0.088,-0.002) | 0.151 |  | 0.825 (0.696,0.977) | 0.184 |  | -0.009 (-0.053,0.035) | 0.721 |  | 1.005 (0.916,1.103) | 0.909 |
| 1PMA | -0.004 (-0.034,0.025) | 0.771 |  | 1.068 (0.966,1.182) | 0.416 |  | 0.021 (-0.012,0.055) | 0.457 |  | 1.114 (1.034,1.200) | 0.117 |
| 2CoEMA | 0.052 (-0.010,0.113) | 0.186 |  | 1.191 (0.992,1.431) | 0.184 |  | 0.035 (-0.030,0.100) | 0.466 |  | 1.237 (1.020,1.500) | 0.162 |
| 2CyEMA | 0.039 (-0.004,0.081) | 0.160 |  | 1.050 (0.932,1.184) | 0.635 |  | 0.019 (-0.018,0.056) | 0.466 |  | 1.042 (0.934,1.162) | 0.592 |
| DHBMA | 0.121 (0.014,0.227) | 0.151 |  | 1.106 (0.786,1.556) | 0.783 |  | 0.074 (-0.027,0.175) | 0.428 |  | 1.310 (0.958,1.790) | 0.229 |
| 2HPMA | 0.034 (-0.023,0.091) | 0.319 |  | 1.034 (0.888,1.204) | 0.854 |  | 0.038 (-0.017,0.093) | 0.428 |  | 1.061 (0.904,1.245) | 0.592 |
| 3HPMA | 0.053 (0.001,0.105) | 0.151 |  | 1.206 (0.991,1.466) | 0.184 |  | 0.027 (-0.029,0.084) | 0.466 |  | 1.207 (1.012,1.440) | 0.162 |
| MADA | 0.068 (-0.004,0.140) | 0.160 |  | 1.117 (0.904,1.379) | 0.564 |  | 0.021 (-0.051,0.094) | 0.655 |  | 1.133 (0.917,1.399) | 0.405 |
| 4HBeMA | **0.100 (0.041,0.158)** | **0.036** |  | 1.207 (1.011,1.440) | 0.184 |  | 0.051 (-0.004,0.107) | 0.428 |  | 1.130 (0.955,1.336) | 0.283 |
| PhGA | 0.044 (-0.026,0.114) | 0.319 |  | 0.983 (0.794,1.216) | 0.922 |  | -0.003 (-0.074,0.068) | 0.925 |  | 0.980 (0.810,1.185) | 0.909 |
| 3HMPMA | **0.100 (0.036,0.164)** | **0.036** |  | 1.197 (0.987,1.450) | 0.184 |  | 0.066 (-0.000,0.131) | 0.428 |  | 1.238 (1.011,1.516) | 0.162 |

Survey-weighted multivariable linear and logistic regression models were used to assess associations of Ln-transformed VOCM concentrations with Z-score transformed PhenoAgeAccel and HD, PhenoAgeAccel>0, and median HD, adjusted for age, sex, BMI, race, marital status, education level, PIR, smoking status, drinking status, physical activity (MET-minutes/week), total energy intake (kcal), diabetes, hypertension, hyperlipidemia, and urinary creatinine. FDR correction was applied to all *P*-values across 16 VOCMs separately for each BA indicator. Bold font indicates *P*-FDR < 0.05.

Abbreviations: PhenoAgeAccel, phenotypic age acceleration; HD, homeostatic dysregulation; 95% CI, 95% confidence interval; OR, odds ratio; *P*-FDR, false discovery rate corrected *P* value; 2MHA, 2-methylhippuric acid; 3&4MHA, 3- and 4-methylhippuric acid; 2CaEMA, N-acetyl-S-(2-carbamoylethyl)-L-cysteine; MCaMA, N-acetyl-S-(N-methylcarbamoyl)-L-cysteine; 2ATCA, 2-aminothiazoline-4-carboxylic acid; BzMA, N-acetyl-S-(benzyl)-L-cysteine; 1PMA, N-acetyl-S-(n-propyl)-L-cysteine; 2CoEMA, N-acetyl-S-(2-carboxyethyl)-L-cysteine; 2CyEMA, N-acetyl-S-(2-cyanoethyl)-L-cysteine; DHBMA, N-acetyl-S-(3,4-dihydroxybutyl)-L-cysteine; 2HPMA, N-acetyl-S-(2-hydroxypropyl)-L-cysteine; 3HPMA, N-acetyl-S-(3-hydroxypropyl)-L-cysteine; MADA, mandelic acid; 4HBeMA, N-acetyl-S-(4-hydroxy-2-butenyl)-L-cysteine; PhGA, phenylglyoxylic acid; 3HMPMA, N-acetyl-S-(3-hydroxypropyl-1-methyl)-L-cysteine.

**Table S13. Association of VOCMs with KDM-BA and PA in weighted linear regression models**

|  | KDM-BA | |  | PA | |
| --- | --- | --- | --- | --- | --- |
|  | β (95% CI) | *P*-FDR |  | β (95% CI) | *P*-FDR |
| 2MHA | 0.457 (-0.028,0.941) | 0.079 |  | 0.454 (-0.254,1.162) | 0.261 |
| 3&4MHA | **0.661 (0.149,1.174)** | **0.017** |  | 0.749 (-0.070,1.569) | 0.113 |
| 2CaEMA | -0.374 (-1.199,0.451) | 0.392 |  | -0.758 (-2.031,0.514) | 0.280 |
| MCaMA | **1.470 (0.596,2.345)** | **0.003** |  | **2.672 (1.471,3.873)** | **0.000** |
| 2ATCA | **1.415 (0.712,2.119)** | **0.000** |  | 0.284 (-0.601,1.169) | 0.510 |
| BzMA | **1.567 (0.873,2.260)** | **0.000** |  | **2.401 (1.521,3.281)** | **0.000** |
| 1PMA | -0.034 (-0.602,0.535) | 0.905 |  | **-0.803 (-1.448,-0.159)** | **0.035** |
| 2CoEMA | **3.849 (3.124,4.575)** | **0.000** |  | **3.790 (2.610,4.970)** | **0.000** |
| 2CyEMA | **-0.640 (-1.086,-0.195)** | **0.009** |  | -0.424 (-1.081,0.234) | 0.261 |
| DHBMA | **5.944 (4.427,7.460)** | **0.000** |  | **7.450 (5.227,9.673)** | **0.000** |
| 2HPMA | **1.016 (0.241,1.791)** | **0.016** |  | 0.561 (-0.557,1.680) | 0.327 |
| 3HPMA | 0.630 (-0.082,1.343) | 0.093 |  | 0.688 (-0.541,1.918) | 0.292 |
| MADA | **2.465 (1.462,3.468)** | **0.000** |  | **1.814 (0.253,3.376)** | **0.045** |
| 4HBeMA | **2.057 (1.442,2.672)** | **0.000** |  | **2.636 (1.524,3.748)** | **0.000** |
| PhGA | **3.876 (2.764,4.989)** | **0.000** |  | **3.112 (1.363,4.861)** | **0.003** |
| 3HMPMA | **2.997 (2.192,3.801)** | **0.000** |  | **4.192 (2.868,5.516)** | **0.000** |

Survey-weighted multivariable linear regression models were used to assess associations of Ln-transformed VOCM concentrations with KDM-BA and PA, adjusted for sex, BMI, race, marital status, education level, PIR, smoking status, drinking status, physical activity (MET-minutes/week), total energy intake (kcal), diabetes, hypertension, hyperlipidemia, and urinary creatinine. FDR correction was applied to all *P*-values across 16 VOCMs separately for each BA indicator. Bold font indicates *P*-FDR < 0.05.

Abbreviations: KDM-BA, Klemera-Doubal method biological age; PA, phenotypic age; *P*-FDR, false discovery rate corrected *P* value; 2MHA, 2-methylhippuric acid; 3&4MHA, 3- and 4-methylhippuric acid; 2CaEMA, N-acetyl-S-(2-carbamoylethyl)-L-cysteine; MCaMA, N-acetyl-S-(N-methylcarbamoyl)-L-cysteine; 2ATCA, 2-aminothiazoline-4-carboxylic acid; BzMA, N-acetyl-S-(benzyl)-L-cysteine; 1PMA, N-acetyl-S-(n-propyl)-L-cysteine; 2CoEMA, N-acetyl-S-(2-carboxyethyl)-L-cysteine; 2CyEMA, N-acetyl-S-(2-cyanoethyl)-L-cysteine; DHBMA, N-acetyl-S-(3,4-dihydroxybutyl)-L-cysteine; 2HPMA, N-acetyl-S-(2-hydroxypropyl)-L-cysteine; 3HPMA, N-acetyl-S-(3-hydroxypropyl)-L-cysteine; MADA, mandelic acid; 4HBeMA, N-acetyl-S-(4-hydroxy-2-butenyl)-L-cysteine; PhGA, phenylglyoxylic acid; 3HMPMA, N-acetyl-S-(3-hydroxypropyl-1-methyl)-L-cysteine.

**Table S14.** **Interaction of VOCMs and dietary indexes on KDMAgeAccel and AL in weighted linear regression models**

| VOCMs | Dietary interaction | KDMAgeAccel | |  |  | AL | |  |
| --- | --- | --- | --- | --- | --- | --- | --- | --- |
|  |  | VOCMs term | Dietary term | *P*-FDR for  interaction |  | VOCMs term | Dietary term | *P*-FDR for  interaction |
|  |  | β (95% CI) | β (95% CI) |  |  | β (95% CI) | β (95% CI) |  |
| 2MHA |  |  |  |  |  |  |  |  |
|  | DII | **0.032 (0.002,0.061)** | **0.166 (0.063,0.269)*** | 0.066 |  | **0.040 (0.001,0.079)** | 0.055 (-0.066,0.175) | 0.979 |
|  | MEDI | **0.031 (0.001,0.061)** | **-0.180 (-0.268,-0.092)*** | **0.038** |  | 0.038 (-0.001,0.077) | -0.090 (-0.186,0.005) | 0.934 |
|  | HEI-2020 | **0.032 (0.003,0.062)** | **-0.155 (-0.248,-0.063)*** | 0.091 |  | **0.039 (0.001,0.078)** | -0.021 (-0.125,0.083) | 0.919 |
|  | AHEI-2010 | 0.029 (-0.001,0.060) | **-0.142 (-0.236,-0.048)*** | 0.189 |  | 0.037 (-0.002,0.076) | -0.056 (-0.160,0.047) | 0.934 |
|  | DASHI | 0.029 (-0.002,0.059) | **-0.145 (-0.249,-0.042)*** | 0.219 |  | 0.039 (-0.000,0.077) | -0.053 (-0.170,0.064) | 0.969 |
| 3&4MHA |  |  |  |  |  |  |  |  |
|  | DII | 0.004 (-0.024,0.032) | **0.213 (0.074,0.352)*** | 0.066 |  | 0.027 (-0.013,0.068) | 0.064 (-0.088,0.216) | 0.979 |
|  | MEDI | 0.004 (-0.024,0.033) | **-0.247 (-0.374,-0.120)*** | **0.033** |  | 0.027 (-0.013,0.067) | -0.075 (-0.195,0.045) | 0.988 |
|  | HEI-2020 | 0.004 (-0.025,0.033) | **-0.238 (-0.368,-0.108)*** | **0.049** |  | 0.027 (-0.013,0.067) | -0.012 (-0.145,0.122) | 0.919 |
|  | AHEI-2010 | 0.002 (-0.028,0.031) | **-0.181 (-0.313,-0.049)*** | 0.189 |  | 0.025 (-0.016,0.066) | -0.029 (-0.163,0.105) | 0.934 |
|  | DASHI | 0.002 (-0.028,0.031) | **-0.213 (-0.356,-0.071)*** | 0.149 |  | 0.028 (-0.012,0.067) | -0.036 (-0.180,0.109) | 0.969 |
| 2CaEMA |  |  |  |  |  |  |  |  |
|  | DII | -0.005 (-0.048,0.039) | **0.197 (0.082,0.312)*** | 0.066 |  | **-0.113 (-0.163,-0.063)*** | 0.127 (-0.009,0.263) | 0.979 |
|  | MEDI | -0.001 (-0.044,0.043) | **-0.251 (-0.369,-0.134)*** | **0.017** |  | **-0.109 (-0.157,-0.060)*** | **-0.194 (-0.309,-0.078)*** | 0.459 |
|  | HEI-2020 | -0.005 (-0.048,0.039) | **-0.176 (-0.290,-0.063)*** | 0.091 |  | **-0.114 (-0.163,-0.065)*** | **-0.176 (-0.283,-0.069)*** | 0.672 |
|  | AHEI-2010 | -0.002 (-0.045,0.041) | **-0.166 (-0.287,-0.045)*** | 0.189 |  | **-0.109 (-0.158,-0.060)*** | **-0.142 (-0.252,-0.033)*** | 0.934 |
|  | DASHI | -0.005 (-0.048,0.039) | **-0.206 (-0.333,-0.078)*** | 0.149 |  | **-0.112 (-0.161,-0.063)*** | **-0.171 (-0.287,-0.055)*** | 0.969 |
| MCaMA |  |  |  |  |  |  |  |  |
|  | DII | **-0.063 (-0.113,-0.013)*** | **0.266 (0.136,0.396)*** | **0.028** |  | **-0.053 (-0.100,-0.006)** | 0.102 (-0.079,0.284) | 0.979 |
|  | MEDI | **-0.067 (-0.116,-0.018)*** | **-0.383 (-0.509,-0.257)*** | **0.000** |  | **-0.055 (-0.100,-0.010)*** | **-0.235 (-0.401,-0.070)*** | 0.459 |
|  | HEI-2020 | **-0.068 (-0.118,-0.019)*** | **-0.317 (-0.451,-0.183)*** | **0.003** |  | **-0.057 (-0.103,-0.011)** | -0.153 (-0.317,0.010) | 0.919 |
|  | AHEI-2010 | **-0.067 (-0.116,-0.018)*** | **-0.249 (-0.390,-0.108)*** | 0.111 |  | **-0.055 (-0.101,-0.009)** | -0.142 (-0.295,0.010) | 0.934 |
|  | DASHI | **-0.074 (-0.124,-0.024)*** | **-0.300 (-0.445,-0.155)*** | **0.018** |  | **-0.061 (-0.107,-0.015)*** | **-0.186 (-0.357,-0.014)** | 0.969 |
| 2ATCA |  |  |  |  |  |  |  |  |
|  | DII | **0.046 (0.006,0.087)** | **0.218 (0.050,0.385)*** | 0.082 |  | -0.040 (-0.081,0.000) | 0.059 (-0.102,0.221) | 0.979 |
|  | MEDI | **0.045 (0.004,0.086)** | **-0.257 (-0.418,-0.096)*** | **0.038** |  | **-0.045 (-0.084,-0.005)** | **-0.176 (-0.336,-0.017)*** | 0.536 |
|  | HEI-2020 | **0.047 (0.006,0.088)** | **-0.194 (-0.378,-0.011)*** | 0.168 |  | **-0.042 (-0.082,-0.001)** | -0.078 (-0.228,0.072) | 0.919 |
|  | AHEI-2010 | **0.044 (0.003,0.085)** | **-0.223 (-0.380,-0.066)*** | 0.186 |  | **-0.045 (-0.085,-0.006)** | -0.137 (-0.275,0.002) | 0.934 |
|  | DASHI | **0.042 (0.001,0.084)** | **-0.189 (-0.339,-0.039)*** | 0.219 |  | **-0.047 (-0.087,-0.007)*** | -0.105 (-0.258,0.049) | 0.969 |
| BzMA |  |  |  |  |  |  |  |  |
|  | DII | 0.000 (-0.032,0.032) | **0.076 (0.004,0.148)** | 0.401 |  | -0.023 (-0.065,0.020) | 0.038 (-0.032,0.108) | 0.979 |
|  | MEDI | 0.005 (-0.027,0.037) | **-0.104 (-0.168,-0.039)*** | 0.378 |  | -0.021 (-0.064,0.022) | **-0.088 (-0.147,-0.029)*** | 0.934 |
|  | HEI-2020 | 0.003 (-0.028,0.035) | **-0.089 (-0.144,-0.034)*** | 0.277 |  | -0.021 (-0.063,0.021) | **-0.073 (-0.138,-0.008)** | 0.919 |
|  | AHEI-2010 | 0.002 (-0.029,0.033) | **-0.092 (-0.157,-0.027)*** | 0.336 |  | -0.018 (-0.061,0.024) | **-0.087 (-0.145,-0.030)*** | 0.972 |
|  | DASHI | 0.002 (-0.029,0.034) | **-0.089 (-0.152,-0.026)*** | 0.363 |  | -0.021 (-0.063,0.022) | **-0.087 (-0.157,-0.018)** | 0.969 |
| 1PMA |  |  |  |  |  |  |  |  |
|  | DII | **0.028 (0.003,0.052)** | **0.050 (0.002,0.099)** | 0.864 |  | -0.021 (-0.045,0.004) | **0.061 (0.004,0.119)** | 0.979 |
|  | MEDI | **0.031 (0.006,0.056)*** | **-0.084 (-0.126,-0.042)*** | 0.739 |  | -0.018 (-0.043,0.006) | **-0.089 (-0.142,-0.036)*** | 0.934 |
|  | HEI-2020 | **0.028 (0.003,0.053)** | **-0.071 (-0.116,-0.027)*** | 0.277 |  | -0.021 (-0.046,0.004) | **-0.095 (-0.146,-0.044)*** | 0.808 |
|  | AHEI-2010 | **0.028 (0.003,0.053)** | -0.050 (-0.102,0.001) | 0.673 |  | -0.021 (-0.045,0.004) | **-0.098 (-0.147,-0.050)*** | 0.970 |
|  | DASHI | **0.027 (0.002,0.052)** | -0.050 (-0.101,0.000) | 0.493 |  | -0.022 (-0.047,0.002) | **-0.095 (-0.152,-0.038)*** | 0.969 |
| 2CoEMA |  |  |  |  |  |  |  |  |
|  | DII | **0.085 (0.043,0.127)*** | 0.129 (-0.006,0.265) | 0.251 |  | **0.075 (0.027,0.124)*** | -0.004 (-0.183,0.176) | 0.979 |
|  | MEDI | **0.085 (0.043,0.127)*** | **-0.173 (-0.299,-0.046)*** | 0.157 |  | **0.074 (0.027,0.122)*** | -0.097 (-0.256,0.062) | 0.934 |
|  | HEI-2020 | **0.083 (0.041,0.125)*** | **-0.158 (-0.302,-0.014)*** | 0.217 |  | **0.074 (0.025,0.122)*** | -0.034 (-0.194,0.127) | 0.919 |
|  | AHEI-2010 | **0.084 (0.042,0.125)*** | -0.099 (-0.240,0.042) | 0.640 |  | **0.075 (0.026,0.124)*** | -0.021 (-0.170,0.128) | 0.934 |
|  | DASHI | **0.084 (0.042,0.126)*** | -0.095 (-0.248,0.057) | 0.655 |  | **0.075 (0.027,0.124)*** | -0.028 (-0.192,0.137) | 0.969 |
| 2CyEMA |  |  |  |  |  |  |  |  |
|  | DII | -0.018 (-0.045,0.009) | **0.061 (0.018,0.104)*** | 0.108 |  | 0.003 (-0.025,0.031) | 0.057 (-0.000,0.114) | 0.979 |
|  | MEDI | -0.016 (-0.042,0.011) | **-0.097 (-0.132,-0.061)*** | **0.038** |  | 0.004 (-0.024,0.031) | **-0.078 (-0.122,-0.034)*** | 0.988 |
|  | HEI-2020 | -0.015 (-0.042,0.012) | **-0.074 (-0.116,-0.032)*** | 0.091 |  | 0.005 (-0.023,0.032) | **-0.072 (-0.116,-0.029)*** | 0.919 |
|  | AHEI-2010 | -0.018 (-0.044,0.009) | **-0.071 (-0.112,-0.030)*** | 0.189 |  | 0.001 (-0.026,0.028) | **-0.088 (-0.132,-0.044)*** | 0.967 |
|  | DASHI | -0.017 (-0.044,0.009) | **-0.075 (-0.122,-0.029)*** | 0.232 |  | 0.003 (-0.024,0.030) | **-0.082 (-0.132,-0.031)*** | 0.969 |
| DHBMA |  |  |  |  |  |  |  |  |
|  | DII | **0.104 (0.037,0.171)*** | **0.248 (0.056,0.441)*** | 0.095 |  | -0.024 (-0.086,0.038) | 0.067 (-0.168,0.302) | 0.979 |
|  | MEDI | **0.104 (0.033,0.174)*** | **-0.299 (-0.509,-0.089)*** | **0.048** |  | -0.027 (-0.088,0.033) | **-0.239 (-0.427,-0.052)*** | 0.479 |
|  | HEI-2020 | **0.105 (0.038,0.172)*** | -0.141 (-0.351,0.070) | 0.390 |  | -0.029 (-0.090,0.031) | -0.136 (-0.355,0.083) | 0.919 |
|  | AHEI-2010 | **0.108 (0.039,0.177)*** | -0.141 (-0.369,0.086) | 0.580 |  | -0.024 (-0.085,0.038) | -0.099 (-0.317,0.119) | 0.972 |
|  | DASHI | **0.107 (0.038,0.175)*** | -0.164 (-0.383,0.055) | 0.405 |  | -0.026 (-0.087,0.034) | -0.161 (-0.386,0.063) | 0.969 |
| 2HPMA |  |  |  |  |  |  |  |  |
|  | DII | **0.038 (0.002,0.073)** | 0.086 (-0.016,0.189) | 0.485 |  | -0.011 (-0.050,0.029) | 0.030 (-0.092,0.152) | 0.979 |
|  | MEDI | **0.042 (0.006,0.078)*** | **-0.137 (-0.238,-0.036)*** | 0.285 |  | -0.009 (-0.048,0.031) | -0.089 (-0.204,0.026) | 0.934 |
|  | HEI-2020 | **0.037 (0.000,0.074)** | **-0.113 (-0.212,-0.013)*** | 0.314 |  | -0.014 (-0.054,0.027) | -0.095 (-0.205,0.014) | 0.919 |
|  | AHEI-2010 | **0.044 (0.008,0.080)*** | -0.060 (-0.158,0.038) | 0.994 |  | -0.005 (-0.046,0.036) | -0.083 (-0.185,0.018) | 0.972 |
|  | DASHI | **0.042 (0.005,0.080)** | -0.077 (-0.181,0.027) | 0.788 |  | -0.009 (-0.051,0.034) | -0.092 (-0.221,0.036) | 0.969 |
| 3HPMA |  |  |  |  |  |  |  |  |
|  | DII | 0.015 (-0.026,0.057) | **0.165 (0.003,0.327)** | 0.174 |  | 0.031 (-0.014,0.077) | -0.042 (-0.224,0.141) | 0.979 |
|  | MEDI | 0.016 (-0.025,0.058) | **-0.266 (-0.414,-0.117)*** | **0.037** |  | 0.031 (-0.012,0.075) | -0.116 (-0.285,0.053) | 0.934 |
|  | HEI-2020 | 0.014 (-0.027,0.055) | **-0.217 (-0.392,-0.041)*** | 0.123 |  | 0.030 (-0.014,0.075) | -0.060 (-0.231,0.111) | 0.919 |
|  | AHEI-2010 | 0.014 (-0.027,0.055) | **-0.177 (-0.340,-0.014)** | 0.216 |  | 0.032 (-0.012,0.076) | -0.048 (-0.196,0.099) | 0.934 |
|  | DASHI | 0.013 (-0.028,0.053) | -0.175 (-0.355,0.005) | 0.327 |  | 0.031 (-0.012,0.075) | -0.057 (-0.221,0.108) | 0.969 |
| MADA |  |  |  |  |  |  |  |  |
|  | DII | **0.072 (0.020,0.123)*** | 0.179 (-0.004,0.362) | 0.174 |  | 0.025 (-0.031,0.082) | 0.010 (-0.187,0.207) | 0.979 |
|  | MEDI | **0.072 (0.020,0.124)*** | **-0.263 (-0.422,-0.104)*** | **0.038** |  | 0.020 (-0.036,0.076) | **-0.172 (-0.321,-0.023)*** | 0.536 |
|  | HEI-2020 | **0.074 (0.023,0.125)*** | -0.153 (-0.346,0.039) | 0.318 |  | 0.023 (-0.034,0.079) | -0.087 (-0.244,0.071) | 0.919 |
|  | AHEI-2010 | **0.075 (0.024,0.126)*** | -0.137 (-0.314,0.040) | 0.504 |  | 0.026 (-0.030,0.083) | -0.077 (-0.248,0.093) | 0.972 |
|  | DASHI | **0.073 (0.022,0.124)*** | -0.156 (-0.327,0.015) | 0.363 |  | 0.023 (-0.033,0.078) | -0.118 (-0.284,0.047) | 0.969 |
| 4HBeMA |  |  |  |  |  |  |  |  |
|  | DII | **0.044 (0.012,0.077)*** | **0.086 (0.031,0.141)*** | 0.082 |  | **0.056 (0.020,0.093)*** | 0.050 (-0.027,0.127) | 0.979 |
|  | MEDI | **0.046 (0.014,0.078)*** | **-0.135 (-0.187,-0.083)*** | **0.031** |  | **0.058 (0.022,0.094)*** | **-0.089 (-0.151,-0.028)*** | 0.934 |
|  | HEI-2020 | **0.045 (0.012,0.078)*** | **-0.106 (-0.167,-0.045)*** | 0.091 |  | **0.057 (0.021,0.092)*** | **-0.078 (-0.145,-0.010)** | 0.919 |
|  | AHEI-2010 | **0.042 (0.010,0.074)*** | **-0.095 (-0.155,-0.035)*** | 0.189 |  | **0.056 (0.021,0.092)*** | **-0.072 (-0.134,-0.011)** | 0.934 |
|  | DASHI | **0.042 (0.010,0.074)*** | **-0.100 (-0.166,-0.034)*** | 0.219 |  | **0.057 (0.021,0.092)*** | **-0.078 (-0.151,-0.005)** | 0.969 |
| PhGA |  |  |  |  |  |  |  |  |
|  | DII | **0.104 (0.054,0.153)*** | **0.205 (0.034,0.375)*** | 0.108 |  | **-0.081 (-0.147,-0.014)** | 0.119 (-0.093,0.332) | 0.979 |
|  | MEDI | **0.105 (0.053,0.158)*** | **-0.241 (-0.408,-0.073)*** | 0.072 |  | **-0.081 (-0.147,-0.015)*** | **-0.208 (-0.382,-0.035)*** | 0.523 |
|  | HEI-2020 | **0.106 (0.055,0.158)*** | -0.130 (-0.312,0.052) | 0.390 |  | **-0.081 (-0.149,-0.013)** | -0.130 (-0.292,0.033) | 0.919 |
|  | AHEI-2010 | **0.112 (0.060,0.163)*** | -0.106 (-0.288,0.075) | 0.673 |  | **-0.072 (-0.140,-0.005)** | -0.094 (-0.272,0.083) | 0.972 |
|  | DASHI | **0.107 (0.055,0.158)*** | -0.154 (-0.329,0.022) | 0.376 |  | **-0.081 (-0.148,-0.014)*** | **-0.181 (-0.349,-0.012)** | 0.969 |
| 3HMPMA |  |  |  |  |  |  |  |  |
|  | DII | 0.034 (-0.005,0.073) | **0.201 (0.035,0.367)*** | 0.108 |  | **0.098 (0.049,0.147)*** | -0.038 (-0.250,0.173) | 0.979 |
|  | MEDI | 0.037 (-0.001,0.075) | **-0.276 (-0.437,-0.114)*** | **0.038** |  | **0.100 (0.053,0.147)*** | -0.113 (-0.298,0.072) | 0.934 |
|  | HEI-2020 | 0.036 (-0.002,0.074) | **-0.209 (-0.395,-0.023)*** | 0.168 |  | **0.100 (0.052,0.147)*** | -0.056 (-0.248,0.135) | 0.919 |
|  | AHEI-2010 | 0.033 (-0.004,0.071) | **-0.197 (-0.369,-0.025)*** | 0.189 |  | **0.100 (0.054,0.147)*** | -0.013 (-0.189,0.164) | 0.934 |
|  | DASHI | 0.034 (-0.003,0.070) | **-0.216 (-0.405,-0.027)*** | 0.219 |  | **0.102 (0.055,0.148)*** | -0.060 (-0.265,0.144) | 0.969 |

Survey-weighted multivariable linear regression models were used to assess interaction of Ln-transformed VOCM concentrations and Z-score transformed dietary indexes on Z-score transformed KDMAgeAccel and AL, adjusted for age, sex, BMI, race, marital status, education level, PIR, smoking status, drinking status, physical activity (MET-minutes/week), total energy intake (kcal), diabetes, hypertension, hyperlipidemia, and urinary creatinine. FDR correction was applied to all *P*-values across 16 VOCMs separately for each combination of a BA indicator and a dietary index. Bold font indicates effect estimates were statistically significant, *P*<0.05. Asterisks indicate *P*-FDR < 0.05.

Abbreviations: 95% CI, 95% confidence interval; KDMAgeAccel, Klemera-Doubal method biological age acceleration; AL, allostatic load; 2MHA, 2-methylhippuric acid; 3&4MHA, 3- and 4-methylhippuric acid; 2CaEMA, N-acetyl-S-(2-carbamoylethyl)-L-cysteine; MCaMA, N-acetyl-S-(N-methylcarbamoyl)-L-cysteine; 2ATCA, 2-aminothiazoline-4-carboxylic acid; BzMA, N-acetyl-S-(benzyl)-L-cysteine; 1PMA, N-acetyl-S-(n-propyl)-L-cysteine; 2CoEMA, N-acetyl-S-(2-carboxyethyl)-L-cysteine; 2CyEMA, N-acetyl-S-(2-cyanoethyl)-L-cysteine; DHBMA, N-acetyl-S-(3,4-dihydroxybutyl)-L-cysteine; 2HPMA, N-acetyl-S-(2-hydroxypropyl)-L-cysteine; 3HPMA, N-acetyl-S-(3-hydroxypropyl)-L-cysteine; MADA, mandelic acid; 4HBeMA, N-acetyl-S-(4-hydroxy-2-butenyl)-L-cysteine; PhGA, phenylglyoxylic acid; 3HMPMA, N-acetyl-S-(3-hydroxypropyl-1-methyl)-L-cysteine; DII, Dietary Inflammatory Index; MEDI, Mediterranean Diet Index; HEI-2020, Healthy Eating Index-2020; AHEI-2010, Alternative Healthy Eating Index-2010; DASHI, Dietary Approaches to Stop Hypertension Index.

**Table S15. Interaction of VOCMs and dietary indexes on PhenoAgeAccel and HD in weighted linear regression models**

| VOCMs | Dietary  interaction | PhenoAgeAccel | |  |  | HD | |  |
| --- | --- | --- | --- | --- | --- | --- | --- | --- |
|  |  | VOCMs term | Dietary term | *P*-FDR for  interaction |  | VOCMs term | Dietary term | *P*-FDR for  interaction |
|  |  | β (95% CI) | β (95% CI) |  |  | β (95% CI) | β (95% CI) |  |
| 2MHA |  |  |  |  |  |  |  |  |
|  | DII | 0.044 (-0.017,0.106) | 0.121 (-0.035,0.277) | 0.822 |  | 0.023 (-0.038,0.083) | 0.134 (-0.011,0.278) | 0.407 |
|  | MEDI | 0.044 (-0.019,0.108) | -0.027 (-0.158,0.104) | 0.928 |  | 0.023 (-0.038,0.084) | -0.009 (-0.112,0.095) | 0.839 |
|  | HEI-2020 | 0.043 (-0.019,0.106) | -0.032 (-0.157,0.093) | 0.949 |  | 0.022 (-0.038,0.083) | -0.005 (-0.112,0.103) | 0.605 |
|  | AHEI-2010 | 0.041 (-0.022,0.105) | -0.070 (-0.184,0.045) | 0.974 |  | 0.021 (-0.039,0.082) | -0.015 (-0.115,0.086) | 0.862 |
|  | DASHI | 0.043 (-0.020,0.106) | -0.044 (-0.176,0.087) | 0.969 |  | 0.022 (-0.039,0.083) | -0.005 (-0.115,0.105) | 0.790 |
| 3&4MHA |  |  |  |  |  |  |  |  |
|  | DII | 0.024 (-0.029,0.076) | 0.128 (-0.068,0.324) | 0.822 |  | 0.035 (-0.017,0.087) | 0.124 (-0.063,0.310) | 0.502 |
|  | MEDI | 0.026 (-0.026,0.079) | -0.027 (-0.199,0.146) | 0.928 |  | 0.038 (-0.014,0.090) | 0.001 (-0.153,0.154) | 0.839 |
|  | HEI-2020 | 0.024 (-0.028,0.076) | -0.063 (-0.238,0.113) | 0.949 |  | 0.037 (-0.015,0.089) | -0.052 (-0.220,0.117) | 0.824 |
|  | AHEI-2010 | 0.022 (-0.032,0.075) | -0.069 (-0.232,0.094) | 0.974 |  | 0.036 (-0.016,0.088) | 0.008 (-0.149,0.166) | 0.862 |
|  | DASHI | 0.024 (-0.029,0.077) | -0.084 (-0.267,0.099) | 0.969 |  | 0.037 (-0.016,0.089) | -0.031 (-0.206,0.145) | 0.931 |
| 2CaEMA |  |  |  |  |  |  |  |  |
|  | DII | 0.026 (-0.039,0.091) | 0.098 (-0.069,0.265) | 0.854 |  | 0.013 (-0.057,0.083) | **0.176 (0.001,0.351)** | 0.307 |
|  | MEDI | 0.032 (-0.033,0.096) | -0.047 (-0.214,0.120) | 0.997 |  | 0.019 (-0.052,0.090) | -0.066 (-0.268,0.136) | 0.839 |
|  | HEI-2020 | 0.029 (-0.035,0.093) | -0.061 (-0.215,0.092) | 0.949 |  | 0.016 (-0.056,0.087) | -0.119 (-0.301,0.063) | 0.605 |
|  | AHEI-2010 | 0.032 (-0.032,0.095) | -0.066 (-0.217,0.086) | 0.974 |  | 0.018 (-0.052,0.088) | -0.067 (-0.252,0.118) | 0.862 |
|  | DASHI | 0.030 (-0.034,0.093) | -0.083 (-0.236,0.070) | 0.969 |  | 0.016 (-0.055,0.088) | -0.105 (-0.279,0.070) | 0.790 |
| MCaMA |  |  |  |  |  |  |  |  |
|  | DII | -0.025 (-0.089,0.040) | 0.137 (-0.027,0.301) | 0.822 |  | 0.027 (-0.036,0.090) | **0.221 (0.071,0.372)** | 0.255 |
|  | MEDI | -0.021 (-0.084,0.043) | -0.115 (-0.269,0.038) | 0.928 |  | 0.030 (-0.032,0.093) | **-0.189 (-0.358,-0.020)** | 0.839 |
|  | HEI-2020 | -0.027 (-0.091,0.037) | -0.110 (-0.258,0.038) | 0.949 |  | 0.026 (-0.036,0.088) | **-0.196 (-0.364,-0.028)** | 0.605 |
|  | AHEI-2010 | -0.026 (-0.090,0.039) | -0.076 (-0.220,0.068) | 0.974 |  | 0.024 (-0.040,0.088) | -0.131 (-0.309,0.048) | 0.862 |
|  | DASHI | -0.029 (-0.092,0.035) | -0.107 (-0.272,0.058) | 0.969 |  | 0.022 (-0.041,0.085) | -0.153 (-0.330,0.024) | 0.790 |
| 2ATCA |  |  |  |  |  |  |  |  |
|  | DII | -0.015 (-0.059,0.029) | 0.089 (-0.109,0.287) | 0.941 |  | 0.035 (-0.016,0.086) | 0.034 (-0.197,0.266) | 0.894 |
|  | MEDI | -0.017 (-0.062,0.028) | -0.109 (-0.318,0.100) | 0.928 |  | 0.033 (-0.018,0.085) | 0.040 (-0.216,0.295) | 0.839 |
|  | HEI-2020 | -0.016 (-0.060,0.028) | -0.051 (-0.242,0.140) | 0.949 |  | 0.034 (-0.017,0.085) | 0.037 (-0.182,0.255) | 0.605 |
|  | AHEI-2010 | -0.020 (-0.064,0.024) | -0.131 (-0.309,0.046) | 0.974 |  | 0.033 (-0.018,0.084) | 0.002 (-0.235,0.238) | 0.862 |
|  | DASHI | -0.019 (-0.063,0.025) | -0.094 (-0.281,0.093) | 0.969 |  | 0.034 (-0.017,0.085) | 0.043 (-0.170,0.255) | 0.790 |
| BzMA |  |  |  |  |  |  |  |  |
|  | DII | -0.041 (-0.086,0.005) | **0.102 (0.020,0.185)** | 0.822 |  | -0.006 (-0.050,0.038) | 0.085 (-0.008,0.179) | 0.502 |
|  | MEDI | -0.041 (-0.085,0.004) | -0.036 (-0.131,0.060) | 0.928 |  | -0.008 (-0.052,0.037) | -0.046 (-0.148,0.055) | 0.839 |
|  | HEI-2020 | -0.038 (-0.084,0.009) | **-0.089 (-0.166,-0.011)** | 0.949 |  | -0.006 (-0.048,0.037) | -0.070 (-0.154,0.014) | 0.605 |
|  | AHEI-2010 | -0.037 (-0.083,0.009) | **-0.086 (-0.166,-0.007)** | 0.974 |  | -0.006 (-0.049,0.038) | -0.077 (-0.171,0.018) | 0.862 |
|  | DASHI | -0.038 (-0.084,0.008) | -0.065 (-0.149,0.020) | 0.973 |  | -0.005 (-0.048,0.038) | -0.062 (-0.144,0.019) | 0.886 |
| 1PMA |  |  |  |  |  |  |  |  |
|  | DII | -0.002 (-0.031,0.028) | 0.067 (-0.010,0.144) | 0.822 |  | 0.023 (-0.011,0.057) | 0.064 (-0.006,0.134) | 0.894 |
|  | MEDI | -0.002 (-0.031,0.026) | -0.035 (-0.107,0.038) | 0.928 |  | 0.024 (-0.009,0.057) | -0.064 (-0.137,0.009) | 0.839 |
|  | HEI-2020 | -0.004 (-0.033,0.026) | -0.059 (-0.129,0.011) | 0.949 |  | 0.023 (-0.010,0.056) | -0.058 (-0.139,0.022) | 0.605 |
|  | AHEI-2010 | -0.002 (-0.033,0.028) | -0.059 (-0.139,0.021) | 0.974 |  | 0.021 (-0.012,0.054) | **-0.078 (-0.149,-0.006)** | 0.862 |
|  | DASHI | -0.004 (-0.034,0.026) | -0.057 (-0.146,0.031) | 0.969 |  | 0.021 (-0.012,0.054) | -0.067 (-0.153,0.019) | 0.790 |
| 2CoEMA |  |  |  |  |  |  |  |  |
|  | DII | 0.051 (-0.010,0.112) | 0.078 (-0.074,0.230) | 0.974 |  | 0.033 (-0.032,0.097) | 0.146 (-0.053,0.345) | 0.479 |
|  | MEDI | 0.051 (-0.011,0.113) | 0.002 (-0.156,0.161) | 0.928 |  | 0.033 (-0.032,0.099) | -0.074 (-0.255,0.107) | 0.839 |
|  | HEI-2020 | 0.051 (-0.011,0.112) | -0.021 (-0.200,0.159) | 0.949 |  | 0.032 (-0.034,0.097) | -0.083 (-0.265,0.100) | 0.643 |
|  | AHEI-2010 | 0.050 (-0.014,0.113) | -0.011 (-0.168,0.147) | 0.974 |  | 0.030 (-0.036,0.096) | -0.071 (-0.279,0.137) | 0.862 |
|  | DASHI | 0.052 (-0.011,0.114) | 0.005 (-0.158,0.168) | 0.969 |  | 0.032 (-0.034,0.098) | -0.045 (-0.213,0.123) | 0.971 |
| 2CyEMA |  |  |  |  |  |  |  |  |
|  | DII | 0.036 (-0.008,0.080) | **0.078 (0.014,0.141)** | 0.822 |  | 0.018 (-0.020,0.056) | **0.070 (0.010,0.130)** | 0.255 |
|  | MEDI | 0.039 (-0.006,0.083) | -0.050 (-0.109,0.010) | 0.928 |  | 0.019 (-0.019,0.057) | -0.025 (-0.074,0.024) | 0.839 |
|  | HEI-2020 | 0.038 (-0.005,0.082) | **-0.075 (-0.131,-0.020)** | 0.949 |  | 0.020 (-0.018,0.058) | -0.046 (-0.099,0.007) | 0.605 |
|  | AHEI-2010 | 0.035 (-0.009,0.080) | **-0.082 (-0.136,-0.029)** | 0.974 |  | 0.017 (-0.021,0.055) | -0.055 (-0.111,0.000) | 0.862 |
|  | DASHI | 0.038 (-0.006,0.082) | **-0.079 (-0.141,-0.017)** | 0.969 |  | 0.019 (-0.019,0.057) | **-0.062 (-0.117,-0.007)** | 0.790 |
| DHBMA |  |  |  |  |  |  |  |  |
|  | DII | 0.107 (-0.001,0.215) | 0.133 (-0.122,0.387) | 0.822 |  | 0.058 (-0.043,0.159) | 0.240 (-0.095,0.576) | 0.407 |
|  | MEDI | **0.120 (0.007,0.234)** | 0.017 (-0.314,0.347) | 0.928 |  | 0.070 (-0.033,0.174) | -0.059 (-0.470,0.352) | 0.839 |
|  | HEI-2020 | 0.116 (-0.001,0.232) | 0.008 (-0.316,0.333) | 0.949 |  | 0.060 (-0.050,0.170) | -0.162 (-0.531,0.207) | 0.605 |
|  | AHEI-2010 | **0.122 (0.007,0.237)** | 0.075 (-0.235,0.386) | 0.974 |  | 0.072 (-0.031,0.176) | 0.009 (-0.403,0.421) | 0.862 |
|  | DASHI | **0.126 (0.012,0.239)** | 0.051 (-0.271,0.372) | 0.969 |  | 0.072 (-0.036,0.181) | -0.022 (-0.367,0.323) | 0.931 |
| 2HPMA |  |  |  |  |  |  |  |  |
|  | DII | 0.042 (-0.019,0.102) | 0.034 (-0.134,0.203) | 0.822 |  | 0.042 (-0.014,0.098) | 0.066 (-0.105,0.238) | 0.950 |
|  | MEDI | 0.048 (-0.008,0.104) | 0.103 (-0.049,0.255) | 0.407 |  | 0.046 (-0.008,0.101) | 0.076 (-0.077,0.230) | 0.839 |
|  | HEI-2020 | 0.043 (-0.014,0.100) | 0.016 (-0.133,0.164) | 0.949 |  | 0.043 (-0.011,0.098) | 0.023 (-0.121,0.167) | 0.605 |
|  | AHEI-2010 | 0.057 (-0.002,0.116) | 0.057 (-0.073,0.186) | 0.253 |  | 0.053 (-0.006,0.111) | 0.039 (-0.098,0.176) | 0.862 |
|  | DASHI | 0.056 (-0.002,0.114) | 0.054 (-0.081,0.190) | 0.337 |  | 0.052 (-0.004,0.109) | 0.026 (-0.124,0.175) | 0.790 |
| 3HPMA |  |  |  |  |  |  |  |  |
|  | DII | 0.049 (-0.004,0.102) | 0.112 (-0.083,0.307) | 0.822 |  | 0.023 (-0.033,0.079) | **0.204 (0.015,0.392)** | 0.307 |
|  | MEDI | 0.051 (-0.001,0.104) | -0.003 (-0.168,0.162) | 0.928 |  | 0.026 (-0.031,0.084) | -0.107 (-0.291,0.076) | 0.839 |
|  | HEI-2020 | 0.051 (-0.002,0.103) | -0.064 (-0.272,0.143) | 0.949 |  | 0.025 (-0.032,0.081) | -0.121 (-0.312,0.071) | 0.605 |
|  | AHEI-2010 | 0.048 (-0.005,0.101) | -0.097 (-0.269,0.076) | 0.974 |  | 0.022 (-0.036,0.080) | -0.148 (-0.367,0.071) | 0.862 |
|  | DASHI | 0.048 (-0.005,0.101) | -0.106 (-0.286,0.073) | 0.969 |  | 0.022 (-0.036,0.080) | -0.129 (-0.295,0.037) | 0.790 |
| MADA |  |  |  |  |  |  |  |  |
|  | DII | 0.064 (-0.007,0.135) | 0.124 (-0.125,0.372) | 0.822 |  | 0.016 (-0.054,0.087) | 0.212 (-0.014,0.437) | 0.345 |
|  | MEDI | 0.065 (-0.008,0.137) | -0.102 (-0.355,0.151) | 0.928 |  | 0.017 (-0.055,0.089) | -0.142 (-0.365,0.080) | 0.839 |
|  | HEI-2020 | 0.066 (-0.007,0.139) | -0.037 (-0.281,0.206) | 0.949 |  | 0.017 (-0.054,0.088) | -0.133 (-0.368,0.101) | 0.605 |
|  | AHEI-2010 | 0.066 (-0.006,0.138) | -0.059 (-0.275,0.158) | 0.974 |  | 0.017 (-0.055,0.088) | -0.088 (-0.296,0.120) | 0.862 |
|  | DASHI | 0.064 (-0.009,0.137) | -0.098 (-0.343,0.146) | 0.969 |  | 0.015 (-0.059,0.090) | -0.118 (-0.333,0.096) | 0.790 |
| 4HBeMA |  |  |  |  |  |  |  |  |
|  | DII | **0.095 (0.036,0.154)*** | **0.088 (0.010,0.167)** | 0.822 |  | 0.048 (-0.008,0.104) | **0.090 (0.016,0.163)** | 0.297 |
|  | MEDI | **0.098 (0.039,0.157)*** | -0.045 (-0.117,0.027) | 0.997 |  | 0.050 (-0.006,0.107) | -0.024 (-0.086,0.037) | 0.839 |
|  | HEI-2020 | **0.095 (0.037,0.153)*** | -0.065 (-0.142,0.013) | 0.949 |  | 0.049 (-0.007,0.106) | -0.052 (-0.125,0.020) | 0.605 |
|  | AHEI-2010 | **0.094 (0.036,0.152)*** | **-0.075 (-0.140,-0.009)** | 0.974 |  | 0.048 (-0.009,0.104) | -0.049 (-0.121,0.023) | 0.878 |
|  | DASHI | **0.096 (0.037,0.154)*** | -0.072 (-0.151,0.008) | 0.969 |  | 0.048 (-0.008,0.105) | -0.053 (-0.121,0.016) | 0.931 |
| PhGA |  |  |  |  |  |  |  |  |
|  | DII | 0.039 (-0.028,0.105) | 0.244 (-0.030,0.519) | 0.822 |  | -0.010 (-0.080,0.061) | **0.317 (0.040,0.594)** | 0.289 |
|  | MEDI | 0.042 (-0.028,0.111) | -0.088 (-0.336,0.160) | 0.928 |  | -0.008 (-0.081,0.065) | -0.159 (-0.441,0.124) | 0.839 |
|  | HEI-2020 | 0.040 (-0.030,0.109) | -0.050 (-0.294,0.195) | 0.949 |  | -0.009 (-0.082,0.064) | -0.141 (-0.420,0.137) | 0.605 |
|  | AHEI-2010 | 0.044 (-0.026,0.113) | -0.094 (-0.331,0.143) | 0.974 |  | -0.007 (-0.081,0.068) | -0.114 (-0.398,0.170) | 0.862 |
|  | DASHI | 0.039 (-0.028,0.105) | -0.130 (-0.368,0.108) | 0.969 |  | -0.010 (-0.084,0.064) | -0.137 (-0.393,0.118) | 0.790 |
| 3HMPMA |  |  |  |  |  |  |  |  |
|  | DII | **0.098 (0.033,0.162)*** | 0.119 (-0.102,0.341) | 0.822 |  | 0.063 (-0.002,0.128) | 0.167 (-0.051,0.385) | 0.407 |
|  | MEDI | **0.101 (0.039,0.163)*** | 0.003 (-0.157,0.164) | 0.928 |  | **0.066 (0.000,0.132)** | 0.005 (-0.167,0.176) | 0.839 |
|  | HEI-2020 | **0.101 (0.039,0.163)*** | -0.067 (-0.259,0.126) | 0.949 |  | **0.066 (0.000,0.131)** | -0.072 (-0.284,0.139) | 0.740 |
|  | AHEI-2010 | **0.097 (0.034,0.161)*** | -0.063 (-0.228,0.102) | 0.974 |  | 0.065 (-0.001,0.130) | -0.019 (-0.222,0.184) | 0.862 |
|  | DASHI | **0.102 (0.041,0.163)*** | -0.063 (-0.252,0.125) | 0.973 |  | **0.067 (0.002,0.132)** | -0.036 (-0.208,0.137) | 0.931 |

Survey-weighted multivariable linear regression models were used to assess interaction of Ln-transformed VOCM concentrations and Z-score transformed dietary indexes on Z-score transformed PhenoAgeAccel and HD, adjusted for age, sex, BMI, race, marital status, education level, PIR, smoking status, drinking status, physical activity (MET-minutes/week), total energy intake (kcal), diabetes, hypertension, hyperlipidemia, and urinary creatinine. FDR correction was applied to all *P*-values across 16 VOCMs separately for each combination of a BA indicator and a dietary index. Bold font indicates effect estimates were statistically significant, *P*<0.05. Asterisks indicate *P*-FDR < 0.05.

Abbreviations: PhenoAgeAccel, phenotypic age acceleration; HD, homeostatic dysregulation; 2MHA, 2-methylhippuric acid; 3&4MHA, 3- and 4-methylhippuric acid; 2CaEMA, N-acetyl-S-(2-carbamoylethyl)-L-cysteine; MCaMA, N-acetyl-S-(N-methylcarbamoyl)-L-cysteine; 2ATCA, 2-aminothiazoline-4-carboxylic acid; BzMA, N-acetyl-S-(benzyl)-L-cysteine; 1PMA, N-acetyl-S-(n-propyl)-L-cysteine; 2CoEMA, N-acetyl-S-(2-carboxyethyl)-L-cysteine; 2CyEMA, N-acetyl-S-(2-cyanoethyl)-L-cysteine; DHBMA, N-acetyl-S-(3,4-dihydroxybutyl)-L-cysteine; 2HPMA, N-acetyl-S-(2-hydroxypropyl)-L-cysteine; 3HPMA, N-acetyl-S-(3-hydroxypropyl)-L-cysteine; MADA, mandelic acid; 4HBeMA, N-acetyl-S-(4-hydroxy-2-butenyl)-L-cysteine; PhGA, phenylglyoxylic acid; 3HMPMA, N-acetyl-S-(3-hydroxypropyl-1-methyl)-L-cysteine; DII, Dietary Inflammatory Index; MEDI, Mediterranean Diet Index; HEI-2020, Healthy Eating Index-2020; AHEI-2010, Alternative Healthy Eating Index-2010; DASHI, Dietary Approaches to Stop Hypertension Index.
